# Countries’ vulnerability to food supply disruptions caused by the Russia–Ukraine war from a trade dependency perspective

**DOI:** 10.1038/s41598-023-43883-4

**Published:** 2023-10-03

**Authors:** Zhengyang Zhang, Meshal J. Abdullah, Guochang Xu, Kazuyo Matsubae, Xianlai Zeng

**Affiliations:** 1 Graduate School of Environmental Studies, Tohoku University, Sendai, 980-0872 Japan; 2 Kuwait Environment Public Authority, 12066 Kuwait City, Kuwait; 3 School of Environment, Tsinghua University, Beijing, 100084 China; 4 Material Cycles Division, National Institute for Environmental Studies, Onogawa 16-2, Tsukuba, 305-8506 Japan; 5 Research Institute for Humanity and Nature, Kyoto, 603-8047 Japan

**Keywords:** Socioeconomic scenarios, Sustainability

## Abstract

Disruptions of key food and fertilizer exports from Russia and Ukraine have exposed many countries to challenges accessing some commodities since these countries’ war began. We evaluated the short-term, external, and direct impacts of disruptions of six food commodities and three types of fertilizer supplies from Russia and Ukraine on food access for all trading partners of the two countries by applying a set of trade and socioeconomic indicators. We found that the external food supplies of 279 countries and territories were affected to varying degrees; 24 countries—especially Georgia, Armenia, Kazakhstan, Azerbaijan, and Mongolia—are extremely vulnerable because they depend almost entirely on a variety of food imports from Russia and Ukraine. Access to fertilizers was affected in 136 countries and territories, particularly Estonia (potassic fertilizer), Mongolia (nitrogenous fertilizers), Kazakhstan (mixed fertilizers), and Brazil, the United States, China, and India (all types of fertilizers). An integrated assessment of countries’ import types, purchasing power parity per capita, and populations indicated that the Democratic Republic of the Congo, Ethiopia, Egypt, and Pakistan are most vulnerable to such supply disruptions. Development of research into diversification and decentralization strategies for food access is needed to guide stable food supply policies.

## Introduction

The ongoing conflict in Ukraine, combined with international sanctions against Russian grain and fertilizer exports, has affected global food supply chains, dealing a crippling blow to global food security^1^. Grain and fertilizers are essential components of global food security. Grain supplies a major proportion of the world’s dietary energy and nutrition needs^2^—especially wheat, which contributes about one-fifth of the total dietary calories and proteins worldwide^3^. The use of fertilizers containing nitrogen (N), phosphorus (P), and potassium (K) can boost average crop yields by 30–50% in intensive agricultural systems^4,5^, thus helping to sustain the ability of crop production to nourish the world’s growing population.

Russia and Ukraine are major global suppliers of grains and edible oils. Of global exports in 2019, they together produced about 30% of durum wheat, 25% of non-durum wheat and meslin, 20% of unprocessed barley, 18% of maize excluding seed corn, 25% of refined sunflower oil, and 71% of crude sunflower oil (Fig. S1, Table S1)^6^. In total, their exports represent nearly 12% of the total calories traded in the world^7,8^. Russia is also the world’s largest exporter of fertilizers; its exports of nitrogenous, potassic, and mixed fertilizers accounted for 13%, 16%, and 15%, respectively, of global markets in 2019 (Fig. S1, Table S1)^6^. Such a high concentration of grain and fertilizer supplies has the potential to expose many countries—particularly import-dependent countries—to increased vulnerability to food insecurity during this conflict, because the extent to which a country connects to world markets through trade is one of the major factors affecting its food security^9^.

The impact of the Russia–Ukraine war on food security has been studied from several perspectives and approaches. Much attention has been focused on the consequences of nutritional insecurity^10–14^; increasing prices for global energy, fertilizers, and food^11,12,14–19^; changes in food imports^16^ and exports^20^; and welfare losses^16,18^ at a regional or global scale, or both. For example, Alexander et al.^12^ projected that higher energy prices combined with food export restrictions from Russia and Ukraine could increase food costs by 60–100% in 2023 from 2021 levels, leading to undernourishment for 61–107 million people in 2023 and net annual additional deaths of 0.42–1.01 million people if the associated dietary patterns are maintained. The greatest impact is in Sub-Saharan Africa, with a projected 307 additional deaths per million per year. These studies have also revealed that food security is most vulnerable in low-income countries and countries heavily dependent on wheat imports from Ukraine. The major approaches used in the abovementioned studies (i.e., the land system modular model^12^, multi-regional input–output model^10^, and computable general equilibrium model^11,16,18,20^) allow for a good understanding of the dynamic changes in human health, macro-economics, and global trade caused by the war, as well as of the inter-country and inter-sectoral impacts of the war through food supply chains and the relative long-term impacts on food security. However, the geographic coverage and agricultural sector breakdown in these studies have been limited. For instance, many African countries were either not analyzed or combined into one region, and many specific agri-commodities were aggregated into one commodity sector (e.g., durum and non-durum wheat, wheat flour, wheat meal, etc. were aggregated into the wheat sector) despite the limited types of wheat products exported from Russia and Ukraine. This may lower the accuracy of information on the short-term impacts of the war on food security at a more detailed country-commodity level.

Other analyses have shed light on the short-term impacts on food security by country and commodity category, mainly by examining countries’ import dependencies of selected food commodities from Russia and Ukraine and taking into account different factors (e.g., the Shannon Diversity Index, cereal production, stocks and prices, diet cost, prevalences of food insecurity and undernourishment). Such analyses have been conducted mostly for cereal products in some Middle Eastern countries^21–24^, Slovenia^25^, and a limited range of other countries^26,27^. However, without a detailed overall international comparison of war-related food insecurity, some vulnerable countries might be overlooked, or the extent of vulnerability in some countries might be overestimated. Studies that have attempted to cover all countries include those that have identified food security vulnerability without a clear commodity classification^28^; quantified weak spots in cereal networks across 238 countries (if a country imported multiple products, it was counted multiple times)^29^; and estimated import dependence on wheat from Russia as well as dependence on wheat, maize, and seed oils from Ukraine across 226 countries through a products network approach, despite the difficulties in comparing the dependency on the same commodity from the two countries owing to product heterogeneity^30^. One of the most important agricultural inputs—different types of fertilizers—has not been investigated in a similar manner, leaving questions about the consequences of global fertilizer supply disruptions unanswered.

To fill in the gaps of previous research and with a focus on the access pillar of food security, we investigated the short-term, external, and direct impacts of food and fertilizer supply disruptions in Russia and Ukraine on food access for all trading partners of the two countries by applying a set of trade and socioeconomic indicators. The novelty lies in creating a detailed overview of the impacts on a country-by-country and commodity-by-commodity basis that has a global scope, and thereby including heretofore unrecognized, as well as newly emerged, areas vulnerable to disruptions of food and fertilizer supplies from Russia and Ukraine.

## Methods

To assess the short-term external supply risks of food and fertilizer shocks to all trading partners of Russia and Ukraine, we first selected a number of commodities exported from the two countries. Then, we identified a set of indicators of trade dependency and socioeconomic factors and quantified them by using actual values for 2020.

### Selection of target commodities

The nine commodities investigated were durum wheat, non-durum wheat and meslin, unprocessed barley, maize (excluding seed corn), crude sunflower oil, and refined sunflower oil exported from Russia and Ukraine, and nitrogenous fertilizers, potassic fertilizers, and mixed fertilizers exported from Russia. According to trade statistics, these commodities were the top six most exported grains and edible oils from both countries and the top three exported fertilizers from Russia, by trade value, in 2019^54^. Data were obtained from the Atlas of Economic Complexity Dataverse. Note that the Atlas dataset’s raw trade data on goods are derived from countries reporting to the United Nations Statistical Division (UN Comtrade). The Atlas dataset rectifies inconsistent trade data and cross-references corrected values of the reported exports and imports of countries to generate reliable estimates of trade flows among 250 countries and territories for more than 6000 goods^54^.

### Vulnerability assessment indicators

The first indicator to assess vulnerability was the trading partners’ dependency on exports of each commodity from Russia and Ukraine. The indicator is a combination of import quantity, import shares from Russia and Ukraine in a country’s total imports of each target commodity (ranging from 0.0 to 1.0), and the Herfindahl–Hirschman Index (HHI), a measure of market concentration commonly used in economics and antitrust analysis^55,56^. The HHI has also been used as an indicator in supply risk assessment^21,57^ and energy security^58^. Its value also ranged from 0.0 to 1.0 in this study. If a country imports a commodity from a large number of countries having relatively small market shares, the HHI is low, indicating strong competition in that country’s market. In contrast, as HHI approaches 1.0, market conditions are less competitive and are dominated by a few large suppliers^59^. A higher HHI value, a higher import share, and a larger quantity of imports indicate a greater possibility that, other things equal, a country’s commodity imports are more dependent on Russia or Ukraine, thereby implying a greater vulnerability to supply disruptions.

The second indicator integrates a country’s total number of import types of relevant commodities, PPP per capita, and population size. The demand for individual products generally depends on a number of variables, including population and per capita income (rather than total income)^60^. Moreover, population and income growth key drivers of food demand in food security studies for the period 2010–2050^61^. Previous studies of global food security have also suggested that purchasing power can reduce the capacity of consumers to access sufficient food^62,63^, and affordability is the primary issue for food security^12^. Therefore, we first chose a country’s total population, rather than other dynamic demographic factors, to emphasize the comparison of immediate and overall food demand levels among countries. We then chose the PPP per capita, which allows inter-country comparisons of levels of average economic well-being^64^. A higher PPP per capita indicates that individuals are relatively more capable of purchasing goods and services to meet demand, even in the face of rising prices. Therefore, a greater number of import types of commodities from Russia and Ukraine, a larger population, and a lower PPP per capita all contribute to the exposure of a country to greater vulnerability to supply disruptions, as compared with a country with a more limited import types, smaller population, and higher PPP per capita.

### Calculation of import shares

The import of a given commodity (e.g., durum wheat) from Russia or Ukraine as a proportion of a trading partner’s total imports was calculated as follows:

1 $$\begin{array}{c}{D}_{i,RUS}^{k}\left(t\right)=\frac{{Q}_{i,RUS}^{k}\left(t\right)}{{\sum }_{j}\left({Q}_{i,j}^{k}\left(t\right)\right)}\end{array}$$

2 $$\begin{array}{c}{D}_{i,UKR}^{k}\left(t\right)=\frac{{Q}_{i,UKR}^{k}\left(t\right)}{{\sum }_{j}\left({Q}_{i,j}^{k}\left(t\right)\right)}\end{array}$$

where ${D}_{i,RUS}^{k}\left(t\right)$ and ${D}_{i,UKR}^{k}\left(t\right)$ denote country $k$’s share of commodity $i$ imported from Russia and Ukraine, respectively, in year $t$; ${Q}_{i,RUS}^{k}\left(t\right)$ and ${Q}_{i,UKR}^{k}\left(t\right)$ are country $k$’s import quantity of commodity $i$ from Russia and Ukraine, respectively, in year $t$; and ${Q}_{i,j}^{k}\left(t\right)$ is the quantity of commodity $i$ imported by country $k$ from each trading partner $j$ in year $t$.

### Calculation of the HHI

The HHI is calculated by incorporating the market shares of all firms in a market^55,56^. If ${x}_{i}$ is the quantity of a country’s trade in a specific commodity with trading partner $i$ in a given year, $n$ partners exist in the market, and $x$ is the country’s total trade, HHI can be obtained by summing the squares of the market share percentages of all trading partners of a country for a particular commodity, as follows:

3 $$\begin{array}{c}HHI=\sum_{i=1}^{n}{\left({x}_{i}/x\right)}^{2}\end{array}$$

The data used in Eqs. (1)–(3) were obtained from the Chatham House Resource Trade Database (CHRTD), and the International Merchandise Trade Statistics was the original data source for CHRTD. CHRTD overcomes the difficulty of amalgamating various Harmonized System (HS) codes when capturing natural resource trade flows in UN Comtrade by selecting over 1350 HS codes and grouping them by resource type, thereby enabling global resource trade to be tracked at different stages of the value chain. It also reconciles exporters’ and importers’ reports into a single record, with each representing the aggregate value (US$) and weight (kg) of the given commodity flow from one country to another over a year. Data gaps and errors are well identified and managed in this dataset^6^.

## Results

**Figure 1:**
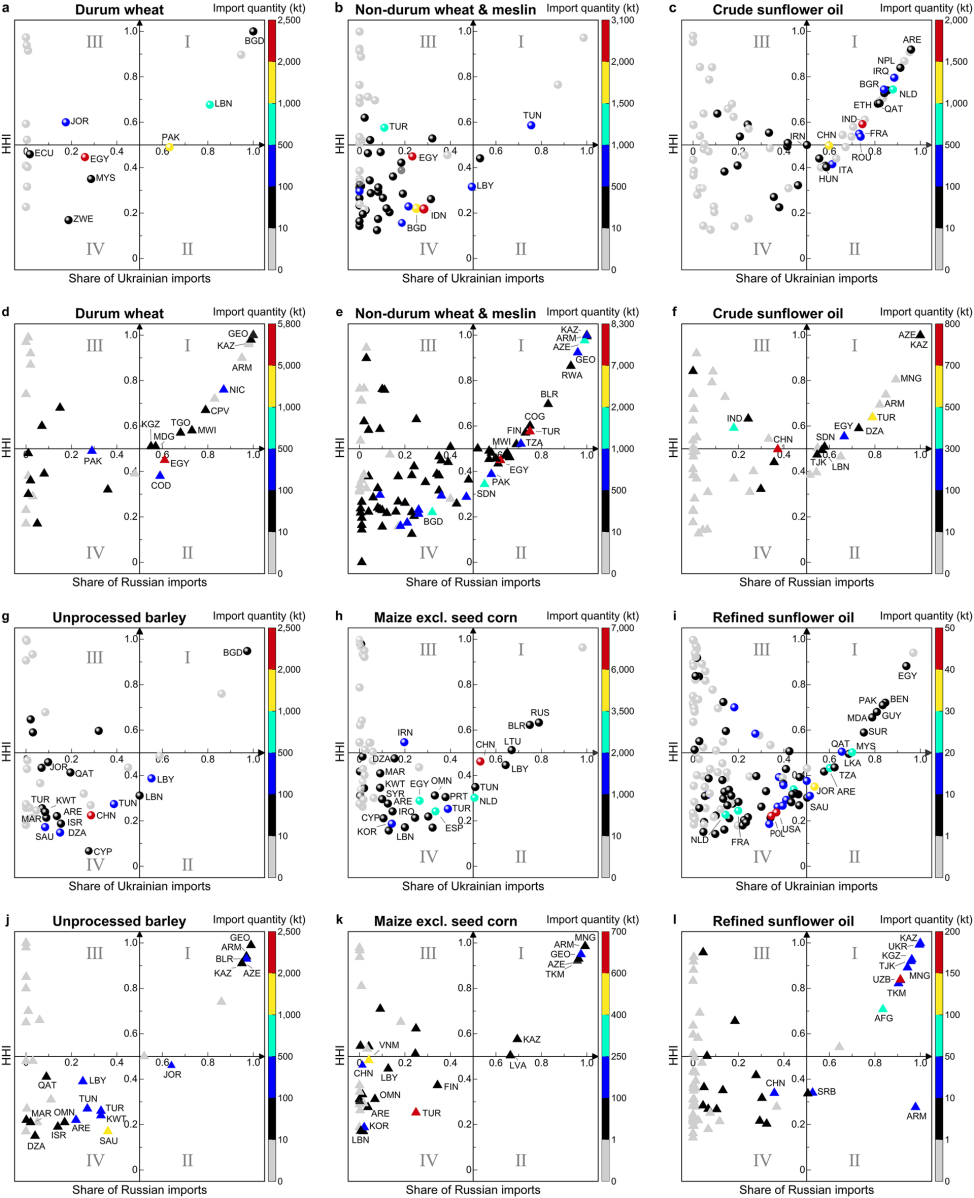
Dependency of trading partners on food imports from Russia and Ukraine in 2020. The y-axis shows the concentration of each commodity in trading partners’ markets by the Herfindahl–Hirschman Index (HHI), and the x-axis shows each trading partner’s import share of each commodity from Ukraine (**a**, **b**, **c**, **g**, **h**, **i**) and Russia (**d**, **e**, **f**, **j**, **k**, **l**). The vertical color bar shows the import quantity (kt). Three-letter codes represent country names. The panels are divided into zones (I–IV) that represent very high (I), high (II), low (III), and very low (IV) dependence on food imports from Russia and Ukraine. For a more detailed description, see Fig. S3a–l and Tables S2–S3.

We identified 156 countries and territories that imported food commodities of interest from Ukraine and 123 countries and territories that imported from Russia (Fig. 1, Tables S2, S3), with Russia having greater export quantities, except in the case of maize (Fig. 1h,k) and crude sunflower oil (Fig. 1c,f). Among these 279 trading partners, 28 in zones I and II were highly dependent on Russian non-durum wheat and meslin (Fig. 1e), and 29 and 22 were highly dependent on Ukrainian crude and refined sunflower oils, respectively (Fig. 1c,i). Such a high dependency led to the exposure of these trading partners to greater vulnerability to supply disruptions, as compared with countries in zones III and IV of other food commodities. Moreover, 24 in zone I [where both the import share and the Herfindahl–Hirschman Index (HHI) were over 0.9 for a commodity; see Fig. 1] were most at risk of food supply disruptions owing to the heaviest dependency. In particular, Georgia (GEO), Armenia (ARM), Kazakhstan (KAZ), Azerbaijan (AZE), and Mongolia (MNG) were extremely reliant on four to seven food commodities imported primarily from Russia, despite the small import quantities. Other countries had large quantities of food imports from Russia and Ukraine, could be affected to varying degrees if they could not quickly find alternative suppliers, although they were relatively less reliant on Russia or Ukraine. For example, Egypt (EGY) imported 2455 kt and 5785 kt of durum wheat from Ukraine and Russia (Fig. 1a,d), 3075 kt and 8255 kt of non-durum wheat from Ukraine and Russia (Fig. 1b,e), and 2545 kt of maize from Ukraine (Fig. 1h); China (CHN) and Saudi Arabia (SAU) each imported about 2400 kt of barley (Fig. 1g,j).

It is important to note that countries such as Syria, Iraq, and Eritrea did not have direct or large imports of certain food commodities (e.g., wheat) from Russia or Ukraine (Fig. 1a,b,d,e), but they still faced indirect impacts of external food access by relying on large imports from Russia’s and Ukraine’s major trading partners, such as Egypt and Turkey^6^. Specifically, Turkey and Egypt were responsible for more than 90% of wheat or meslin flour and sunflower oil (crude or refined) exports to Syria, Iraq, and Eritrea in 2020 (Table S4)^6^. However, Turkey and Egypt together reduced their agricultural and food exports by over US$4 billion in pursuit of domestic food security during 2022^20^. Given that over 52% of the calorie intake in Iraq and Syria comes from wheat, corn, and sunflower oil products (Fig. S2)^31^, coupled with the negative consequences of the war on food security in Sub-Saharan Africa^5,9,67^, Iraq^32^, and Syria^33^, the short-term external food accessibility of these three countries has been notably affected.

Further investigation revealed that the main geographic destinations for Russian and Ukrainian barley exports were the Middle East and North African (MENA) countries, whereas maize was primarily shipped to the European Union, followed by the MENA countries (Fig. 1g,h,j,k). These countries primarily utilize barley and maize as animal feed (Tables S5, S6)^31^. Shortages of animal feed grains might increase feed prices, animal slaughter, and the removal of small livestock producers from the supply chain. It may also cause income losses and limit the affordability and accessibility of animal-sourced foods. Such cascading effects have been evident in countries such as Iran^34^.

**Figure 2:**
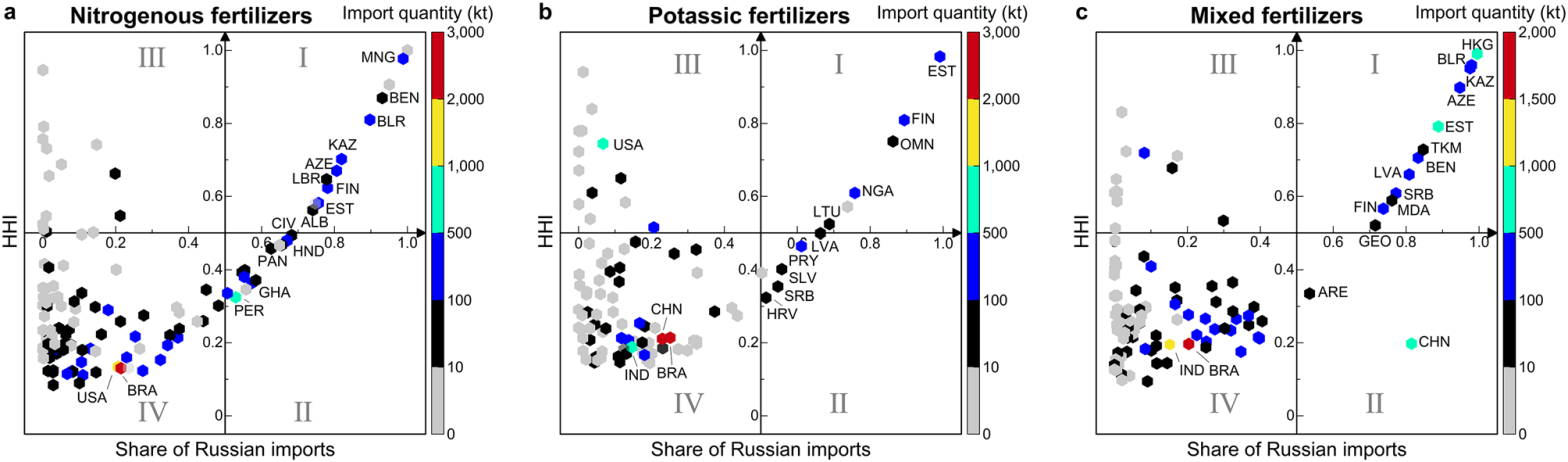
Dependency on Russian fertilizer imports in 2020. The y-axis shows the concentration of each commodity in trading partners’ markets by the Herfindahl–Hirschman Index (HHI), and the x-axis shows each trading partner’s share of total imports of fertilizers from Russia. The vertical color bar shows the import quantity (kt). Three-letter codes represent the country name. The panels are divided into zones (I–IV) that represent very high (I), high (II), low (III), and very low (IV) dependence on fertilizer imports from Russia. For more details, see Fig. S4a–c and Tables S7.

For fertilizers, if a country importing multiple fertilizers was counted only once, a total of 136 countries and territories imported Russian fertilizers in 2020 (Fig. 2, Table S7). Of these trading partners, 25 (18%) imported 736 kt of one type of fertilizer, 24 (18%) imported 999 kt of both mixed fertilizers and nitrogenous or potassic fertilizers, and 87 (64%) imported approximately 32,982 kt of all three types of fertilizers (Table S7). An extremely high reliance on Russia (with both import shares and HHI > 0.9 for the corresponding commodity) was observed in Estonia (EST), Niger (NGA), and Kyrgyzstan (KGZ) for potassic fertilizers; in Mongolia, Central African Republic (CAF), and Dominica (DMA) for nitrogenous fertilizers; and in Hong Kong SAR of China (HKG), Belarus (BLR), and Kazakhstan for mixed fertilizers. In addition, Brazil (BRA) was the largest importer (7332 kt total imports) of all fertilizers, followed by the United States (2959 kt), China (2725 kt), India (2330 kt), and Estonia (1522 kt), in spite of their very low dependency (Fig. 2, Table S7). Given that fertilizers cannot be easily sourced elsewhere and their production cutbacks are directly related to rising energy prices, a reduced supply in the world market, or higher prices, or both, would be expected as the result of the war. A lack of access to adequate fertilizers could directly and negatively impact food production in future planting seasons for various crops in heavily import-dependent countries, thereby further disrupting food provision in vulnerable countries^37,38^. If crop yields fall because of fertilizer supply disruptions, particularly in some of the world’s largest agricultural producing countries (China, India, the United States, and Brazil, as ranked by 2021 gross production values^31^), then global food supplies may face more severe challenges in the coming years.

**Figure 3:**
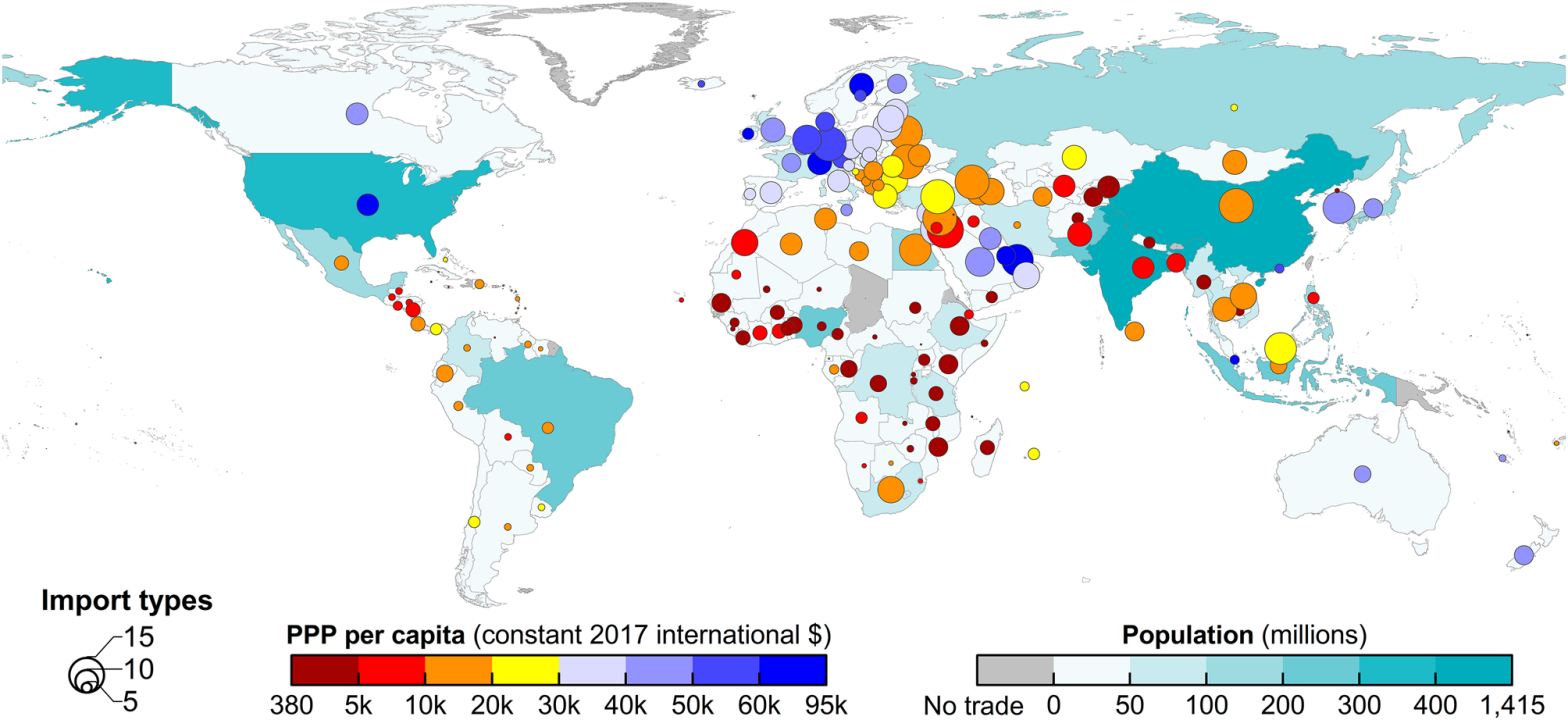
Comparison of import types of commodities, purchasing power parity (PPP) per capita, and population among trading partners of Russia and Ukraine in 2020. The number of import types varying from 1 to 15 includes twelve food commodities exported from Russia and Ukraine and three types of fertilizers exported from Russia. The currency unit of PPP per capita is constant 2017 international dollars (int$). For more details see Table S8.

Figure 3 illustrates the extent to which supply chain disruptions caused by the war are affecting external access to food and fertilizers in 176 countries and territories. A larger number of import types, a larger population, and a lower PPP per capita all contribute to the exposure of a country to greater vulnerability to supply disruptions, as compared with a country with a fewer import types, smaller population, and higher PPP per capita. Compared with Europe and Gulf Cooperation Council member states having, Sub-Saharan Africa and South Asia were much more vulnerable to disruptions of food and fertilizer supplies from Russia and Ukraine in the short term, primarily because of their low PPPs per capita and large populations. For example, Pakistan had 10 import varieties, a per capita PPP of int$5256 and a population of 218 million, and the Democratic Republic of the Congo and Ethiopia had seven or eight import varieties each, a population of around 100 million each, and per capita PPPs under int$2,700. Egypt, which had 13 import varieties, a population of 101 million, and a per capita PPP of int$12,801, is another country worthy of attention. Compared with countries under similar challenges accessing external food and fertilizers during the war, Egypt is unlikely to be self-sufficient in the twenty-first century under sustainable intensification of agriculture^39^, nor do its natural boundaries allow it to produce the 11 major food and fodder crops they will need, even if agricultural productivity increases by an average yield of 1.6% per year^36^. If Egypt’s economic development does not enable it to increase the affordability of high-priced food from other countries, these factors, coupled with the country’s low per capita PPP, likely will expose it to greater risk of short-term external food supply shocks compared with countries with per capita PPPs of more than int$30,000 and smaller populations.

## Discussion

It is clear that a sizable portion of global food supplies depends on food commodities and fertilizers imported from Russia and Ukraine via highly integrated global supply chains. Conflicts in some parts of the world can spill over into the global food supply chain, resulting in short-term external risks of varying degrees to food supplies beyond the point of origin. In response to external food supply shocks, multiple solutions are required. First, resource diversification and self-sufficiency should be promoted, but that requires careful consideration of what populations actually consume. For example, a rice-based diet in India, Bangladesh, or Vietnam can lower the importance of wheat in daily calorie intake^31^. In Nigeria, however, poor Nigerians would benefit little from wheat self-sufficiency approaches because wheat constitutes only 4% of their total food consumption and 8% of their starchy staple consumption. Conversely, millet, rice, cassava, and tubers are 10 times more important to their diet^9^.

Finding relevant substitutes is another effective solution, but it may not be an option for every country. The United States, Canada, France, Australia, Argentina, Germany, Kazakhstan, Poland, Romania, Lithuania, and Bulgaria, as top wheat exporters^31^, can become dominant alternatives to Russia and Ukraine. To offset the 18 Mt lost from Ukrainian wheat exports without any contribution from Russia, these eleven countries need to fill the gap by increasing wheat yields or expanding wheat cropping areas by at least 8% and applying an additional 548 kt of nitrogen fertilizer in aggregate, in the mid- to long-term^40^. This type of increase in wheat yield is not likely in the short or medium term^35,40^. In addition, considering uncertainties such as annual variability in yield, climate-change-induced crop failures, trade restrictions, soaring energy and fertilizer prices, reduced fertilizer use, and destruction of agricultural lands and infrastructure in Ukraine, additional declines in wheat exports are likely in the future^12,20,40,41^.

Furthermore, approximately 90% of Russia’s grain exports were shipped through the Black Sea in 2020^42^. Importing wheat from geographically distant alternative countries can lead to changes in maritime shipping routes and thus increased shipping costs in a period of rising fuel prices. For example, MENA countries may frequently ship grain cargo along routes between the east coast of the Americas and the Suez Canal rather than through the Bosphorus Strait and the Suez Canal, whereas many Asian countries use the shipping routes between Asia and the west coast of the Americas^43^. Countries with weak purchasing power and large number of types of agricultural imports (Fig. 3) may not be able to afford increased shipping costs. This has been reflected in Lebanon (per capita PPP: int$ 10,786 and 14 commodity imports from Russia and Ukraine in 2020), which attempted to import from Turkey, Egypt, Algeria, Morocco, and eastern European countries, rather than from western European countries and the United States^44^. In addition, countries such as Pakistan^45^ and Sri Lanka^46^ have run out of foreign currencies for imports because of severe financial crises.

However, some measures are commonly applicable to the stabilization of food supplies. In the short term, putting in place the necessary measures to free up exports of food commodities and fertilizers from Russia and Ukraine and distribute them from places of surplus to places of need can immediately relieve the pressure of such food shortages. In this respect, international organizations play a vital role. Under the United Nations’s moderating influence, the Black Sea Grain Initiative and a memorandum of understanding between the United Nations and the Russian Federation were set up to reintroduce food and fertilizer exports from Ukraine and Russia to global markets^47^. As of 5 March 2023, about 23 Mt of grain was exported under the Initiative, with developing countries such as Bangladesh, Yemen, Ethiopia, and Djibouti benefitting the most^47^.

In the long term, import-dependent countries need to identify and address the fundamental issues of food insecurity, such as grain financialization, which is the most important factor increasing global food insecurity and turmoil in developing countries that rely heavily on grain imports^48^. Meanwhile, the approach of growing the bulk of staple cereals in monocultures on an industrial scale needs to be reconsidered^38^. Diversifying cultivation to include more crop species where possible, rather than relying on a small number of food staples, would enhance food system resilience against crises involving conflicts and other unpredictable risks^49^. Research shows that countries with an effective crop diversity of four species at the national level are exposed to severe harvest failure every 7 years on average, whereas a diversity of 10 reduces the occurrence of failures to once in 60 years^50^.

Additionally, new directions in research into the world’s food supply need to be embraced in the wake of the war, including the development of research into diversification and decentralization strategies for food access. The analysis and visualization of potential supply-disruption risks of key agricultural commodities and inputs (e.g., fertilizer, animal feed) through multi-regional supply chains associated with global trade are critical for such strategy formulation. Knowing the potential impact of supply disruptions on an import-dependent country’s final domestic demand via supply chains per unit of commodity and input due to possible crises in each producing/exporting country will allow importers to optimize domestic production structures, manage inventories, and mitigate the risks of over-reliance on a single market and supply channel.

We close this paper by mentioning major limitations of this study and the relaxation of which would provide directions for future research. First, the simple setting of the global food supply chain did not allow for cross-sectional and cross-country evaluation of ripple effects of supply shocks, nor were we able to explicitly track agricultural products at every stage of the food supply chain. Additionally, the insufficient consideration of food insecurity determinants did not allow for the impacts on each country’s food security to be comprehensibly assessed and compared. An effective approach to delving into the interaction of a multitude of driving forces affecting food security on both the demand and supply sides is multi-regional input–output modelling^51–53^; such modelling should account for drivers that affect both national and international food security. It is hoped that these contribute to our better understanding of complexity of food security issues.

### Supplementary Information

Supplementary Information.

## Data Availability

The data used to select target commodities exported from Ukraine and Russia were based on the Atlas of Economic Complexity Dataverse by the Growth Lab at Harvard University, publicly available at https://atlas.cid.harvard.edu/explore. The food supply and utilization accounts provided in Tables S2 and S3 were obtained from FAOSTAT of the Food and Agriculture Organization of the United Nations^31^, available at https://www.fao.org/faostat/en/#data. The data used to support the study’s findings, as shown in Figs. 1 and 2, are publicly available from the Chatham House Resource Trade Database (CHRTD)^6^ at https://resourcetrade.earth/. The data for PPP per capita and population for different countries (see Fig. 3) were sourced from the World Economic Outlook Database of the International Monetary Fund (IMF)^65^ (available at https://www.imf.org/en/Publications/WEO/weo-database/2023/April) and the Real GDP (purchasing power parity) of the Central Intelligence Agency^66^ (available at https://www.cia.gov/the-world-factbook/field/real-gdp-purchasing-power-parity) for eight countries when the data were insufficient in the IMF datasets. All data generated during this study are in the Supplementary Information.
