# Supplementary material for: Countries’ vulnerability to food supply disruptions caused by the Russia–Ukraine war from a trade dependency perspective
Source: Sci Rep. 2023 Oct 3;13:16591. doi: 10.1038/s41598-023-43883-4 (PMC10547748; doi:10.1038/s41598-023-43883-4)
Supplement: Supplementary file 1 — Supplementary Information. [file 41598_2023_43883_MOESM1_ESM.docx]

**Supplementary Information for**

Countries’ vulnerability to food supply disruptions caused by the Russia-Ukraine war from a trade dependency perspective

Zhengyang Zhang^1, *^, Meshal J. Abdullah^2^, Guochang Xu^3^, Kazuyo Matsubae^1^, and Xianlai Zeng^3, *^

^1^ Graduate School of Environmental Studies, Tohoku University, Sendai, 980-0874, Japan.

^2^ Kuwait Environment Public Authority, Kuwait City, 12066, Kuwait.

^3^ School of Environment, Tsinghua University, Beijing, 100084, China.

^*^Corresponding author

E-mail: zhengyang.zhang.a8@tohoku.ac.jp, xlzeng@tsinghua.edu.cn

**This PDF file includes:**

Figures S1 to S4

Tables S1 to S8





Fig. S1 Global market shares of selected commodities from Ukraine and Russia in 2019.

Table S1. Global market shares of selected commodities from Ukraine and Russia in 2019

| **Commodity** | **Resource** | **HS Code** | **Russia** | | **Ukraine** | |
| --- | --- | --- | --- | --- | --- | --- |
|  |  |  | **Export weight (kt)** | **Trade share (%)** | **Export weight (kt)** | **Trade share (%)** |
| Durum wheat |  | 100110 | 2,449 | 20.6 | 3,991 | 9.2 |
| Non-durum wheat & meslin |  | 100190 | 31,021 | 15.6 | 20,405 | 9.4 |
| Unprocessed barley |  | 100300 | 3,521 | 9.9 | 3,664 | 10.0 |
| Maize excluding seed corn |  | 100590 | 3,100 | 1.9 | 30,676 | 15.6 |
| Crude sunflower oil |  | 151211 | 2,419 | 21.1 | 5,502 | 50.0 |
| Refined sunflower oil |  | 151219 | 536 | 12.9 | 519 | 12.1 |
| Nitrogenous fertilizers | Ammonium nitrate, including solution, in pack > 10 kg | 310230 | 3,705 |  | 0.1 |  |
|  | Ammonium nitrate limestone etc. mixes, pack > 10 kg | 310240 | 554 |  | 109 |  |
|  | Ammonium sulphate, in packs > 10 kg | 310221 | 332 |  | 85 |  |
|  | Ammonium sulphate-nitrate mix, double salts, pack> 10 kg | 310229 | 133 |  | - |  |
|  | Calcium-ammonium nitrate mix, double salts pack > 10 kg | 310260 | 44 |  | 8 |  |
|  | Nitrogenous fertilizers, mixes, nes, pack > 10 kg | 310290 | 133 |  | 0.2 |  |
|  | Sodium nitrate, in packs > 10 kg | 310250 | 15 |  | 0.1 |  |
|  | Urea-ammonium nitrate mixes in solution, pack > 10 kg | 310280 | 2,641 |  | 0.000003 |  |
|  | Urea, including aqueous solution in packs > 10 kg | 310210 | 6,778 |  | 701 |  |
| **Subtotal** | | **3102** | **14,334** | **13.2** | **905** | **0.9** |
| Potassic fertilizers | Potassic fertilizers, mixes, nes, pack > 10 kg | 310490 | 201 |  | 2 |  |
|  | Potassium chloride, in packs > 10 kg | 310420 | 9,385 |  | 58 |  |
|  | Potassium sulphate, in packs > 10 kg | 310430 | 60 |  | 0.1 |  |
| **Subtotal** | | **3104** | **9,645** | **15.7** | **60** | **0.1** |
| Mixed fertilizers | Diammonium phosphate, in packs > 10 kg | 310530 | 1,646 |  | - |  |
|  | Fertilizer mixes in tablets etc. or in packs < 10 kg | 310510 | 6 |  | 0.2 |  |
|  | Fertilizers containing phosphorus & potassium, <=10 kg | 310560 | 30 |  | 1.3 |  |
|  | Fertilizers, mixes, nes | 310590 | 218 |  | 1.4 |  |
|  | Fertilizers with nitrates & phosphates, nes, <=10 kg | 310551 | 641 |  | 0.1 |  |
|  | Fertilizers with nitrogen & phosphorus nes, <=10 kg | 310559 | 731 |  | 1.1 |  |
|  | Monoammonium phosphate & mix with diammonium, <=10 kg | 310540 | 2,283 |  | 0.2 |  |
|  | Nitrogen-phosphorus-potassium fertilizers, pack > 10 kg | 310520 | 5,655 |  | 0.8 |  |
|  | **Subtotal** | **3105** | **11,210** | **14.7** | **5.1** | **0.0002** |

Data source: The Growth Lab at Harvard University. (2019). “Growth Projections and Complexity Rankings, Harvard Dataverse V3”. https://doi.org/10.7910/dvn/xtaqmc

Table S2. Dependency of trading partners on food imports from Ukraine in 2020.

| **No.** | **Code** | **Importer** | **Resource** | **Import quantity (kt)** | **Import share** | **Herfindahl–Hirschman Index** | **Dependency on Ukraine** |
| --- | --- | --- | --- | --- | --- | --- | --- |
| 1 | AFG | Afghanistan | Crude sunflower oil | 0.02 | 0.004 | 0.38 | Very low |
| 1 | AFG | Afghanistan | Refined sunflower oil | 0.2 | 0.002 | 0.71 | Low |
| 2 | ALB | Albania | Non-durum wheat & meslin | 22 | 0.09 | 0.47 | Very low |
| 2 | ALB | Albania | Refined sunflower oil | 7 | 0.24 | 0.22 | Very low |
| 3 | DZA | Algeria | Crude sunflower oil | 13 | 0.24 | 0.59 | Low |
| 3 | DZA | Algeria | Durum wheat | 5 | 0.01 | 0.40 | Very low |
| 3 | DZA | Algeria | Maize excl. seed corn | 773 | 0.15 | 0.48 | Very low |
| 3 | DZA | Algeria | Unprocessed barley | 126 | 0.15 | 0.15 | Very low |
| 4 | AGO | Angola | Refined sunflower oil | 0.4 | 0.11 | 0.41 | Very low |
| 5 | ATG | Antigua and Barbuda | Refined sunflower oil | 0.0001 | 0.05 | 0.52 | Low |
| 6 | ARM | Armenia | Crude sunflower oil | 0.3 | 0.13 | 0.69 | Low |
| 6 | ARM | Armenia | Refined sunflower oil | 0.4 | 0.02 | 0.96 | Low |
| 7 | AUS | Australia | Crude sunflower oil | 5 | 0.16 | 0.57 | Low |
| 7 | AUS | Australia | Crude sunflower oil | 5 | 0.16 | 0.57 | Low |
| 7 | AUS | Australia | Refined sunflower oil | 18 | 0.38 | 0.39 | Very low |
| 8 | AUT | Austria | Crude sunflower oil | 4 | 0.13 | 0.18 | Very low |
| 8 | AUT | Austria | Non-durum wheat & meslin | 0.3 | 0.0003 | 0.35 | Very low |
| 8 | AUT | Austria | Refined sunflower oil | 3 | 0.05 | 0.20 | Very low |
| 9 | AZE | Azerbaijan | Refined sunflower oil | 0.2 | 0.01 | 0.83 | Low |
| 9 | AZE | Azerbaijan | Unprocessed barley | 0.02 | 0.00 | 0.91 | Low |
| 10 | BHS | Bahamas | Refined sunflower oil | 0.0001 | 0.01 | 0.69 | Low |
| 11 | BHR | Bahrain | Refined sunflower oil | 1 | 0.18 | 0.28 | Very low |
| 11 | BHR | Bahrain | Unprocessed barley | 0.4 | 0.15 | 0.28 | Very low |
| 12 | BGD | Bangladesh | Durum wheat | 10 | 1.00 | 1.00 | Very high |
| 12 | BGD | Bangladesh | Maize excl. seed corn | 113 | 0.002 | 0.42 | Very low |
| 12 | BGD | Bangladesh | Non-durum wheat & meslin | 1,505 | 0.25 | 0.22 | Very low |
| 12 | BGD | Bangladesh | Refined sunflower oil | 3 | 0.34 | 0.21 | Very low |
| 12 | BGD | Bangladesh | Unprocessed barley | 21 | 0.97 | 0.95 | Very high |
| 13 | BRB | Barbados | Refined sunflower oil | 0.1 | 0.05 | 0.82 | Low |
| 14 | BLR | Belarus | Crude sunflower oil | 1.2 | 0.08 | 0.84 | Low |
| 14 | BLR | Belarus | Maize excl. seed corn | 59 | 0.75 | 0.62 | Very high |
| 14 | BLR | Belarus | Non-durum wheat & meslin | 1 | 0.07 | 0.70 | Low |
| 14 | BLR | Belarus | Refined sunflower oil | 14 | 0.18 | 0.70 | Low |
| 14 | BLR | Belarus | Unprocessed barley | 4 | 0.03 | 0.93 | Low |

**Table S2.** Continued.

| **No.** | **Code** | **Importer** | **Resource** | **Import quantity (kt)** | **Import share** | **Herfindahl–Hirschman Index** | **Dependency on Ukraine** |
| --- | --- | --- | --- | --- | --- | --- | --- |
| 15 | BEL | Belgium | Crude sunflower oil | 1 | 0.002 | 0.59 | Low |
| 15 | BEL | Belgium | Maize excl. seed corn | 418 | 0.24 | 0.31 | Very low |
| 15 | BEL | Belgium | Refined sunflower oil | 18 | 0.06 | 0.31 | Very low |
| 16 | BLZ | Belize | Refined sunflower oil | 0.00005 | 0.0001 | 0.72 | Low |
| 17 | BEN | Benin | Durum wheat | 0.002 | 1.00 | 1.00 | Very high |
| 17 | BEN | Benin | Maize excl. seed corn | 0.03 | 0.02 | 0.40 | Very low |
| 17 | BEN | Benin | Refined sunflower oil | 1 | 0.85 | 0.72 | Very high |
| 17 | BEN | Benin | Unprocessed barley | 0.003 | 0.98 | 0.95 | Very high |
| 18 | BIH | Bosnia Herzegovina | Crude sunflower oil | 2 | 0.05 | 0.48 | Very low |
| 18 | BIH | Bosnia Herzegovina | Durum wheat | 0.00001 | 0.000004 | 0.41 | Very low |
| 19 | BWA | Botswana | Crude sunflower oil | 0.1 | 0.15 | 0.66 | Low |
| 19 | BWA | Botswana | Refined sunflower oil | 0.3 | 0.01 | 0.97 | Low |
| 20 | BRA | Brazil | Refined sunflower oil | 0.1 | 0.05 | 0.18 | Very low |
| 21 | VGB | British Virgin Islands | Refined sunflower oil | 0.0003 | 0.97 | 0.94 | Very high |
| 22 | BGR | Bulgaria | Crude sunflower oil | 25 | 0.86 | 0.74 | Very high |
| 22 | BGR | Bulgaria | Maize excl. seed corn | 0.2 | 0.01 | 0.26 | Very low |
| 22 | BGR | Bulgaria | Refined sunflower oil | 1 | 0.05 | 0.40 | Very low |
| 22 | BGR | Bulgaria | Unprocessed barley | 0.03 | 0.05 | 0.25 | Very low |
| 23 | BFA | Burkina Faso | Refined sunflower oil | 0.02 | 0.13 | 0.21 | Very low |
| 24 | BDI | Burundi | Refined sunflower oil | 0.1 | 0.11 | 0.70 | Low |
| 25 | KHM | Cambodia | Non-durum wheat & meslin | 0.4 | 0.01 | 0.89 | Low |
| 26 | CMR | Cameroon | Refined sunflower oil | 0.02 | 0.06 | 0.26 | Very low |
| 27 | CAN | Canada | Crude sunflower oil | 0.1 | 0.08 | 0.13 | Very low |
| 27 | CAN | Canada | Maize excl. seed corn | 13 | 0.01 | 0.98 | Low |
| 27 | CAN | Canada | Refined sunflower oil | 8 | 0.14 | 0.60 | Low |
| 28 | CYM | Cayman Islands | Refined sunflower oil | 0.0001 | 0.14 | 0.39 | Very low |
| 29 | CAF | Central African Republic | Refined sunflower oil | 0.02 | 0.09 | 0.78 | Low |
| 30 | CHL | Chile | Crude sunflower oil | 0.000003 | 0.0001 | 0.98 | Low |
| 30 | CHL | Chile | Refined sunflower oil | 0.1 | 0.001 | 0.90 | Low |
| 31 | CHN | China | Crude sunflower oil | 1,178 | 0.60 | 0.50 | High |
| 31 | CHN | China | Maize excl. seed corn | 6,957 | 0.53 | 0.46 | High |
| 31 | CHN | China | Non-durum wheat & meslin | 6 | 0.00003 | 0.24 | Very low |
| 31 | CHN | China | Refined sunflower oil | 23 | 0.44 | 0.34 | Very low |

**Table S2.** Continued.

| **No.** | **Code** | **Importer** | **Resource** | **Import quantity (kt)** | **Import share** | **Herfindahl–Hirschman Index** | **Dependency on Ukraine** |
| --- | --- | --- | --- | --- | --- | --- | --- |
| 31 | CHN | China | Unprocessed barley | 2,405 | 0.29 | 0.22 | Very low |
| 32 | HKG | China, Hong Kong SAR | Crude sunflower oil | 1 | 0.89 | 0.80 | Very high |
| 32 | HKG | China, Hong Kong SAR | Non-durum wheat & meslin | 1 | 0.000005 | 0.51 | Low |
| 32 | HKG | China, Hong Kong SAR | Refined sunflower oil | 0.1 | 0.05 | 0.15 | Very low |
| 33 | COM | Comoros | Refined sunflower oil | 0.01 | 0.07 | 0.43 | Very low |
| 34 | COD | Congo, Democratic Republic | Crude sunflower oil | 0.04 | 0.07 | 0.47 | Very low |
| 34 | COD | Congo, Democratic Republic | Refined sunflower oil | 0.4 | 0.13 | 0.20 | Very low |
| 35 | COG | Congo, Republic | Refined sunflower oil | 0.1 | 0.20 | 0.18 | Very low |
| 36 | COK | Cook Islands | Refined sunflower oil | 0.00003 | 0.01 | 0.50 | Low |
| 37 | CRI | Costa Rica | Crude sunflower oil | 4 | 0.76 | 0.61 | Very high |
| 37 | CRI | Costa Rica | Maize excl. seed corn | 1 | 0.001 | 0.79 | Low |
| 37 | CRI | Costa Rica | Refined sunflower oil | 2 | 0.47 | 0.39 | Very low |
| 38 | CIV | Cote d'Ivoire | Refined sunflower oil | 4 | 0.58 | 0.42 | High |
| 39 | HRV | Croatia | Refined sunflower oil | 0.1 | 0.003 | 0.35 | Very low |
| 40 | CUB | Cuba | Refined sunflower oil | 1 | 0.34 | 0.53 | Low |
| 41 | CYP | Cyprus | Crude sunflower oil | 1 | 0.05 | 0.64 | Low |
| 41 | CYP | Cyprus | Maize excl. seed corn | 35 | 0.11 | 0.21 | Very low |
| 41 | CYP | Cyprus | Non-durum wheat & meslin | 9 | 0.18 | 0.16 | Very low |
| 41 | CYP | Cyprus | Refined sunflower oil | 2 | 0.10 | 0.22 | Very low |
| 41 | CYP | Cyprus | Unprocessed barley | 43 | 0.28 | 0.07 | Very low |
| 42 | CZE | Czech Republic | Crude sunflower oil | 2 | 0.09 | 0.23 | Very low |
| 42 | CZE | Czech Republic | Maize excl. seed corn | 4 | 0.02 | 0.51 | Low |
| 42 | CZE | Czech Republic | Non-durum wheat & meslin | 0.1 | 0.001 | 0.68 | Low |
| 42 | CZE | Czech Republic | Refined sunflower oil | 7 | 0.13 | 0.38 | Very low |
| 43 | DNK | Denmark | Non-durum wheat & meslin | 0.0002 | 0.000002 | 0.33 | Very low |
| 43 | DNK | Denmark | Refined sunflower oil | 0.1 | 0.01 | 0.28 | Very low |
| 44 | DJI | Djibouti | Crude sunflower oil | 10 | 0.93 | 0.87 | Very high |
| 44 | DJI | Djibouti | Non-durum wheat & meslin | 17 | 0.32 | 0.53 | Low |
| 44 | DJI | Djibouti | Refined sunflower oil | 3 | 0.02 | 0.92 | Low |
| 45 | DMA | Dominica | Refined sunflower oil | 0.1 | 0.39 | 0.39 | Very low |
| 46 | DOM | Dominican Republic | Crude sunflower oil | 0.5 | 0.17 | 0.68 | Low |

**Table S2.** Continued.

| **No.** | **Code** | **Importer** | **Resource** | **Import quantity (kt)** | **Import share** | **Herfindahl–Hirschman Index** | **Dependency on Ukraine** |
| --- | --- | --- | --- | --- | --- | --- | --- |
| 47 | ECU | Ecuador | Crude sunflower oil | 0.01 | 0.0003 | 0.95 | Low |
| 47 | ECU | Ecuador | Durum wheat | 13 | 0.02 | 0.46 | Very low |
| 47 | ECU | Ecuador | Non-durum wheat & meslin | 23 | 0.08 | 0.31 | Very low |
| 47 | ECU | Ecuador | Refined sunflower oil | 0.03 | 0.01 | 0.70 | Low |
| 48 | EGY | Egypt | Crude sunflower oil | 52 | 0.33 | 0.55 | Low |
| 48 | EGY | Egypt | Durum wheat | 2,455 | 0.26 | 0.45 | Very low |
| 48 | EGY | Egypt | Maize excl. seed corn | 2,545 | 0.26 | 0.29 | Very low |
| 48 | EGY | Egypt | Non-durum wheat & meslin | 3,075 | 0.23 | 0.45 | Very low |
| 48 | EGY | Egypt | Refined sunflower oil | 5 | 0.94 | 0.88 | Very high |
| 48 | EGY | Egypt | Unprocessed barley | 6 | 0.86 | 0.76 | Very high |
| 49 | SLV | El Salvador | Refined sunflower oil | 1 | 0.30 | 0.26 | Very low |
| 50 | EST | Estonia | Crude sunflower oil | 2 | 0.34 | 0.49 | Very low |
| 50 | EST | Estonia | Maize excl. seed corn | 1 | 0.02 | 0.29 | Very low |
| 50 | EST | Estonia | Non-durum wheat & meslin | 6 | 0.39 | 0.46 | Very low |
| 50 | EST | Estonia | Refined sunflower oil | 3 | 0.46 | 0.32 | Very low |
| 51 | SWZ | Eswatini | Non-durum wheat & meslin | 2 | 0.0004 | 0.39 | Very low |
| 52 | ETH | Ethiopia | Crude sunflower oil | 11 | 0.81 | 0.68 | Very high |
| 52 | ETH | Ethiopia | Durum wheat | 3 | 0.01 | 0.92 | Low |
| 52 | ETH | Ethiopia | Non-durum wheat & meslin | 178 | 0.19 | 0.29 | Very low |
| 52 | ETH | Ethiopia | Refined sunflower oil | 2 | 0.01 | 0.51 | Low |
| 53 | FJI | Fiji | Refined sunflower oil | 0.1 | 0.06 | 0.40 | Very low |
| 54 | FIN | Finland | Maize excl. seed corn | 0.3 | 0.01 | 0.37 | Very low |
| 55 | FRA | France | Crude sunflower oil | 132 | 0.73 | 0.55 | Very high |
| 55 | FRA | France | Maize excl. seed corn | 0.002 | 0.000004 | 0.28 | Very low |
| 55 | FRA | France | Refined sunflower oil | 23 | 0.20 | 0.25 | Very low |
| 56 | PYF | French Polynesia | Maize excl. seed corn | 0.0001 | 0.00002 | 1.00 | Low |
| 57 | GAB | Gabon | Refined sunflower oil | 2 | 0.03 | 0.55 | Low |
| 58 | GMB | Gambia | Refined sunflower oil | 0.3 | 0.17 | 0.28 | Very low |
| 59 | GEO | Georgia | Crude sunflower oil | 1 | 0.29 | 0.58 | Low |
| 59 | GEO | Georgia | Maize excl. seed corn | 3 | 0.02 | 0.95 | Low |
| 59 | GEO | Georgia | Non-durum wheat & meslin | 0.05 | 0.0001 | 0.92 | Low |
| 59 | GEO | Georgia | Refined sunflower oil | 1 | 0.03 | 0.84 | Low |
| 59 | GEO | Georgia | Unprocessed barley | 0.001 | 0.00 | 0.99 | Low |

**Table S2.** Continued.

| **No.** | **Code** | **Importer** | **Resource** | **Import quantity (kt)** | **Import share** | **Herfindahl–Hirschman Index** | **Dependency on Ukraine** |
| --- | --- | --- | --- | --- | --- | --- | --- |
| 60 | DEU | Germany | Crude sunflower oil | 57 | 0.14 | 0.38 | Very low |
| 60 | DEU | Germany | Durum wheat | 0.00001 | 0.00000002 | 0.23 | Very low |
| 60 | DEU | Germany | Maize excl. seed corn | 383 | 0.003 | 0.31 | Very low |
| 60 | DEU | Germany | Non-durum wheat & meslin | 10 | 0.00000001 | 0.31 | Very low |
| 60 | DEU | Germany | Refined sunflower oil | 1 | 0.01 | 0.22 | Very low |
| 60 | DEU | Germany | Unprocessed barley | 0.00 | 0.00 | 0.20 | Very low |
| 61 | GHA | Ghana | Refined sunflower oil | 11 | 0.41 | 0.29 | Very low |
| 62 | GRC | Greece | Crude sunflower oil | 13 | 0.20 | 0.41 | Very low |
| 62 | GRC | Greece | Non-durum wheat & meslin | 87 | 0.10 | 0.27 | Very low |
| 62 | GRC | Greece | Refined sunflower oil | 0.2 | 0.01 | 0.68 | Low |
| 62 | GRC | Greece | Unprocessed barley | 4 | 0.06 | 0.37 | Very low |
| 63 | GRD | Grenada | Refined sunflower oil | 0.03 | 0.29 | 0.58 | Low |
| 64 | GIN | Guinea | Refined sunflower oil | 1 | 0.38 | 0.21 | Very low |
| 65 | GUY | Guyana | Refined sunflower oil | 2 | 0.81 | 0.68 | Very high |
| 65 | GUY | Guyana | Unprocessed barley | 0.03 | 0.45 | 0.44 | Very low |
| 66 | HUN | Hungary | Crude sunflower oil | 21 | 0.58 | 0.41 | High |
| 66 | HUN | Hungary | Maize excl. seed corn | 26 | 0.24 | 0.21 | Very low |
| 66 | HUN | Hungary | Refined sunflower oil | 1 | 0.05 | 0.31 | Very low |
| 67 | ISL | Iceland | Crude sunflower oil | 0.0002 | 0.001 | 0.43 | Very low |
| 67 | ISL | Iceland | Refined sunflower oil | 0.002 | 0.004 | 0.18 | Very low |
| 68 | IND | India | Crude sunflower oil | 1,891 | 0.75 | 0.59 | Very high |
| 68 | IND | India | Maize excl. seed corn | 2 | 0.02 | 0.43 | Very low |
| 68 | IND | India | Refined sunflower oil | 0.3 | 0.05 | 0.35 | Very low |
| 68 | IND | India | Unprocessed barley | 7 | 0.09 | 0.68 | Low |
| 69 | IDN | Indonesia | Non-durum wheat & meslin | 2,884 | 0.28 | 0.22 | Very low |
| 69 | IDN | Indonesia | Refined sunflower oil | 1 | 0.09 | 0.22 | Very low |
| 70 | IRN | Iran | Crude sunflower oil | 30 | 0.50 | 0.50 | High |
| 70 | IRN | Iran | Maize excl. seed corn | 1,221 | 0.20 | 0.55 | Low |
| 70 | IRN | Iran | Unprocessed barley | 17 | 0.02 | 0.65 | Low |
| 71 | IRQ | Iraq | Crude sunflower oil | 455 | 0.88 | 0.80 | Very high |
| 71 | IRQ | Iraq | Maize excl. seed corn | 110 | 0.15 | 0.24 | Very low |
| 71 | IRQ | Iraq | Refined sunflower oil | 0.1 | 0.002 | 0.88 | Low |

**Table S2.** Continued.

| **No.** | **Code** | **Importer** | **Resource** | **Import quantity (kt)** | **Import share** | **Herfindahl–Hirschman Index** | **Dependency on Ukraine** |
| --- | --- | --- | --- | --- | --- | --- | --- |
| 72 | IRL | Ireland | Maize excl. seed corn | 452 | 0.30 | 0.22 | Very low |
| 72 | IRL | Ireland | Refined sunflower oil | 0.02 | 0.002 | 0.22 | Very low |
| 73 | ISR | Israel | Crude sunflower oil | 0.02 | 0.05 | 0.14 | Very low |
| 73 | ISR | Israel | Durum wheat | 0.1 | 0.004 | 0.52 | Low |
| 73 | ISR | Israel | Maize excl. seed corn | 371 | 0.15 | 0.19 | Very low |
| 73 | ISR | Israel | Non-durum wheat & meslin | 123 | 0.0001 | 0.23 | Very low |
| 73 | ISR | Israel | Refined sunflower oil | 15 | 0.34 | 0.19 | Very low |
| 73 | ISR | Israel | Unprocessed barley | 77 | 0.15 | 0.19 | Very low |
| 74 | ITA | Italy | Crude sunflower oil | 354 | 0.61 | 0.42 | High |
| 74 | ITA | Italy | Maize excl. seed corn | 718 | 0.13 | 0.16 | Very low |
| 74 | ITA | Italy | Non-durum wheat & meslin | 228 | 0.0000001 | 0.14 | Very low |
| 74 | ITA | Italy | Refined sunflower oil | 2 | 0.05 | 0.15 | Very low |
| 75 | JPN | Japan | Crude sunflower oil | 5 | 0.18 | 0.14 | Very low |
| 75 | JPN | Japan | Maize excl. seed corn | 0.05 | 0.000003 | 0.55 | Low |
| 75 | JPN | Japan | Refined sunflower oil | 0.1 | 0.01 | 0.21 | Very low |
| 76 | JOR | Jordan | Crude sunflower oil | 0.2 | 0.05 | 0.50 | Low |
| 76 | JOR | Jordan | Durum wheat | 134 | 0.18 | 0.60 | Low |
| 76 | JOR | Jordan | Maize excl. seed corn | 35 | 0.05 | 0.54 | Low |
| 76 | JOR | Jordan | Non-durum wheat & meslin | 224 | 0.18 | 0.42 | Very low |
| 76 | JOR | Jordan | Refined sunflower oil | 33 | 0.53 | 0.35 | High |
| 76 | JOR | Jordan | Unprocessed barley | 58 | 0.10 | 0.46 | Very low |
| 77 | KAZ | Kazakhstan | Non-durum wheat & meslin | 0.1 | 0.0002 | 1.00 | Low |
| 78 | KEN | Kenya | Crude sunflower oil | 5 | 0.84 | 0.71 | Very high |
| 78 | KEN | Kenya | Maize excl. seed corn | 9 | 0.05 | 0.54 | Low |
| 78 | KEN | Kenya | Non-durum wheat & meslin | 46 | 0.02 | 0.21 | Very low |
| 78 | KEN | Kenya | Refined sunflower oil | 0.2 | 0.15 | 0.27 | Very low |
| 79 | KOR | Korea, Republic | Crude sunflower oil | 0.02 | 0.02 | 0.28 | Very low |
| 79 | KOR | Korea, Republic | Durum wheat | 0.00001 | 0.000001 | 0.97 | Low |
| 79 | KOR | Korea, Republic | Maize excl. seed corn | 1,638 | 0.14 | 0.19 | Very low |
| 79 | KOR | Korea, Republic | Non-durum wheat & meslin | 413 | 0.000000004 | 0.27 | Very low |
| 79 | KOR | Korea, Republic | Refined sunflower oil | 15 | 0.39 | 0.27 | Very low |
| 80 | KWT | Kuwait | Crude sunflower oil | 2 | 0.95 | 0.91 | Very high |
| 80 | KWT | Kuwait | Maize excl. seed corn | 13 | 0.09 | 0.35 | Very low |

**Table S2.** Continued.

| **No.** | **Code** | **Importer** | **Resource** | **Import quantity (kt)** | **Import share** | **Herfindahl–Hirschman Index** | **Dependency on Ukraine** |
| --- | --- | --- | --- | --- | --- | --- | --- |
| 80 | KWT | Kuwait | Non-durum wheat & meslin | 0.2 | 0.000001 | 0.76 | Low |
| 80 | KWT | Kuwait | Refined sunflower oil | 2 | 0.16 | 0.21 | Very low |
| 80 | KWT | Kuwait | Unprocessed barley | 43 | 0.08 | 0.24 | Very low |
| 81 | LAO | Lao PDR | Non-durum wheat & meslin | 3 | 0.99 | 0.97 | Very high |
| 82 | LVA | Latvia | Crude sunflower oil | 0.1 | 0.03 | 0.31 | Very low |
| 82 | LVA | Latvia | Maize excl. seed corn | 5 | 0.06 | 0.50 | Low |
| 82 | LVA | Latvia | Refined sunflower oil | 3 | 0.22 | 0.20 | Very low |
| 83 | LBN | Lebanon | Crude sunflower oil | 35 | 0.41 | 0.49 | Very low |
| 83 | LBN | Lebanon | Durum wheat | 512 | 0.81 | 0.68 | Very high |
| 83 | LBN | Lebanon | Maize excl. seed corn | 151 | 0.20 | 0.17 | Very low |
| 83 | LBN | Lebanon | Non-durum wheat & meslin | 251 | 0.31 | 0.26 | Very low |
| 83 | LBN | Lebanon | Refined sunflower oil | 16 | 0.50 | 0.38 | High |
| 83 | LBN | Lebanon | Unprocessed barley | 28 | 0.50 | 0.31 | High |
| 84 | LBR | Liberia | Maize excl. seed corn | 0.4 | 0.26 | 0.44 | Very low |
| 84 | LBR | Liberia | Refined sunflower oil | 0.1 | 0.25 | 0.40 | Very low |
| 85 | LBY | Libya | Maize excl. seed corn | 495 | 0.64 | 0.45 | High |
| 85 | LBY | Libya | Non-durum wheat & meslin | 546 | 0.49 | 0.32 | Very low |
| 85 | LBY | Libya | Refined sunflower oil | 19 | 0.27 | 0.58 | Low |
| 85 | LBY | Libya | Unprocessed barley | 439 | 0.55 | 0.39 | High |
| 86 | LTU | Lithuania | Crude sunflower oil | 9 | 0.42 | 0.31 | Very low |
| 86 | LTU | Lithuania | Maize excl. seed corn | 200 | 0.67 | 0.51 | Very high |
| 86 | LTU | Lithuania | Refined sunflower oil | 8 | 0.47 | 0.27 | Very low |
| 87 | MDG | Madagascar | Maize excl. seed corn | 1 | 0.98 | 0.96 | Very high |
| 87 | MDG | Madagascar | Refined sunflower oil | 1 | 0.14 | 0.22 | Very low |
| 88 | MWI | Malawi | Non-durum wheat & meslin | 1 | 0.01 | 0.52 | Low |
| 89 | MYS | Malaysia | Crude sunflower oil | 44 | 0.46 | 0.32 | Very low |
| 89 | MYS | Malaysia | Durum wheat | 51 | 0.29 | 0.35 | Very low |
| 89 | MYS | Malaysia | Maize excl. seed corn | 1 | 0.0004 | 0.54 | Low |
| 89 | MYS | Malaysia | Non-durum wheat & meslin | 297 | 0.24 | 0.22 | Very low |
| 89 | MYS | Malaysia | Refined sunflower oil | 27 | 0.70 | 0.50 | Very high |
| 89 | MYS | Malaysia | Unprocessed barley | 1.4 | 0.28 | 0.27 | Very low |
| 90 | MDV | Maldives | Refined sunflower oil | 0.1 | 0.02 | 0.40 | Very low |
| 91 | MLI | Mali | Refined sunflower oil | 0.02 | 0.07 | 0.32 | Very low |

**Table S2.** Continued.

| **No.** | **Code** | **Importer** | **Resource** | **Import quantity (kt)** | **Import share** | **Herfindahl–Hirschman Index** | **Dependency on Ukraine** |
| --- | --- | --- | --- | --- | --- | --- | --- |
| 92 | MLT | Malta | Crude sunflower oil | 0.2 | 0.67 | 0.49 | High |
| 92 | MLT | Malta | Refined sunflower oil | 1 | 0.50 | 0.30 | High |
| 92 | MLT | Malta | Unprocessed barley | 0.00 | 0.00 | 0.27 | Very low |
| 93 | MHL | Marshall Islands | Refined sunflower oil | 0.001 | 0.05 | 0.86 | Low |
| 94 | MRT | Mauritania | Non-durum wheat & meslin | 88 | 0.13 | 0.20 | Very low |
| 94 | MRT | Mauritania | Refined sunflower oil | 1 | 0.50 | 0.35 | High |
| 95 | MUS | Mauritius | Crude sunflower oil | 0.2 | 0.03 | 0.84 | Low |
| 95 | MUS | Mauritius | Refined sunflower oil | 1 | 0.45 | 0.32 | Very low |
| 96 | MEX | Mexico | Crude sunflower oil | 3 | 0.23 | 0.46 | Very low |
| 96 | MEX | Mexico | Non-durum wheat & meslin | 33 | 0.02 | 0.62 | Low |
| 97 | MDA | Moldova | Crude sunflower oil | 0.01 | 0.89 | 0.79 | Very high |
| 97 | MDA | Moldova | Durum wheat | 1 | 0.95 | 0.90 | Very high |
| 97 | MDA | Moldova | Maize excl. seed corn | 3 | 0.10 | 0.41 | Very low |
| 97 | MDA | Moldova | Non-durum wheat & meslin | 3 | 0.87 | 0.77 | Very high |
| 97 | MDA | Moldova | Refined sunflower oil | 5 | 0.79 | 0.66 | Very high |
| 97 | MDA | Moldova | Unprocessed barley | 1 | 0.79 | 0.65 | Very high |
| 98 | MNG | Mongolia | Refined sunflower oil | 1 | 0.04 | 0.89 | Low |
| 99 | MNE | Montenegro | Refined sunflower oil | 0.1 | 0.01 | 0.73 | Low |
| 100 | MAR | Morocco | Crude sunflower oil | 13 | 0.21 | 0.54 | Low |
| 100 | MAR | Morocco | Maize excl. seed corn | 268 | 0.09 | 0.41 | Very low |
| 100 | MAR | Morocco | Non-durum wheat & meslin | 942 | 0.22 | 0.23 | Very low |
| 100 | MAR | Morocco | Refined sunflower oil | 0.004 | 0.001 | 0.64 | Low |
| 100 | MAR | Morocco | Unprocessed barley | 100 | 0.09 | 0.21 | Very low |
| 101 | MOZ | Mozambique | Crude sunflower oil | 1 | 0.03 | 0.54 | Low |
| 101 | MOZ | Mozambique | Non-durum wheat & meslin | 68 | 0.08 | 0.12 | Very low |
| 101 | MOZ | Mozambique | Refined sunflower oil | 1 | 0.42 | 0.51 | Low |
| 102 | MMR | Myanmar | Non-durum wheat & meslin | 68 | 0.15 | 0.36 | Very low |
| 102 | MMR | Myanmar | Refined sunflower oil | 16 | 0.37 | 0.26 | Very low |
| 103 | NPL | Nepal | Crude sunflower oil | 26 | 0.91 | 0.84 | Very high |
| 103 | NPL | Nepal | Maize excl. seed corn | 8 | 0.02 | 0.93 | Low |
| 103 | NPL | Nepal | Refined sunflower oil | 1 | 0.52 | 0.36 | High |
| 104 | NLD | Netherlands | Crude sunflower oil | 651 | 0.86 | 0.74 | Very high |
| 104 | NLD | Netherlands | Maize excl. seed corn | 3,009 | 0.51 | 0.30 | High |

**Table S2.** Continued.

| **No.** | **Code** | **Importer** | **Resource** | **Import quantity (kt)** | **Import share** | **Herfindahl–Hirschman Index** | **Dependency on Ukraine** |
| --- | --- | --- | --- | --- | --- | --- | --- |
| 104 | NLD | Netherlands | Non-durum wheat & meslin | 27 | 0.01 | 0.32 | Very low |
| 104 | NLD | Netherlands | Refined sunflower oil | 21 | 0.14 | 0.23 | Very low |
| 104 | NLD | Netherlands | Unprocessed barley | 2 | 0.00 | 0.26 | Very low |
| 105 | NCL | New Caledonia | Non-durum wheat & meslin | 1 | 0.04 | 0.71 | Low |
| 105 | NCL | New Caledonia | Refined sunflower oil | 0.3 | 0.12 | 0.25 | Very low |
| 106 | NZL | New Zealand | Crude sunflower oil | 2 | 0.18 | 0.62 | Low |
| 106 | NZL | New Zealand | Maize excl. seed corn | 0.4 | 0.003 | 0.62 | Low |
| 106 | NZL | New Zealand | Non-durum wheat & meslin | 0.4 | 0.001 | 0.71 | Low |
| 106 | NZL | New Zealand | Refined sunflower oil | 5 | 0.43 | 0.28 | Very low |
| 107 | NER | Niger | Refined sunflower oil | 0.1 | 0.79 | 0.64 | Very high |
| 108 | NGA | Nigeria | Refined sunflower oil | 0.1 | 0.10 | 0.51 | Low |
| 109 | NOR | Norway | Crude sunflower oil | 0.1 | 0.07 | 0.79 | Low |
| 109 | NOR | Norway | Maize excl. seed corn | 5 | 0.06 | 0.46 | Very low |
| 109 | NOR | Norway | Non-durum wheat & meslin | 0.3 | 0.001 | 0.24 | Very low |
| 109 | NOR | Norway | Refined sunflower oil | 1 | 0.10 | 0.14 | Very low |
| 110 | OMN | Oman | Crude sunflower oil | 18 | 0.84 | 0.73 | Very high |
| 110 | OMN | Oman | Maize excl. seed corn | 126 | 0.33 | 0.31 | Very low |
| 110 | OMN | Oman | Non-durum wheat & meslin | 24 | 0.04 | 0.46 | Very low |
| 110 | OMN | Oman | Refined sunflower oil | 10 | 0.40 | 0.36 | Very low |
| 110 | OMN | Oman | Unprocessed barley | 4 | 0.03 | 0.21 | Very low |
| 111 | PAK | Pakistan | Durum wheat | 1,078 | 0.63 | 0.49 | High |
| 111 | PAK | Pakistan | Maize excl. seed corn | 1 | 0.06 | 0.30 | Very low |
| 111 | PAK | Pakistan | Non-durum wheat & meslin | 216 | 0.18 | 0.39 | Very low |
| 111 | PAK | Pakistan | Refined sunflower oil | 2 | 0.83 | 0.71 | Very high |
| 111 | PAK | Pakistan | Unprocessed barley | 1 | 0.25 | 0.32 | Very low |
| 112 | PAN | Panama | Refined sunflower oil | 0.2 | 0.13 | 0.45 | Very low |
| 113 | PER | Peru | Refined sunflower oil | 3 | 0.44 | 0.32 | Very low |
| 114 | PHL | Philippines | Durum wheat | 0.2 | 0.003 | 0.94 | Low |
| 114 | PHL | Philippines | Non-durum wheat & meslin | 634 | 0.00002 | 0.30 | Very low |
| 114 | PHL | Philippines | Refined sunflower oil | 3 | 0.22 | 0.18 | Very low |
| 115 | POL | Poland | Crude sunflower oil | 111 | 0.72 | 0.54 | Very high |
| 115 | POL | Poland | Durum wheat | 2 | 0.01 | 0.32 | Very low |
| 115 | POL | Poland | Maize excl. seed corn | 1 | 0.004 | 0.31 | Very low |

**Table S2.** Continued.

| **No.** | **Code** | **Importer** | **Resource** | **Import quantity (kt)** | **Import share** | **Herfindahl–Hirschman Index** | **Dependency on Ukraine** |
| --- | --- | --- | --- | --- | --- | --- | --- |
| 115 | POL | Poland | Non-durum wheat & meslin | 2 | 0.004 | 0.34 | Very low |
| 115 | POL | Poland | Refined sunflower oil | 45 | 0.34 | 0.22 | Very low |
| 115 | POL | Poland | Unprocessed barley | 0.02 | 0.00 | 0.18 | Very low |
| 116 | PRT | Portugal | Crude sunflower oil | 18 | 0.36 | 0.27 | Very low |
| 116 | PRT | Portugal | Maize excl. seed corn | 733 | 0.38 | 0.30 | Very low |
| 117 | QAT | Qatar | Crude sunflower oil | 11 | 0.82 | 0.68 | Very high |
| 117 | QAT | Qatar | Maize excl. seed corn | 4 | 0.04 | 0.54 | Low |
| 117 | QAT | Qatar | Non-durum wheat & meslin | 38 | 0.27 | 0.22 | Very low |
| 117 | QAT | Qatar | Refined sunflower oil | 12 | 0.65 | 0.50 | Very high |
| 117 | QAT | Qatar | Unprocessed barley | 55 | 0.20 | 0.41 | Very low |
| 118 | ROU | Romania | Crude sunflower oil | 5 | 0.23 | 0.27 | Very low |
| 118 | ROU | Romania | Refined sunflower oil | 8 | 0.23 | 0.19 | Very low |
| 118 | ROU | Romania | Unprocessed barley | 24 | 0.03 | 0.59 | Low |
| 119 | RUS | Russian Federation | Maize excl. seed corn | 16 | 0.79 | 0.63 | Very high |
| 119 | RUS | Russian Federation | Refined sunflower oil | 0.0002 | 0.0001 | 0.23 | Very low |
| 119 | RUS | Russian Federation | Unprocessed barley | 40 | 0.32 | 0.60 | Low |
| 120 | RWA | Rwanda | Refined sunflower oil | 1 | 0.15 | 0.41 | Very low |
| 121 | KNA | Saint Kitts and Nevis | Refined sunflower oil | 0.00004 | 0.003 | 0.99 | Low |
| 122 | VCT | Saint Vincent and the Grenadines | Refined sunflower oil | 0.00004 | 0.0003 | 1.00 | Low |
| 123 | SAU | Saudi Arabia | Crude sunflower oil | 55 | 0.5553 | 0.44 | High |
| 123 | SAU | Saudi Arabia | Maize excl. seed corn | 72 | 0.02 | 0.34 | Very low |
| 123 | SAU | Saudi Arabia | Non-durum wheat & meslin | 64 | 0.0001 | 0.33 | Very low |
| 123 | SAU | Saudi Arabia | Refined sunflower oil | 15 | 0.51 | 0.31 | High |
| 123 | SAU | Saudi Arabia | Unprocessed barley | 439 | 0.08 | 0.17 | Very low |
| 124 | SEN | Senegal | Crude sunflower oil | 2 | 0.56 | 0.40 | High |
| 124 | SEN | Senegal | Refined sunflower oil | 7 | 0.32 | 0.33 | Very low |
| 125 | SRB | Serbia | Durum wheat | 0.000002 | 0.00001 | 0.59 | Low |
| 125 | SRB | Serbia | Non-durum wheat & meslin | 0.02 | 0.00001 | 0.53 | Low |
| 126 | SYC | Seychelles | Maize excl. seed corn | 0.5 | 0.13 | 0.47 | Very low |
| 126 | SYC | Seychelles | Refined sunflower oil | 0.1 | 0.03 | 0.47 | Very low |
| 127 | SLE | Sierra Leone | Refined sunflower oil | 0.1 | 0.50 | 0.43 | High |
| 128 | SGP | Singapore | Crude sunflower oil | 3 | 0.63 | 0.44 | High |

**Table S2.** Continued.

| **No.** | **Code** | **Importer** | **Resource** | **Import quantity (kt)** | **Import share** | **Herfindahl–Hirschman Index** | **Dependency on Ukraine** |
| --- | --- | --- | --- | --- | --- | --- | --- |
| 128 | SGP | Singapore | Refined sunflower oil | 5 | 0.20 | 0.28 | Very low |
| 129 | SVK | Slovakia | Crude sunflower oil | 1 | 0.04 | 0.31 | Very low |
| 129 | SVK | Slovakia | Refined sunflower oil | 5 | 0.14 | 0.33 | Very low |
| 129 | SVK | Slovakia | Unprocessed barley | 0.3 | 0.00 | 0.43 | Very low |
| 130 | SOM | Somalia | Non-durum wheat & meslin | 40 | 0.53 | 0.44 | High |
| 130 | SOM | Somalia | Refined sunflower oil | 1 | 0.02 | 0.73 | Low |
| 131 | ZAF | South Africa | Crude sunflower oil | 0.4 | 0.002 | 0.37 | Very low |
| 131 | ZAF | South Africa | Maize excl. seed corn | 0.5 | 0.01 | 0.97 | Low |
| 131 | ZAF | South Africa | Non-durum wheat & meslin | 60 | 0.03 | 0.21 | Very low |
| 131 | ZAF | South Africa | Refined sunflower oil | 2 | 0.07 | 0.39 | Very low |
| 131 | ZAF | South Africa | Unprocessed barley | 0.1 | 0.00 | 0.99 | Low |
| 132 | SSD | South Sudan | Refined sunflower oil | 0.1 | 0.13 | 0.55 | Low |
| 133 | ESP | Spain | Crude sunflower oil | 433 | 0.73 | 0.54 | Very high |
| 133 | ESP | Spain | Maize excl. seed corn | 2,700 | 0.33 | 0.24 | Very low |
| 133 | ESP | Spain | Non-durum wheat & meslin | 373 | 0.0000001 | 0.16 | Very low |
| 133 | ESP | Spain | Refined sunflower oil | 4 | 0.15 | 0.27 | Very low |
| 133 | ESP | Spain | Unprocessed barley | 28 | 0.07 | 0.43 | Very low |
| 134 | LKA | Sri Lanka | Maize excl. seed corn | 6 | 0.26 | 0.23 | Very low |
| 134 | LKA | Sri Lanka | Non-durum wheat & meslin | 114 | 0.08 | 0.29 | Very low |
| 134 | LKA | Sri Lanka | Refined sunflower oil | 2 | 0.69 | 0.50 | High |
| 134 | LKA | Sri Lanka | Unprocessed barley | 2 | 0.59 | 0.49 | High |
| 135 | PSE | State of Palestine | Maize excl. seed corn | 0.6 | 0.02 | 0.92 | Low |
| 135 | PSE | State of Palestine | Refined sunflower oil | 8 | 0.40 | 0.36 | Very low |
| 136 | SDN | Sudan | Crude sunflower oil | 53 | 0.42 | 0.51 | Low |
| 136 | SDN | Sudan | Non-durum wheat & meslin | 110 | 0.05 | 0.34 | Very low |
| 136 | SDN | Sudan | Refined sunflower oil | 0.1 | 0.01 | 0.92 | Low |
| 137 | SUR | Suriname | Refined sunflower oil | 4 | 0.75 | 0.59 | Very high |
| 138 | SWE | Sweden | Refined sunflower oil | 2 | 0.13 | 0.30 | Very low |
| 139 | CHE | Switzerland | Crude sunflower oil | 3 | 0.09 | 0.20 | Very low |
| 139 | CHE | Switzerland | Maize excl. seed corn | 1 | 0.01 | 0.34 | Very low |
| 139 | CHE | Switzerland | Non-durum wheat & meslin | 10 | 0.02 | 0.22 | Very low |
| 139 | CHE | Switzerland | Refined sunflower oil | 3 | 0.14 | 0.16 | Very low |
| 139 | CHE | Switzerland | Unprocessed barley | 0.3 | 0.01 | 0.47 | Very low |

**Table S2.** Continued.

| **No.** | **Code** | **Importer** | **Resource** | **Import quantity (kt)** | **Import share** | **Herfindahl–Hirschman Index** | **Dependency on Ukraine** |
| --- | --- | --- | --- | --- | --- | --- | --- |
| 140 | SYR | Syria | Maize excl. seed corn | 22 | 0.10 | 0.30 | Very low |
| 141 | TZA | Tanzania | Non-durum wheat & meslin | 47 | 0.05 | 0.52 | Low |
| 141 | TZA | Tanzania | Refined sunflower oil | 8 | 0.62 | 0.44 | High |
| 142 | THA | Thailand | Crude sunflower oil | 8 | 0.92 | 0.85 | Very high |
| 142 | THA | Thailand | Maize excl. seed corn | 10 | 0.01 | 0.64 | Low |
| 142 | THA | Thailand | Non-durum wheat & meslin | 569 | 0.19 | 0.16 | Very low |
| 142 | THA | Thailand | Refined sunflower oil | 5 | 0.17 | 0.31 | Very low |
| 142 | THA | Thailand | Unprocessed barley | 0.2 | 0.00 | 1.00 | Low |
| 143 | TGO | Togo | Refined sunflower oil | 2 | 0.41 | 0.43 | Very low |
| 144 | TUN | Tunisia | Crude sunflower oil | 8 | 0.66 | 0.54 | Very high |
| 144 | TUN | Tunisia | Maize excl. seed corn | 508 | 0.51 | 0.35 | High |
| 144 | TUN | Tunisia | Non-durum wheat & meslin | 984 | 0.76 | 0.59 | Very high |
| 144 | TUN | Tunisia | Unprocessed barley | 371 | 0.39 | 0.27 | Very low |
| 144 | TUR | Turkey | Crude sunflower oil | 90.2 | 0.11 | 0.64 | Low |
| 144 | TUR | Turkey | Maize excl. seed corn | 1,025 | 0.39 | 0.25 | Very low |
| 144 | TUR | Turkey | Non-durum wheat & meslin | 1,025 | 0.11 | 0.58 | Low |
| 144 | TUR | Turkey | Refined sunflower oil | 0.3 | 0.01 | 0.94 | Low |
| 144 | TUR | Turkey | Unprocessed barley | 69 | 0.07 | 0.26 | Very low |
| 145 | TKM | Turkmenistan | Refined sunflower oil | 0.4 | 0.01 | 0.82 | Low |
| 146 | UGA | Uganda | Non-durum wheat & meslin | 72 | 0.12 | 0.22 | Very low |
| 147 | ARE | United Arab Emirates | Crude sunflower oil | 46 | 0.959 | 0.92 | Very high |
| 147 | ARE | United Arab Emirates | Maize excl. seed corn | 71 | 0.12 | 0.28 | Very low |
| 147 | ARE | United Arab Emirates | Non-durum wheat & meslin | 72 | 0.05 | 0.29 | Very low |
| 147 | ARE | United Arab Emirates | Refined sunflower oil | 28 | 0.60 | 0.43 | High |
| 147 | ARE | United Arab Emirates | Unprocessed barley | 63 | 0.14 | 0.22 | Very low |
| 148 | GBR | United Kingdom | Crude sunflower oil | 98 | 0.59 | 0.40 | High |
| 148 | GBR | United Kingdom | Maize excl. seed corn | 730 | 0.32 | 0.17 | Very low |
| 148 | GBR | United Kingdom | Non-durum wheat & meslin | 31 | 0.01 | 0.20 | Very low |
| 148 | GBR | United Kingdom | Refined sunflower oil | 5 | 0.03 | 0.26 | Very low |
| 148 | GBR | United Kingdom | Unprocessed barley | 2 | 0.02 | 0.26 | Very low |
| 149 | USA | United States | Crude sunflower oil | 32 | 0.38 | 0.23 | Very low |
| 149 | USA | United States | Non-durum wheat & meslin | 0.01 | 0.00001 | 0.92 | Low |
| 149 | USA | United States | Refined sunflower oil | 41 | 0.37 | 0.24 | Very low |

**Table S2.** Continued.

| **No.** | **Code** | **Importer** | **Resource** | **Import quantity (kt)** | **Import share** | **Herfindahl–Hirschman Index** | **Dependency on Ukraine** |
| --- | --- | --- | --- | --- | --- | --- | --- |
| 150 | UZB | Uzbekistan | Refined sunflower oil | 2 | 0.01 | 0.84 | Low |
| 151 | VUT | Vanuatu | Refined sunflower oil | 0.000004 | 0.0001 | 0.75 | Low |
| 152 | VEN | Venezuela | Refined sunflower oil | 1.8 | 0.28 | 0.40 | Very low |
| 153 | VNM | Vietnam | Maize excl. seed corn | 165 | 0.01 | 0.48 | Very low |
| 153 | VNM | Vietnam | Non-durum wheat & meslin | 251 | 0.08 | 0.17 | Very low |
| 153 | VNM | Vietnam | Refined sunflower oil | 3 | 0.16 | 0.34 | Very low |
| 154 | YEM | Yemen | Maize excl. seed corn | 0.1 | 0.0002 | 0.98 | Low |
| 155 | YEM | Yemen | Non-durum wheat & meslin | 708 | 0.23 | 0.23 | Very low |
| 155 | YEM | Yemen | Refined sunflower oil | 8 | 0.13 | 0.72 | Low |
| 156 | ZWE | Zimbabwe | Durum wheat | 36 | 0.19 | 0.17 | Very low |

Data on import quantity were obtained from Chatham House (2021), ‘resourcetrade.earth’, https://resourcetrade.earth/

Table S3. Dependency of trading partners on food imports from Russia in 2020.

| **No.** | **Code** | **Importer** | **Resource** | **Import quantity (kt)** | **Import share** | **Herfindahl–Hirschman Index** | **Dependency on Russia** |
| --- | --- | --- | --- | --- | --- | --- | --- |
| 1 | AFG | Afghanistan | Refined sunflower oil | 74 | 0.84 | 0.71 | Very high |
| 1 | AFG | Afghanistan | Non-durum wheat & meslin | 3 | 0.64 | 0.47 | High |
| 1 | AFG | Afghanistan | Crude sunflower oil | 3 | 0.51 | 0.38 | High |
| 2 | ALB | Albania | Non-durum wheat & meslin | 152 | 0.64 | 0.47 | High |
| 2 | ALB | Albania | Refined sunflower oil | 9 | 0.29 | 0.22 | Very low |
| 3 | DZA | Algeria | Crude sunflower oil | 40 | 0.73 | 0.59 | Very high |
| 3 | DZA | Algeria | Unprocessed barley | 31 | 0.04 | 0.15 | Vey low |
| 4 | AGO | Angola | Non-durum wheat & meslin | 200 | 0.34 | 0.39 | Very low |
| 5 | ARM | Armenia | Durum wheat | 0.4 | 0.95 | 0.90 | Very high |
| 5 | ARM | Armenia | Non-durum wheat & meslin | 343 | 1.00 | 0.99 | Very high |
| 5 | ARM | Armenia | Crude sunflower oil | 2 | 0.82 | 0.69 | Very high |
| 5 | ARM | Armenia | Unprocessed barley | 23 | 0.97 | 0.94 | Very high |
| 5 | ARM | Armenia | Maize except seed corn | 57 | 0.99 | 0.99 | Very high |
| 5 | ARM | Armenia | Refined sunflower oil | 25 | 0.98 | 0.96 | Very high |
| 6 | AUS | Australia | Crude sunflower oil | 1 | 0.74 | 0.57 | Very high |
| 6 | AUS | Australia | Refined sunflower oil | 1 | 0.01 | 0.39 | Very low |
| 7 | AUT | Austria | Crude sunflower oil | 0.0001 | 0.000004 | 0.18 | Very low |
| 7 | AUT | Austria | Refined sunflower oil | 0.0001 | 0.000002 | 0.20 | Very low |
| 8 | AZE | Azerbaijan | Durum wheat | 8 | 0.83 | 0.72 | Very high |
| 8 | AZE | Azerbaijan | Non-durum wheat & meslin | 1,357 | 0.99 | 0.98 | Very high |
| 8 | AZE | Azerbaijan | Crude sunflower oil | 40 | 1.00 | 1.00 | Very high |
| 8 | AZE | Azerbaijan | Unprocessed barley | 56 | 0.95 | 0.91 | Very high |
| 8 | AZE | Azerbaijan | Maize except seed corn | 46 | 0.96 | 0.93 | Very high |
| 8 | AZE | Azerbaijan | Refined sunflower oil | 11 | 0.91 | 0.83 | Very high |
| 9 | BHS | Bahamas | Refined sunflower oil | 0.0003 | 0.01 | 0.69 | Low |
| 10 | BHR | Bahrain | Refined sunflower oil | 0.02 | 0.004 | 0.28 | Very low |
| 11 | BGD | Bangladesh | Non-durum wheat & meslin | 1,941 | 0.32 | 0.22 | Very low |
| 11 | BGD | Bangladesh | Refined sunflower oil | 0.3 | 0.03 | 0.21 | Very low |
| 12 | BLR | Belarus | Durum wheat | 1 | 0.98 | 0.96 | Very high |
| 12 | BLR | Belarus | Non-durum wheat & meslin | 14 | 0.83 | 0.70 | Very high |
| 12 | BLR | Belarus | Unprocessed barley | 142 | 0.97 | 0.93 | Very high |
| 12 | BLR | Belarus | Refined sunflower oil | 64 | 0.82 | 0.70 | Very high |

**Table S3.** Continued.

| **No.** | **Code** | **Importer** | **Resource** | **Import quantity (kt)** | **Import share** | **Herfindahl–Hirschman Index** | **Dependency on Russia** |
| --- | --- | --- | --- | --- | --- | --- | --- |
| 12 | BLR | Belarus | Crude sunflower oil | 13 | 0.0001 | 0.84 | Low |
| 12 | BLR | Belarus | Maize except seed corn | 19 | 0.25 | 0.62 | Low |
| 13 | BEL | Belgium | Unprocessed barley | 0.00001 | 0.00000001 | 0.80 | Low |
| 14 | BEN | Benin | Non-durum wheat & meslin | 28 | 1.00 | 1.00 | Very high |
| 15 | BRA | Brazil | Non-durum wheat & meslin | 238 | 0.04 | 0.58 | Low |
| 16 | BGR | Bulgaria | Crude sunflower oil | 0.003 | 0.0001 | 0.74 | Low |
| 16 | BGR | Bulgaria | Non-durum wheat & meslin | 9 | 0.31 | 0.24 | Very low |
| 16 | BGR | Bulgaria | Maize except seed corn | 0.04 | 0.002 | 0.26 | Very low |
| 16 | BGR | Bulgaria | Refined sunflower oil | 0.003 | 0.0002 | 0.40 | Very low |
| 17 | BFA | Burkina Faso | Durum wheat | 3 | 0.98 | 0.97 | Very high |
| 17 | BFA | Burkina Faso | Non-durum wheat & meslin | 4 | 0.03 | 0.94 | Low |
| 18 | BDI | Burundi | Non-durum wheat & meslin | 20 | 0.23 | 0.22 | Very low |
| 19 | CPV | Cabo Verde | Durum wheat | 15 | 0.79 | 0.67 | Very high |
| 19 | CPV | Cabo Verde | Non-durum wheat & meslin | 10 | 0.65 | 0.48 | High |
| 20 | KHM | Cambodia | Refined sunflower oil | 0.05 | 0.02 | 0.58 | Low |
| 20 | KHM | Cambodia | Durum wheat | 0.5 | 0.03 | 0.29 | Very low |
| 21 | CMR | Cameroon | Non-durum wheat & meslin | 389 | 0.48 | 0.36 | Very low |
| 22 | CAN | Canada | Maize except seed corn | 0.001 | 0.000001 | 0.98 | Low |
| 22 | CAN | Canada | Refined sunflower oil | 0.02 | 0.0004 | 0.60 | Low |
| 22 | CAN | Canada | Crude sunflower oil | 0.03 | 0.02 | 0.13 | Very low |
| 23 | CHN | China | Durum wheat | 21 | 0.01 | 0.48 | Very low |
| 23 | CHN | China | Non-durum wheat & meslin | 34 | 0.005 | 0.24 | Very low |
| 23 | CHN | China | Crude sunflower oil | 734 | 0.37 | 0.50 | Very low |
| 23 | CHN | China | Maize except seed corn | 169 | 0.01 | 0.46 | Very low |
| 23 | CHN | China | Refined sunflower oil | 19 | 0.36 | 0.34 | Very low |
| 23 | CHN | China | Unprocessed barley | 13 | 0.002 | 0.22 | Vey low |
| 24 | COD | Congo, Democratic Republic | Durum wheat | 212 | 0.59 | 0.38 | High |
| 24 | COD | Congo, Democratic Republic | Non-durum wheat & meslin | 199 | 0.61 | 0.43 | High |
| 24 | COD | Congo, Democratic Republic | Maize except seed corn | 6 | 0.09 | 0.71 | Low |

**Table S3.** Continued.

| **No.** | **Code** | **Importer** | **Resource** | **Import quantity (kt)** | **Import share** | **Herfindahl–Hirschman Index** | **Dependency on Russia** |
| --- | --- | --- | --- | --- | --- | --- | --- |
| 25 | COG | Congo, Republic | Durum wheat | 6 | 1.00 | 1.00 | Very high |
| 25 | COG | Congo, Republic | Non-durum wheat & meslin | 148 | 0.75 | 0.60 | Very high |
| 25 | COG | Congo, Republic | Refined sunflower oil | 0.04 | 0.07 | 0.18 | Very low |
| 26 | CIV | Cote d'Ivoire | Non-durum wheat & meslin | 65 | 0.10 | 0.76 | Low |
| 26 | CIV | Cote d'Ivoire | Refined sunflower oil | 2 | 0.28 | 0.42 | Very low |
| 27 | CYP | Cyprus | Crude sunflower oil | 9 | 0.79 | 0.64 | Very high |
| 27 | CYP | Cyprus | Non-durum wheat & meslin | 8 | 0.17 | 0.16 | Very low |
| 27 | CYP | Cyprus | Maize except seed corn | 7 | 0.02 | 0.21 | Very low |
| 28 | CZE | Czech Republic | Crude sunflower oil | 0.0001 | 0.00001 | 0.23 | Very low |
| 29 | DNK | Denmark | Non-durum wheat & meslin | 3 | 0.03 | 0.33 | Very low |
| 29 | DNK | Denmark | Crude sunflower oil | 1 | 0.18 | 0.37 | Very low |
| 29 | DNK | Denmark | Maize except seed corn | 3 | 0.01 | 0.33 | Very low |
| 29 | DNK | Denmark | Refined sunflower oil | 0.1 | 0.01 | 0.28 | Very low |
| 30 | DJI | Djibouti | Refined sunflower oil | 0.3 | 0.002 | 0.92 | Low |
| 31 | EGY | Egypt | Crude sunflower oil | 103 | 0.66 | 0.55 | Very high |
| 31 | EGY | Egypt | Durum wheat | 5,785 | 0.61 | 0.45 | High |
| 31 | EGY | Egypt | Non-durum wheat & meslin | 8,255 | 0.62 | 0.45 | High |
| 31 | EGY | Egypt | Maize except seed corn | 1 | 0.0001 | 0.29 | Very low |
| 32 | EST | Estonia | Crude sunflower oil | 0.002 | 0.0003 | 0.49 | Very low |
| 32 | EST | Estonia | Maize except seed corn | 1 | 0.02 | 0.29 | Very low |
| 32 | EST | Estonia | Refined sunflower oil | 2 | 0.30 | 0.32 | Very low |
| 33 | SWZ | Eswatini | Non-durum wheat & meslin | 1 | 0.02 | 0.39 | Very low |
| 34 | ETH | Ethiopia | Durum wheat | 7 | 0.02 | 0.92 | Low |
| 34 | ETH | Ethiopia | Refined sunflower oil | 0.1 | 0.0004 | 0.51 | Low |
| 34 | ETH | Ethiopia | Non-durum wheat & meslin | 53 | 0.06 | 0.29 | Very low |
| 34 | ETH | Ethiopia | Unprocessed barley | 0.3 | 0.03 | 0.48 | Vey low |
| 35 | FIN | Finland | Non-durum wheat & meslin | 88 | 0.73 | 0.57 | Very high |
| 35 | FIN | Finland | Maize except seed corn | 8 | 0.34 | 0.37 | Very low |
| 35 | FIN | Finland | Refined sunflower oil | 0.0001 | 0.0001 | 0.36 | Very low |
| 35 | FIN | Finland | Unprocessed barley | 0.000002 | 0.00002 | 0.17 | Vey low |
| 36 | FRA | France | Crude sunflower oil | 3 | 0.02 | 0.55 | Low |
| 36 | FRA | France | Durum wheat | 0.0001 | 0.000001 | 0.17 | Very low |

**Table S3.** Continued.

| **No.** | **Code** | **Importer** | **Resource** | **Import quantity (kt)** | **Import share** | **Herfindahl–Hirschman Index** | **Dependency on Russia** |
| --- | --- | --- | --- | --- | --- | --- | --- |
| 37 | GAB | Gabon | Non-durum wheat & meslin | 13 | 0.11 | 0.76 | Low |
| 38 | GMB | Gambia | Non-durum wheat & meslin | 53 | 0.60 | 0.46 | High |
| 38 | GMB | Gambia | Refined sunflower oil | 0.03 | 0.01 | 0.28 | Very low |
| 39 | GEO | Georgia | Durum wheat | 3 | 1.00 | 1.00 | Very high |
| 39 | GEO | Georgia | Non-durum wheat & meslin | 516 | 0.96 | 0.92 | Very high |
| 39 | GEO | Georgia | Crude sunflower oil | 2 | 0.70 | 0.58 | Very high |
| 39 | GEO | Georgia | Unprocessed barley | 28 | 0.99 | 0.99 | Very high |
| 39 | GEO | Georgia | Maize except seed corn | 143 | 0.97 | 0.95 | Very high |
| 39 | GEO | Georgia | Refined sunflower oil | 41 | 0.91 | 0.84 | Very high |
| 40 | DEU | Germany | Durum wheat | 0.0001 | 0.0000001 | 0.23 | Very low |
| 40 | DEU | Germany | Non-durum wheat & meslin | 0.3 | 0.0001 | 0.31 | Very low |
| 40 | DEU | Germany | Crude sunflower oil | 0.4 | 0.001 | 0.38 | Very low |
| 40 | DEU | Germany | Maize except seed corn | 17 | 0.0001 | 0.31 | Very low |
| 40 | DEU | Germany | Refined sunflower oil | 0.2 | 0.001 | 0.22 | Very low |
| 41 | GHA | Ghana | Non-durum wheat & meslin | 233 | 0.29 | 0.35 | Very low |
| 41 | GHA | Ghana | Refined sunflower oil | 0.1 | 0.003 | 0.29 | Very low |
| 42 | GRC | Greece | Non-durum wheat & meslin | 145 | 0.17 | 0.27 | Very low |
| 42 | GRC | Greece | Crude sunflower oil | 0.00004 | 0.000001 | 0.41 | Very low |
| 42 | GRC | Greece | Maize except seed corn | 15 | 0.02 | 0.40 | Very low |
| 43 | GIN | Guinea | Non-durum wheat & meslin | 60 | 0.17 | 0.38 | Very low |
| 43 | GIN | Guinea | Refined sunflower oil | 0.2 | 0.12 | 0.21 | Very low |
| 44 | HUN | Hungary | Refined sunflower oil | 0.00003 | 0.000002 | 0.31 | Very low |
| 45 | ISL | Iceland | Unprocessed barley | 0.00002 | 0.000001 | 0.48 | Vey low |
| 37 | GAB | Gabon | Non-durum wheat & meslin | 13 | 0.11 | 0.76 | Low |
| 38 | GMB | Gambia | Non-durum wheat & meslin | 53 | 0.60 | 0.46 | High |
| 38 | GMB | Gambia | Refined sunflower oil | 0.03 | 0.01 | 0.28 | Very low |
| 39 | GEO | Georgia | Durum wheat | 3 | 1.00 | 1.00 | Very high |
| 39 | GEO | Georgia | Non-durum wheat & meslin | 516 | 0.96 | 0.92 | Very high |
| 39 | GEO | Georgia | Crude sunflower oil | 2 | 0.70 | 0.58 | Very high |
| 39 | GEO | Georgia | Unprocessed barley | 28 | 0.99 | 0.99 | Very high |
| 39 | GEO | Georgia | Maize except seed corn | 143 | 0.97 | 0.95 | Very high |
| 39 | GEO | Georgia | Refined sunflower oil | 41 | 0.91 | 0.84 | Very high |

**Table S3.** Continued.

| **No.** | **Code** | **Importer** | **Resource** | **Import quantity (kt)** | **Import share** | **Herfindahl–Hirschman Index** | **Dependency on Russia** |
| --- | --- | --- | --- | --- | --- | --- | --- |
| 40 | DEU | Germany | Durum wheat | 0.0001 | 0.0000001 | 0.23 | Very low |
| 40 | DEU | Germany | Non-durum wheat & meslin | 0.3 | 0.0001 | 0.31 | Very low |
| 40 | DEU | Germany | Crude sunflower oil | 0.4 | 0.001 | 0.38 | Very low |
| 40 | DEU | Germany | Maize except seed corn | 17 | 0.0001 | 0.31 | Very low |
| 40 | DEU | Germany | Refined sunflower oil | 0.2 | 0.001 | 0.22 | Very low |
| 41 | GHA | Ghana | Non-durum wheat & meslin | 233 | 0.29 | 0.35 | Very low |
| 41 | GHA | Ghana | Refined sunflower oil | 0.1 | 0.003 | 0.29 | Very low |
| 42 | GRC | Greece | Non-durum wheat & meslin | 145 | 0.17 | 0.27 | Very low |
| 42 | GRC | Greece | Crude sunflower oil | 0.00004 | 0.000001 | 0.41 | Very low |
| 42 | GRC | Greece | Maize except seed corn | 15 | 0.02 | 0.40 | Very low |
| 43 | GIN | Guinea | Non-durum wheat & meslin | 60 | 0.17 | 0.38 | Very low |
| 43 | GIN | Guinea | Refined sunflower oil | 0.2 | 0.12 | 0.21 | Very low |
| 44 | HUN | Hungary | Refined sunflower oil | 0.00003 | 0.000002 | 0.31 | Very low |
| 45 | ISL | Iceland | Unprocessed barley | 0.00002 | 0.000001 | 0.48 | Vey low |
| 46 | IND | India | Crude sunflower oil | 455 | 0.18 | 0.59 | Low |
| 46 | IND | India | Refined sunflower oil | 0.02 | 0.003 | 0.35 | Very low |
| 47 | IDN | Indonesia | Non-durum wheat & meslin | 48 | 0.005 | 0.22 | Very low |
| 47 | IDN | Indonesia | Refined sunflower oil | 0.02 | 0.003 | 0.22 | Very low |
| 48 | IRQ | Iraq | Non-durum wheat & meslin | 0.1 | 0.01 | 0.71 | Low |
| 48 | IRQ | Iraq | Refined sunflower oil | 0.1 | 0.002 | 0.88 | Low |
| 49 | ISR | Israel | Non-durum wheat & meslin | 201 | 0.09 | 0.23 | Very low |
| 49 | ISR | Israel | Crude sunflower oil | 0.03 | 0.06 | 0.14 | Very low |
| 49 | ISR | Israel | Maize except seed corn | 0.005 | 0.000002 | 0.19 | Very low |
| 49 | ISR | Israel | Refined sunflower oil | 7 | 0.15 | 0.19 | Very low |
| 49 | ISR | Israel | Unprocessed barley | 70 | 0.14 | 0.19 | Vey low |
| 40 | DEU | Germany | Durum wheat | 0.0001 | 0.0000001 | 0.23 | Very low |
| 40 | DEU | Germany | Non-durum wheat & meslin | 0.3 | 0.0001 | 0.31 | Very low |
| 40 | DEU | Germany | Crude sunflower oil | 0.4 | 0.001 | 0.38 | Very low |
| 40 | DEU | Germany | Maize except seed corn | 17 | 0.0001 | 0.31 | Very low |
| 40 | DEU | Germany | Refined sunflower oil | 0.2 | 0.001 | 0.22 | Very low |
| 41 | GHA | Ghana | Non-durum wheat & meslin | 233 | 0.29 | 0.35 | Very low |
| 41 | GHA | Ghana | Refined sunflower oil | 0.1 | 0.003 | 0.29 | Very low |

**Table S3.** Continued.

| **No.** | **Code** | **Importer** | **Resource** | **Import quantity (kt)** | **Import share** | **Herfindahl–Hirschman Index** | **Dependency on Russia** |
| --- | --- | --- | --- | --- | --- | --- | --- |
| 42 | GRC | Greece | Non-durum wheat & meslin | 145 | 0.17 | 0.27 | Very low |
| 42 | GRC | Greece | Crude sunflower oil | 0.00004 | 0.000001 | 0.41 | Very low |
| 42 | GRC | Greece | Maize except seed corn | 15 | 0.02 | 0.40 | Very low |
| 43 | GIN | Guinea | Non-durum wheat & meslin | 60 | 0.17 | 0.38 | Very low |
| 43 | GIN | Guinea | Refined sunflower oil | 0.2 | 0.12 | 0.21 | Very low |
| 44 | HUN | Hungary | Refined sunflower oil | 0.00003 | 0.000002 | 0.31 | Very low |
| 45 | ISL | Iceland | Unprocessed barley | 0.00002 | 0.000001 | 0.48 | Vey low |
| 46 | IND | India | Crude sunflower oil | 455 | 0.18 | 0.59 | Low |
| 46 | IND | India | Refined sunflower oil | 0.02 | 0.003 | 0.35 | Very low |
| 47 | IDN | Indonesia | Non-durum wheat & meslin | 48 | 0.005 | 0.22 | Very low |
| 47 | IDN | Indonesia | Refined sunflower oil | 0.02 | 0.003 | 0.22 | Very low |
| 48 | IRQ | Iraq | Non-durum wheat & meslin | 0.1 | 0.01 | 0.71 | Low |
| 48 | IRQ | Iraq | Refined sunflower oil | 0.1 | 0.002 | 0.88 | Low |
| 49 | ISR | Israel | Non-durum wheat & meslin | 201 | 0.09 | 0.23 | Very low |
| 49 | ISR | Israel | Crude sunflower oil | 0.03 | 0.06 | 0.14 | Very low |
| 49 | ISR | Israel | Maize except seed corn | 0.005 | 0.000002 | 0.19 | Very low |
| 49 | ISR | Israel | Refined sunflower oil | 7 | 0.15 | 0.19 | Very low |
| 49 | ISR | Israel | Unprocessed barley | 70 | 0.14 | 0.19 | Vey low |
| 50 | ITA | Italy | Durum wheat | 43 | 0.01 | 0.30 | Very low |
| 50 | ITA | Italy | Non-durum wheat & meslin | 52 | 0.01 | 0.14 | Very low |
| 51 | JPN | Japan | Maize except seed corn | 70 | 0.005 | 0.55 | Low |
| 51 | JPN | Japan | Crude sunflower oil | 1.24 | 0.04 | 0.14 | Very low |
| 52 | JOR | Jordan | Unprocessed barley | 385 | 0.64 | 0.46 | High |
| 52 | JOR | Jordan | Durum wheat | 54 | 0.07 | 0.60 | Low |
| 52 | JOR | Jordan | Crude sunflower oil | 0.0006 | 0.0001 | 0.50 | Low |
| 52 | JOR | Jordan | Maize except seed corn | 1 | 0.001 | 0.54 | Low |
| 52 | JOR | Jordan | Non-durum wheat & meslin | 293 | 0.24 | 0.42 | Very low |
| 52 | JOR | Jordan | Refined sunflower oil | 8 | 0.13 | 0.35 | Very low |
| 53 | KAZ | Kazakhstan | Durum wheat | 70 | 0.99 | 0.98 | Very high |
| 53 | KAZ | Kazakhstan | Non-durum wheat & meslin | 505 | 1.00 | 1.00 | Very high |
| 53 | KAZ | Kazakhstan | Crude sunflower oil | 59 | 1.00 | 1.00 | Very high |
| 53 | KAZ | Kazakhstan | Unprocessed barley | 61 | 0.95 | 0.91 | Very high |

**Table S3.** Continued.

| **No.** | **Code** | **Importer** | **Resource** | **Import quantity (kt)** | **Import share** | **Herfindahl–Hirschman Index** | **Dependency on Russia** |
| --- | --- | --- | --- | --- | --- | --- | --- |
| 53 | KAZ | Kazakhstan | Maize except seed corn | 4 | 0.69 | 0.58 | Very high |
| 53 | KAZ | Kazakhstan | Refined sunflower oil | 50 | 1.00 | 1.00 | Very high |
| 54 | KEN | Kenya | Non-durum wheat & meslin | 596 | 0.31 | 0.21 | Very low |
| 55 | PRK | Korea, DPR | Non-durum wheat & meslin | 50 | 0.99 | 0.98 | Very high |
| 55 | PRK | Korea, DPR | Refined sunflower oil | 0.2 | 0.64 | 0.54 | Very high |
| 56 | KOR | Korea, Republic | Durum wheat | 0.00001 | 0.000001 | 0.97 | Low |
| 56 | KOR | Korea, Republic | Unprocessed barley | 0.05 | 0.001 | 0.85 | Low |
| 56 | KOR | Korea, Republic | Non-durum wheat & meslin | 54 | 0.01 | 0.27 | Very low |
| 56 | KOR | Korea, Republic | Crude sunflower oil | 0.01 | 0.01 | 0.28 | Very low |
| 56 | KOR | Korea, Republic | Maize except seed corn | 237 | 0.02 | 0.19 | Very low |
| 56 | KOR | Korea, Republic | Refined sunflower oil | 4 | 0.10 | 0.27 | Very low |
| 57 | KWT | Kuwait | Non-durum wheat & meslin | 0.4 | 0.001 | 0.76 | Low |
| 57 | KWT | Kuwait | Unprocessed barley | 166 | 0.33 | 0.24 | Vey low |
| 58 | KGZ | Kyrgyzstan | Durum wheat | 12 | 0.55 | 0.51 | Very high |
| 58 | KGZ | Kyrgyzstan | Crude sunflower oil | 1 | 0.55 | 0.50 | Very high |
| 58 | KGZ | Kyrgyzstan | Refined sunflower oil | 32 | 0.96 | 0.93 | Very high |
| 58 | KGZ | Kyrgyzstan | Non-durum wheat & meslin | 74 | 0.57 | 0.49 | High |
| 58 | KGZ | Kyrgyzstan | Unprocessed barley | 0.2 | 0.52 | 0.50 | High |
| 58 | KGZ | Kyrgyzstan | Maize except seed corn | 0.01 | 0.18 | 0.65 | Low |
| 59 | LVA | Latvia | Maize except seed corn | 62 | 0.66 | 0.50 | Very high |
| 59 | LVA | Latvia | Durum wheat | 19 | 0.36 | 0.32 | Very low |
| 59 | LVA | Latvia | Non-durum wheat & meslin | 356 | 0.36 | 0.45 | Very low |
| 59 | LVA | Latvia | Crude sunflower oil | 0.3 | 0.07 | 0.31 | Very low |
| 59 | LVA | Latvia | Refined sunflower oil | 4 | 0.32 | 0.20 | Very low |
| 60 | LBN | Lebanon | Crude sunflower oil | 48 | 0.57 | 0.49 | High |
| 60 | LBN | Lebanon | Durum wheat | 93 | 0.15 | 0.68 | Low |
| 60 | LBN | Lebanon | Non-durum wheat & meslin | 159 | 0.20 | 0.26 | Very low |
| 60 | LBN | Lebanon | Maize except seed corn | 4 | 0.01 | 0.17 | Very low |
| 60 | LBN | Lebanon | Refined sunflower oil | 0.1 | 0.002 | 0.38 | Very low |
| 60 | LBN | Lebanon | Unprocessed barley | 6 | 0.11 | 0.31 | Vey low |
| 61 | LBR | Liberia | Crude sunflower oil | 0.0004 | 0.004 | 0.67 | Low |
| 61 | LBR | Liberia | Non-durum wheat & meslin | 13 | 0.35 | 0.38 | Very low |

**Table S3.** Continued.

| **No.** | **Code** | **Importer** | **Resource** | **Import quantity (kt)** | **Import share** | **Herfindahl–Hirschman Index** | **Dependency on Russia** |
| --- | --- | --- | --- | --- | --- | --- | --- |
| 53 | KAZ | Kazakhstan | Maize except seed corn | 4 | 0.69 | 0.58 | Very high |
| 53 | KAZ | Kazakhstan | Refined sunflower oil | 50 | 1.00 | 1.00 | Very high |
| 54 | KEN | Kenya | Non-durum wheat & meslin | 596 | 0.31 | 0.21 | Very low |
| 55 | PRK | Korea, DPR | Non-durum wheat & meslin | 50 | 0.99 | 0.98 | Very high |
| 55 | PRK | Korea, DPR | Refined sunflower oil | 0.2 | 0.64 | 0.54 | Very high |
| 56 | KOR | Korea, Republic | Durum wheat | 0.00001 | 0.000001 | 0.97 | Low |
| 56 | KOR | Korea, Republic | Unprocessed barley | 0.05 | 0.001 | 0.85 | Low |
| 56 | KOR | Korea, Republic | Non-durum wheat & meslin | 54 | 0.01 | 0.27 | Very low |
| 56 | KOR | Korea, Republic | Crude sunflower oil | 0.01 | 0.01 | 0.28 | Very low |
| 56 | KOR | Korea, Republic | Maize except seed corn | 237 | 0.02 | 0.19 | Very low |
| 56 | KOR | Korea, Republic | Refined sunflower oil | 4 | 0.10 | 0.27 | Very low |
| 57 | KWT | Kuwait | Non-durum wheat & meslin | 0.4 | 0.001 | 0.76 | Low |
| 57 | KWT | Kuwait | Unprocessed barley | 166 | 0.33 | 0.24 | Vey low |
| 58 | KGZ | Kyrgyzstan | Durum wheat | 12 | 0.55 | 0.51 | Very high |
| 58 | KGZ | Kyrgyzstan | Crude sunflower oil | 1 | 0.55 | 0.50 | Very high |
| 58 | KGZ | Kyrgyzstan | Refined sunflower oil | 32 | 0.96 | 0.93 | Very high |
| 58 | KGZ | Kyrgyzstan | Non-durum wheat & meslin | 74 | 0.57 | 0.49 | High |
| 58 | KGZ | Kyrgyzstan | Unprocessed barley | 0.2 | 0.52 | 0.50 | High |
| 58 | KGZ | Kyrgyzstan | Maize except seed corn | 0.01 | 0.18 | 0.65 | Low |
| 59 | LVA | Latvia | Maize except seed corn | 62 | 0.66 | 0.50 | Very high |
| 59 | LVA | Latvia | Durum wheat | 19 | 0.36 | 0.32 | Very low |
| 59 | LVA | Latvia | Non-durum wheat & meslin | 356 | 0.36 | 0.45 | Very low |
| 59 | LVA | Latvia | Crude sunflower oil | 0.3 | 0.07 | 0.31 | Very low |
| 59 | LVA | Latvia | Refined sunflower oil | 4 | 0.32 | 0.20 | Very low |
| 60 | LBN | Lebanon | Crude sunflower oil | 48 | 0.57 | 0.49 | High |
| 60 | LBN | Lebanon | Durum wheat | 93 | 0.15 | 0.68 | Low |
| 60 | LBN | Lebanon | Non-durum wheat & meslin | 159 | 0.20 | 0.26 | Very low |
| 60 | LBN | Lebanon | Maize except seed corn | 4 | 0.01 | 0.17 | Very low |
| 60 | LBN | Lebanon | Refined sunflower oil | 0.1 | 0.002 | 0.38 | Very low |
| 60 | LBN | Lebanon | Unprocessed barley | 6 | 0.11 | 0.31 | Vey low |
| 61 | LBR | Liberia | Crude sunflower oil | 0.0004 | 0.004 | 0.67 | Low |
| 61 | LBR | Liberia | Non-durum wheat & meslin | 13 | 0.35 | 0.38 | Very low |

**Table S3.** Continued.

| **No.** | **Code** | **Importer** | **Resource** | **Import quantity (kt)** | **Import share** | **Herfindahl–Hirschman Index** | **Dependency on Russia** |
| --- | --- | --- | --- | --- | --- | --- | --- |
| 53 | KAZ | Kazakhstan | Maize except seed corn | 4 | 0.69 | 0.58 | Very high |
| 53 | KAZ | Kazakhstan | Refined sunflower oil | 50 | 1.00 | 1.00 | Very high |
| 54 | KEN | Kenya | Non-durum wheat & meslin | 596 | 0.31 | 0.21 | Very low |
| 55 | PRK | Korea, DPR | Non-durum wheat & meslin | 50 | 0.99 | 0.98 | Very high |
| 55 | PRK | Korea, DPR | Refined sunflower oil | 0.2 | 0.64 | 0.54 | Very high |
| 56 | KOR | Korea, Republic | Durum wheat | 0.00001 | 0.000001 | 0.97 | Low |
| 56 | KOR | Korea, Republic | Unprocessed barley | 0.05 | 0.001 | 0.85 | Low |
| 56 | KOR | Korea, Republic | Non-durum wheat & meslin | 54 | 0.01 | 0.27 | Very low |
| 56 | KOR | Korea, Republic | Crude sunflower oil | 0.01 | 0.01 | 0.28 | Very low |
| 56 | KOR | Korea, Republic | Maize except seed corn | 237 | 0.02 | 0.19 | Very low |
| 56 | KOR | Korea, Republic | Refined sunflower oil | 4 | 0.10 | 0.27 | Very low |
| 57 | KWT | Kuwait | Non-durum wheat & meslin | 0.4 | 0.001 | 0.76 | Low |
| 57 | KWT | Kuwait | Unprocessed barley | 166 | 0.33 | 0.24 | Vey low |
| 58 | KGZ | Kyrgyzstan | Durum wheat | 12 | 0.55 | 0.51 | Very high |
| 58 | KGZ | Kyrgyzstan | Crude sunflower oil | 1 | 0.55 | 0.50 | Very high |
| 58 | KGZ | Kyrgyzstan | Refined sunflower oil | 32 | 0.96 | 0.93 | Very high |
| 58 | KGZ | Kyrgyzstan | Non-durum wheat & meslin | 74 | 0.57 | 0.49 | High |
| 58 | KGZ | Kyrgyzstan | Unprocessed barley | 0.2 | 0.52 | 0.50 | High |
| 58 | KGZ | Kyrgyzstan | Maize except seed corn | 0.01 | 0.18 | 0.65 | Low |
| 59 | LVA | Latvia | Maize except seed corn | 62 | 0.66 | 0.50 | Very high |
| 59 | LVA | Latvia | Durum wheat | 19 | 0.36 | 0.32 | Very low |
| 59 | LVA | Latvia | Non-durum wheat & meslin | 356 | 0.36 | 0.45 | Very low |
| 59 | LVA | Latvia | Crude sunflower oil | 0.3 | 0.07 | 0.31 | Very low |
| 59 | LVA | Latvia | Refined sunflower oil | 4 | 0.32 | 0.20 | Very low |
| 60 | LBN | Lebanon | Crude sunflower oil | 48 | 0.57 | 0.49 | High |
| 60 | LBN | Lebanon | Durum wheat | 93 | 0.15 | 0.68 | Low |
| 60 | LBN | Lebanon | Non-durum wheat & meslin | 159 | 0.20 | 0.26 | Very low |
| 60 | LBN | Lebanon | Maize except seed corn | 4 | 0.01 | 0.17 | Very low |
| 60 | LBN | Lebanon | Refined sunflower oil | 0.1 | 0.002 | 0.38 | Very low |
| 60 | LBN | Lebanon | Unprocessed barley | 6 | 0.11 | 0.31 | Vey low |
| 61 | LBR | Liberia | Crude sunflower oil | 0.0004 | 0.004 | 0.67 | Low |
| 61 | LBR | Liberia | Non-durum wheat & meslin | 13 | 0.35 | 0.38 | Very low |

**Table S3.** Continued.

| **No.** | **Code** | **Importer** | **Resource** | **Import quantity (kt)** | **Import share** | **Herfindahl–Hirschman Index** | **Dependency on Russia** |
| --- | --- | --- | --- | --- | --- | --- | --- |
| 62 | LBY | Libya | Non-durum wheat & meslin | 171 | 0.35 | 0.32 | Very low |
| 62 | LBY | Libya | Maize except seed corn | 97 | 0.13 | 0.45 | Very low |
| 62 | LBY | Libya | Unprocessed barley | 200 | 0.25 | 0.39 | Vey low |
| 63 | LTU | Lithuania | Maize except seed corn | 73 | 0.25 | 0.51 | Low |
| 63 | LTU | Lithuania | Durum wheat | 0.2 | 0.01 | 0.38 | Very low |
| 63 | LTU | Lithuania | Non-durum wheat & meslin | 96 | 0.43 | 0.26 | Very low |
| 63 | LTU | Lithuania | Crude sunflower oil | 5 | 0.25 | 0.31 | Very low |
| 63 | LTU | Lithuania | Refined sunflower oil | 1 | 0.07 | 0.27 | Very low |
| 63 | LTU | Lithuania | Unprocessed barley | 3 | 0.05 | 0.21 | Vey low |
| 64 | MDG | Madagascar | Durum wheat | 43 | 0.57 | 0.51 | Very high |
| 64 | MDG | Madagascar | Non-durum wheat & meslin | 36 | 0.52 | 0.50 | Very high |
| 64 | MDG | Madagascar | Crude sunflower oil | 0.02 | 0.01 | 0.49 | Very low |
| 64 | MDG | Madagascar | Refined sunflower oil | 0.1 | 0.02 | 0.22 | Very low |
| 65 | MWI | Malawi | Durum wheat | 58 | 0.73 | 0.58 | Very high |
| 65 | MWI | Malawi | Non-durum wheat & meslin | 47 | 0.69 | 0.52 | Very high |
| 66 | MYS | Malaysia | Refined sunflower oil | 2 | 0.06 | 0.50 | Low |
| 66 | MYS | Malaysia | Non-durum wheat & meslin | 54 | 0.04 | 0.22 | Very low |
| 66 | MYS | Malaysia | Crude sunflower oil | 28 | 0.30 | 0.32 | Very low |
| 66 | MYS | Malaysia | Unprocessed barley | 0.02 | 0.004 | 0.27 | Vey low |
| 67 | MLI | Mali | Non-durum wheat & meslin | 137 | 0.40 | 0.51 | Low |
| 68 | MLT | Malta | Non-durum wheat & meslin | 8 | 0.40 | 0.28 | Very low |
| 69 | MRT | Mauritania | Non-durum wheat & meslin | 162 | 0.24 | 0.20 | Very low |
| 70 | MEX | Mexico | Non-durum wheat & meslin | 96 | 0.02 | 0.62 | Low |
| 71 | MDA | Moldova | Non-durum wheat & meslin | 0 | 0.02 | 0.77 | Low |
| 71 | MDA | Moldova | Crude sunflower oil | 0.001 | 0.08 | 0.79 | Low |
| 71 | MDA | Moldova | Unprocessed barley | 0.0002 | 0.0002 | 0.65 | Low |
| 71 | MDA | Moldova | Refined sunflower oil | 1 | 0.18 | 0.66 | Low |
| 71 | MDA | Moldova | Maize except seed corn | 0.1 | 0.003 | 0.41 | Very low |
| 72 | MNG | Mongolia | Durum wheat | 27 | 1.00 | 1.00 | Very high |
| 72 | MNG | Mongolia | Non-durum wheat & meslin | 144 | 1.00 | 1.00 | Very high |
| 72 | MNG | Mongolia | Crude sunflower oil | 1 | 0.89 | 0.80 | Very high |
| 72 | MNG | Mongolia | Unprocessed barley | 2 | 1.00 | 1.00 | Very high |

**Table S3.** Continued.

| **No.** | **Code** | **Importer** | **Resource** | **Import quantity (kt)** | **Import share** | **Herfindahl–Hirschman Index** | **Dependency on Russia** |
| --- | --- | --- | --- | --- | --- | --- | --- |
| 72 | MNG | Mongolia | Maize except seed corn | 8 | 0.99 | 0.98 | Very high |
| 72 | MNG | Mongolia | Refined sunflower oil | 15 | 0.94 | 0.89 | Very high |
| 73 | MNE | Montenegro | Crude sunflower oil | 0.02 | 0.02 | 0.37 | Very low |
| 74 | MAR | Morocco | Refined sunflower oil | 0.002 | 0.0005 | 0.64 | Low |
| 74 | MAR | Morocco | Non-durum wheat & meslin | 426 | 0.10 | 0.23 | Very low |
| 74 | MAR | Morocco | Unprocessed barley | 25 | 0.02 | 0.21 | Vey low |
| 75 | MOZ | Mozambique | Durum wheat | 28 | 1.00 | 1.00 | Very high |
| 75 | MOZ | Mozambique | Non-durum wheat & meslin | 204 | 0.23 | 0.12 | Very low |
| 76 | MMR | Myanmar | Non-durum wheat & meslin | 4 | 0.01 | 0.36 | Very low |
| 76 | MMR | Myanmar | Refined sunflower oil | 2 | 0.04 | 0.26 | Very low |
| 77 | NAM | Namibia | Non-durum wheat & meslin | 82 | 0.66 | 0.46 | High |
| 78 | NPL | Nepal | Crude sunflower oil | 3 | 0.09 | 0.84 | Low |
| 78 | NPL | Nepal | Refined sunflower oil | 0.02 | 0.01 | 0.36 | Very low |
| 79 | NLD | Netherlands | Crude sunflower oil | 0.000002 | 0.000000003 | 0.74 | Low |
| 79 | NLD | Netherlands | Non-durum wheat & meslin | 1 | 0.0004 | 0.32 | Very low |
| 79 | NLD | Netherlands | Maize except seed corn | 6 | 0.001 | 0.30 | Very low |
| 79 | NLD | Netherlands | Refined sunflower oil | 0.001 | 0.000004 | 0.23 | Very low |
| 80 | NCL | New Caledonia | Refined sunflower oil | 0.2 | 0.08 | 0.25 | Very low |
| 81 | NZL | New Zealand | Crude sunflower oil | 0.001 | 0.0001 | 0.62 | Low |
| 81 | NZL | New Zealand | Refined sunflower oil | 0.0001 | 0.00001 | 0.28 | Very low |
| 82 | NIC | Nicaragua | Durum wheat | 102 | 0.87 | 0.76 | Very high |
| 82 | NIC | Nicaragua | Non-durum wheat & meslin | 102 | 0.57 | 0.45 | High |
| 82 | NIC | Nicaragua | Refined sunflower oil | 0.1 | 0.37 | 0.28 | Very low |
| 83 | NGA | Nigeria | Non-durum wheat & meslin | 998 | 0.18 | 0.16 | Very low |
| 84 | MKD | North Macedonia | Durum wheat | 0.1 | 0.02 | 0.87 | Low |
| 84 | MKD | North Macedonia | Non-durum wheat & meslin | 0.5 | 0.006 | 0.71 | Low |
| 85 | NOR | Norway | Non-durum wheat & meslin | 24 | 0.08 | 0.24 | Very low |
| 85 | NOR | Norway | Maize except seed corn | 1 | 0.01 | 0.46 | Very low |
| 85 | NOR | Norway | Refined sunflower oil | 0.0003 | 0.00003 | 0.14 | Very low |
| 86 | OMN | Oman | Non-durum wheat & meslin | 429 | 0.66 | 0.46 | High |
| 86 | OMN | Oman | Crude sunflower oil | 3 | 0.14 | 0.73 | Low |
| 86 | OMN | Oman | Maize except seed corn | 26 | 0.07 | 0.31 | Very low |

**Table S3.** Continued.

| **No.** | **Code** | **Importer** | **Resource** | **Import quantity (kt)** | **Import share** | **Herfindahl–Hirschman Index** | **Dependency on Russia** |
| --- | --- | --- | --- | --- | --- | --- | --- |
| 86 | OMN | Oman | Unprocessed barley | 27 | 0.17 | 0.21 | Vey low |
| 87 | PAK | Pakistan | Non-durum wheat & meslin | 685 | 0.58 | 0.39 | High |
| 87 | PAK | Pakistan | Durum wheat | 499 | 0.29 | 0.49 | Very low |
| 88 | PAN | Panama | Crude sunflower oil | 0.00004 | 0.002 | 0.47 | Very low |
| 89 | PHL | Philippines | Non-durum wheat & meslin | 549 | 0.09 | 0.30 | Very low |
| 90 | POL | Poland | Crude sunflower oil | 0.001 | 0.00001 | 0.54 | Low |
| 90 | POL | Poland | Non-durum wheat & meslin | 1 | 0.002 | 0.34 | Very low |
| 90 | POL | Poland | Refined sunflower oil | 0.003 | 0.00002 | 0.22 | Very low |
| 91 | QAT | Qatar | Maize except seed corn | 4 | 0.04 | 0.54 | Low |
| 91 | QAT | Qatar | Non-durum wheat & meslin | 23 | 0.16 | 0.22 | Very low |
| 91 | QAT | Qatar | Unprocessed barley | 25 | 0.09 | 0.41 | Vey low |
| 92 | ROU | Romania | Non-durum wheat & meslin | 56 | 0.04 | 0.38 | Very low |
| 92 | ROU | Romania | Maize except seed corn | 0.1 | 0.00003 | 0.37 | Very low |
| 92 | ROU | Romania | Refined sunflower oil | 0.004 | 0.0001 | 0.19 | Very low |
| 93 | RWA | Rwanda | Non-durum wheat & meslin | 65 | 0.93 | 0.86 | Very high |
| 94 | SAU | Saudi Arabia | Durum wheat | 62 | 0.08 | 0.39 | Very low |
| 94 | SAU | Saudi Arabia | Non-durum wheat & meslin | 247 | 0.08 | 0.33 | Very low |
| 94 | SAU | Saudi Arabia | Crude sunflower oil | 35 | 0.36 | 0.44 | Very low |
| 94 | SAU | Saudi Arabia | Maize except seed corn | 0.1 | 0.00002 | 0.34 | Very low |
| 94 | SAU | Saudi Arabia | Unprocessed barley | 1,873 | 0.36 | 0.17 | Vey low |
| 95 | SEN | Senegal | Durum wheat | 284 | 0.61 | 0.45 | High |
| 95 | SEN | Senegal | Non-durum wheat & meslin | 107 | 0.35 | 0.47 | Very low |
| 95 | SEN | Senegal | Crude sunflower oil | 1 | 0.27 | 0.40 | Very low |
| 96 | SRB | Serbia | Refined sunflower oil | 13 | 0.53 | 0.34 | High |
| 96 | SRB | Serbia | Non-durum wheat & meslin | 0.1 | 0.004 | 0.53 | Low |
| 96 | SRB | Serbia | Maize except seed corn | 0.1 | 0.03 | 0.53 | Low |
| 97 | SYC | Seychelles | Refined sunflower oil | 0.2 | 0.04 | 0.47 | Very low |
| 98 | SGP | Singapore | Refined sunflower oil | 1 | 0.04 | 0.28 | Very low |
| 99 | SVN | Slovenia | Crude sunflower oil | 0.00002 | 0.000001 | 0.51 | Low |
| 99 | SVN | Slovenia | Refined sunflower oil | 0.0002 | 0.00001 | 0.24 | Very low |
| 100 | SOM | Somalia | Non-durum wheat & meslin | 30 | 0.39 | 0.44 | Very low |
| 101 | ZAF | South Africa | Durum wheat | 0.00002 | 0.0001 | 0.74 | Low |

**Table S3.** Continued.

| **No.** | **Code** | **Importer** | **Resource** | **Import quantity (kt)** | **Import share** | **Herfindahl–Hirschman Index** | **Dependency on Russia** |
| --- | --- | --- | --- | --- | --- | --- | --- |
| 101 | ZAF | South Africa | Maize except seed corn | 0.1 | 0.001 | 0.97 | Low |
| 101 | ZAF | South Africa | Non-durum wheat & meslin | 572 | 0.26 | 0.21 | Very low |
| 102 | ESP | Spain | Non-durum wheat & meslin | 21 | 0.01 | 0.16 | Very low |
| 103 | LKA | Sri Lanka | Non-durum wheat & meslin | 505 | 0.36 | 0.29 | Very low |
| 103 | LKA | Sri Lanka | Refined sunflower oil | 0.02 | 0.01 | 0.50 | Very low |
| 104 | PSE | State of Palestine | Durum wheat | 9 | 1.00 | 1.00 | Very high |
| 104 | PSE | State of Palestine | Non-durum wheat & meslin | 5 | 0.13 | 0.47 | Very low |
| 104 | PSE | State of Palestine | Refined sunflower oil | 1 | 0.05 | 0.36 | Very low |
| 105 | SDN | Sudan | Crude sunflower oil | 74 | 0.58 | 0.51 | Very high |
| 105 | SDN | Sudan | Non-durum wheat & meslin | 1,333 | 0.55 | 0.34 | High |
| 106 | SWE | Sweden | Maize except seed corn | 3 | 0.05 | 0.30 | Very low |
| 107 | CHE | Switzerland | Non-durum wheat & meslin | 55 | 0.12 | 0.22 | Very low |
| 107 | CHE | Switzerland | Refined sunflower oil | 0.0001 | 0.000002 | 0.16 | Very low |
| 108 | TJK | Tajikistan | Refined sunflower oil | 38 | 0.96 | 0.92 | Very high |
| 108 | TJK | Tajikistan | Crude sunflower oil | 15 | 0.55 | 0.47 | High |
| 108 | TJK | Tajikistan | Durum wheat | 0.04 | 0.01 | 0.99 | Low |
| 108 | TJK | Tajikistan | Non-durum wheat & meslin | 28 | 0.03 | 0.90 | Low |
| 108 | TJK | Tajikistan | Unprocessed barley | 0.01 | 0.0002 | 1.00 | Low |
| 108 | TJK | Tajikistan | Maize except seed corn | 0.1 | 0.01 | 0.81 | Low |
| 109 | TZA | Tanzania | Non-durum wheat & meslin | 701 | 0.71 | 0.52 | Very high |
| 110 | THA | Thailand | Non-durum wheat & meslin | 308 | 0.10 | 0.16 | Very low |
| 110 | THA | Thailand | Refined sunflower oil | 0.1 | 0.01 | 0.31 | Very low |
| 101 | ZAF | South Africa | Maize except seed corn | 0.1 | 0.001 | 0.97 | Low |
| 101 | ZAF | South Africa | Non-durum wheat & meslin | 572 | 0.26 | 0.21 | Very low |
| 102 | ESP | Spain | Non-durum wheat & meslin | 21 | 0.01 | 0.16 | Very low |
| 103 | LKA | Sri Lanka | Non-durum wheat & meslin | 505 | 0.36 | 0.29 | Very low |
| 103 | LKA | Sri Lanka | Refined sunflower oil | 0.02 | 0.01 | 0.50 | Very low |
| 104 | PSE | State of Palestine | Durum wheat | 9 | 1.00 | 1.00 | Very high |
| 104 | PSE | State of Palestine | Non-durum wheat & meslin | 5 | 0.13 | 0.47 | Very low |
| 104 | PSE | State of Palestine | Refined sunflower oil | 1 | 0.05 | 0.36 | Very low |
| 105 | SDN | Sudan | Crude sunflower oil | 74 | 0.58 | 0.51 | Very high |
| 105 | SDN | Sudan | Non-durum wheat & meslin | 1,333 | 0.55 | 0.34 | High |

**Table S3.** Continued.

| **No.** | **Code** | **Importer** | **Resource** | **Import quantity (kt)** | **Import share** | **Herfindahl–Hirschman Index** | **Dependency on Russia** |
| --- | --- | --- | --- | --- | --- | --- | --- |
| 106 | SWE | Sweden | Maize except seed corn | 3 | 0.05 | 0.30 | Very low |
| 107 | CHE | Switzerland | Non-durum wheat & meslin | 55 | 0.12 | 0.22 | Very low |
| 107 | CHE | Switzerland | Refined sunflower oil | 0.0001 | 0.000002 | 0.16 | Very low |
| 108 | TJK | Tajikistan | Refined sunflower oil | 38 | 0.96 | 0.92 | Very high |
| 108 | TJK | Tajikistan | Crude sunflower oil | 15 | 0.55 | 0.47 | High |
| 108 | TJK | Tajikistan | Durum wheat | 0.04 | 0.01 | 0.99 | Low |
| 108 | TJK | Tajikistan | Non-durum wheat & meslin | 28 | 0.03 | 0.90 | Low |
| 108 | TJK | Tajikistan | Unprocessed barley | 0.01 | 0.0002 | 1.00 | Low |
| 108 | TJK | Tajikistan | Maize except seed corn | 0.1 | 0.01 | 0.81 | Low |
| 109 | TZA | Tanzania | Non-durum wheat & meslin | 701 | 0.71 | 0.52 | Very high |
| 110 | THA | Thailand | Non-durum wheat & meslin | 308 | 0.10 | 0.16 | Very low |
| 110 | THA | Thailand | Refined sunflower oil | 0.1 | 0.01 | 0.31 | Very low |
| 111 | TGO | Togo | Durum wheat | 32 | 0.68 | 0.57 | Very high |
| 111 | TGO | Togo | Non-durum wheat & meslin | 57 | 0.58 | 0.48 | High |
| 111 | TGO | Togo | Refined sunflower oil | 0.05 | 0.01 | 0.43 | Very low |
| 112 | TUN | Tunisia | Non-durum wheat & meslin | 111 | 0.09 | 0.59 | Low |
| 112 | TUN | Tunisia | Crude sunflower oil | 4 | 0.33 | 0.54 | Low |
| 112 | TUN | Tunisia | Unprocessed barley | 263 | 0.27 | 0.27 | Vey low |
| 106 | SWE | Sweden | Maize except seed corn | 3 | 0.05 | 0.30 | Very low |
| 107 | CHE | Switzerland | Non-durum wheat & meslin | 55 | 0.12 | 0.22 | Very low |
| 107 | CHE | Switzerland | Refined sunflower oil | 0.0001 | 0.000002 | 0.16 | Very low |
| 108 | TJK | Tajikistan | Refined sunflower oil | 38 | 0.96 | 0.92 | Very high |
| 108 | TJK | Tajikistan | Crude sunflower oil | 15 | 0.55 | 0.47 | High |
| 108 | TJK | Tajikistan | Durum wheat | 0.04 | 0.01 | 0.99 | Low |
| 108 | TJK | Tajikistan | Non-durum wheat & meslin | 28 | 0.03 | 0.90 | Low |
| 108 | TJK | Tajikistan | Unprocessed barley | 0.01 | 0.0002 | 1.00 | Low |
| 108 | TJK | Tajikistan | Maize except seed corn | 0.1 | 0.01 | 0.81 | Low |
| 109 | TZA | Tanzania | Non-durum wheat & meslin | 701 | 0.71 | 0.52 | Very high |
| 110 | THA | Thailand | Non-durum wheat & meslin | 308 | 0.10 | 0.16 | Very low |
| 110 | THA | Thailand | Refined sunflower oil | 0.1 | 0.01 | 0.31 | Very low |
| 111 | TGO | Togo | Durum wheat | 32 | 0.68 | 0.57 | Very high |
| 111 | TGO | Togo | Non-durum wheat & meslin | 57 | 0.58 | 0.48 | High |

**Table S3.** Continued.

| **No.** | **Code** | **Importer** | **Resource** | **Import quantity (kt)** | **Import share** | **Herfindahl–Hirschman Index** | **Dependency on Russia** |
| --- | --- | --- | --- | --- | --- | --- | --- |
| 111 | TGO | Togo | Refined sunflower oil | 0.05 | 0.01 | 0.43 | Very low |
| 112 | TUN | Tunisia | Non-durum wheat & meslin | 111 | 0.09 | 0.59 | Low |
| 112 | TUN | Tunisia | Crude sunflower oil | 4 | 0.33 | 0.54 | Low |
| 112 | TUN | Tunisia | Unprocessed barley | 263 | 0.27 | 0.27 | Vey low |
| 113 | TUR | Turkey | Non-durum wheat & meslin | 7,025 | 0.75 | 0.58 | Very high |
| 113 | TUR | Turkey | Crude sunflower oil | 667 | 0.79 | 0.64 | Very high |
| 113 | TUR | Turkey | Refined sunflower oil | 0.1 | 0.003 | 0.94 | Low |
| 113 | TUR | Turkey | Durum wheat | 20 | 0.02 | 0.36 | Very low |
| 113 | TUR | Turkey | Maize except seed corn | 654 | 0.25 | 0.25 | Very low |
| 113 | TUR | Turkey | Unprocessed barley | 316 | 0.33 | 0.26 | Vey low |
| 114 | TKM | Turkmenistan | Crude sunflower oil | 5 | 0.85 | 0.74 | Very high |
| 114 | TKM | Turkmenistan | Maize except seed corn | 8 | 0.96 | 0.92 | Very high |
| 114 | TKM | Turkmenistan | Refined sunflower oil | 32 | 0.90 | 0.82 | Very high |
| 114 | TKM | Turkmenistan | Non-durum wheat & meslin | 17 | 0.25 | 0.63 | Low |
| 115 | UGA | Uganda | Non-durum wheat & meslin | 203 | 0.33 | 0.22 | Very low |
| 116 | UKR | Ukraine | Unprocessed barley | 9 | 0.86 | 0.74 | Very high |
| 116 | UKR | Ukraine | Refined sunflower oil | 17 | 1.00 | 0.99 | Very high |
| 116 | UKR | Ukraine | Crude sunflower oil | 0.1 | 0.65 | 0.47 | High |
| 116 | UKR | Ukraine | Durum wheat | 0.1 | 0.48 | 0.39 | Very low |
| 116 | UKR | Ukraine | Non-durum wheat & meslin | 11 | 0.23 | 0.38 | Very low |
| 116 | UKR | Ukraine | Maize except seed corn | 0.1 | 0.02 | 0.39 | Very low |
| 117 | ARE | United Arab Emirates | Crude sunflower oil | 1 | 0.02 | 0.92 | Low |
| 117 | ARE | United Arab Emirates | Non-durum wheat & meslin | 686 | 0.47 | 0.29 | Very low |
| 117 | ARE | United Arab Emirates | Maize except seed corn | 21 | 0.04 | 0.28 | Very low |
| 117 | ARE | United Arab Emirates | Refined sunflower oil | 0.03 | 0.001 | 0.43 | Very low |
| 117 | ARE | United Arab Emirates | Unprocessed barley | 103 | 0.22 | 0.22 | Vey low |
| 118 | GBR | United Kingdom | Non-durum wheat & meslin | 17 | 0.01 | 0.20 | Very low |
| 118 | GBR | United Kingdom | Maize except seed corn | 38 | 0.02 | 0.17 | Very low |
| 119 | USA | United States | Unprocessed barley | 0.02 | 0.0001 | 0.98 | Low |
| 119 | USA | United States | Crude sunflower oil | 0.2 | 0.003 | 0.23 | Very low |
| 119 | USA | United States | Refined sunflower oil | 0.1 | 0.001 | 0.24 | Very low |
| 120 | UZB | Uzbekistan | Refined sunflower oil | 189 | 0.91 | 0.84 | Very high |

**Table S3.** Continued.

| **No.** | **Code** | **Importer** | **Resource** | **Import quantity (kt)** | **Import share** | **Herfindahl–Hirschman Index** | **Dependency on Russia** |
| --- | --- | --- | --- | --- | --- | --- | --- |
| 120 | UZB | Uzbekistan | Non-durum wheat & meslin | 21 | 0.01 | 0.98 | Low |
| 120 | UZB | Uzbekistan | Crude sunflower oil | 11 | 0.24 | 0.63 | Low |
| 120 | UZB | Uzbekistan | Unprocessed barley | 4 | 0.06 | 0.66 | Low |
| 120 | UZB | Uzbekistan | Maize except seed corn | 0.03 | 0.0005 | 0.94 | Low |
| 121 | VNM | Vietnam | Crude sunflower oil | 0.1 | 0.54 | 0.40 | High |
| 121 | VNM | Vietnam | Refined sunflower oil | 8 | 0.51 | 0.34 | High |
| 121 | VNM | Vietnam | Unprocessed barley | 2 | 0.01 | 0.70 | Low |
| 121 | VNM | Vietnam | Non-durum wheat & meslin | 658 | 0.21 | 0.17 | Very low |
| 121 | VNM | Vietnam | Maize except seed corn | 507 | 0.04 | 0.48 | Very low |
| 122 | YEM | Yemen | Non-durum wheat & meslin | 796 | 0.26 | 0.23 | Very low |
| 123 | ZWE | Zimbabwe | Durum wheat | 10 | 0.05 | 0.17 | Very low |

Data on import quantity were obtained from Chatham House (2021), ‘resourcetrade.earth’, https://resourcetrade.earth/

Table S4. Top exported wheat, maize, and sunflower products from Turkey and Egypt and their destinations in 2020.

| **Commodities** | **Exporter** | **Importer** | **Import quantity  (kt)** | **Export share**  **in total exports** | **Import share**  **in total imports** |
| --- | --- | --- | --- | --- | --- |
| Wheat or meslin flour | Turkey | Iraq | 1,345 | 46% | 99% |
|  | Turkey | Yemen | 294 | 10% | 81% |
|  | Turkey | Syria | 200 | 7% | 80% |
|  | Turkey | Angola | 164 | 6% | 79% |
|  | Turkey | Venezuela | 156 | 5% | 71% |
|  | Turkey | Somalia | 113 | 4% | 54% |
|  | Turkey | Benin | 112 | 4% | 88% |
|  | Turkey | Ghana | 65 | 2% | 98% |
|  | Turkey | Madagascar | 51 | 2% | 47% |
|  | Turkey | Others | 435 | 15% |  |
|  |  | **Subtotal** | **2,935** | **100%** |  |
|  | Egypt | Eritrea | 77 | 26% | 100% |
|  | Egypt | Somalia | 61 | 20% | 29% |
|  | Egypt | Madagascar | 53 | 17% | 49% |
|  | Egypt | Yemen | 48 | 16% | 13% |
|  | Egypt | Syria | 23 | 8% | 9% |
|  | Egypt | Others | 40 | 13% |  |
|  |  | **Subtotal** | **301** | **100%** |  |
| Sunflower oil, crude | Turkey | Iraq | 59 | 58% | 11% |
|  | Turkey | Iran | 30 | 29% | 50% |
|  | Turkey | Others | 13 | 13% |  |
|  |  | **Subtotal** | **102** | **100%** |  |
|  | Egypt | Saudi Arabia | 7 | 24% | 7% |
|  | Egypt | Rwanda | 5 | 16% | 100% |
|  | Egypt | Syria | 4 | 14% | 92% |
|  | Egypt | State of Palestine | 3 | 10% | 99% |
|  | Egypt | Jordan | 3 | 10% | 63% |
|  | Egypt | Eritrea | 2 | 5% | 98% |
|  |  | Yemen | 1 | 4% | 79% |
|  |  | Others | 5 | 17% |  |
|  |  | **Subtotal** | **29** | **100%** |  |
| Sunflower oil, refined | Turkey | Djibouti | 190 | 25% | 96% |
|  | Turkey | Ethiopia | 157 | 20% | 69% |
|  | Turkey | Syria | 57 | 7% | 69% |
|  | Turkey | Iraq | 54 | 7% | 94% |
|  | Turkey | Yemen | 50 | 6% | 84% |
|  | Turkey | Libya | 49 | 6% | 71% |
|  | Turkey | Somalia | 25 | 3% | 85% |
|  | Turkey | Others | 193 | 25% |  |
|  |  | **Subtotal** | **775** | **100%** |  |
|  | Egypt | Jordan | 5 | 23% | 8% |
|  | Egypt | Ethiopia | 3 | 13% | 1% |
|  | Egypt | Rwanda | 2 | 11% | 60% |
|  | Egypt | Syria | 2 | 10% | 3% |
|  | Egypt | Eritrea | 2 | 8% | 96% |
|  | Egypt | Madagascar | 2 | 8% | 35% |
|  |  | Others | 6 | 27% |  |
|  |  | **Subtotal** | **22** | **100%** |  |

Data on import quantity were obtained from Chatham House (2021), ‘resourcetrade.earth’, <https://resourcetrade.earth/>

Fig. S2. Share of calorie-based food supply in countries that import over 70% of wheat flour, corn starch, and sunflower oil in total imports from Egypt and Turkey in 2020 (*Source*: FAO.[FAOSTAT]. License: CC BY-NC-SA 3.0 IGO. https://www.fao.org/faostat/en/#data).

Table S5. Barley supply and its feed utilization with the share of use over domestic supply quantity in 2019 (in tonnes).

| Country | Production | Import Quantity | Export Quantity | Stock Variation | Domestic supply quantity | Feed use | Share of feed use |
| --- | --- | --- | --- | --- | --- | --- | --- |
| Afghanistan | 123,576 | 11,927 | 3,485 | 6,499 | 125,519 | 88,000 | 70% |
| Albania | 7,942 | 6,058 |  | 0 | 14,000 | 7,608 | 54% |
| Algeria | 1,647,746 | 122,297 |  | 0 | 1,770,043 | 1,101,551 | 62% |
| Armenia | 68,333 | 23,322 |  | 8,389 | 83,266 | 73,879 | 89% |
| Australia | 8,818,946 | 51 | 2,872,279 | -132,000 | 6,078,718 | 3,982,925 | 66% |
| Austria | 828,130 | 226,025 | 52,705 | -122,930 | 1,124,380 | 595,344 | 53% |
| Azerbaijan | 988,184 | 14,160 | 16,121 | 6,002 | 980,221 | 809,873 | 83% |
| Bangladesh | 157 | 204 |  | -27 | 388 | 77 | 20% |
| Belarus | 1,098,189 | 225,660 | 897 | -1,000 | 1,323,952 | 992,111 | 75% |
| Belgium | 401,060 | 2,011,363 | 214,132 | -19,887 | 2,218,178 | 1001437 | 45% |
| Bolivia (Pluractional State of) | 47,951 |  |  | 0 | 47,951 | 28,785 | 60% |
| Bosnia and Herzegovina | 74,462 | 28,591 | 24 | -3,000 | 106,029 | 73,393 | 69% |
| Botswana |  | 15 |  | 0 | 15 | 15 | 100% |
| Bulgaria | 563,150 | 1,713 | 267,290 | -4,765 | 302,338 | 117135 | 39% |
| Canada | 10,382,600 | 41,891 | 2,183,447 | -506,055 | 8,747,099 | 5,527,561 | 63% |
| Chile | 210,890 | 52,123 | 3 | -64,018 | 327,028 | 53,485 | 16% |
| China, Hong Kong SAR |  | 49 | 2 | 0 | 47 | 47 | 100% |
| Congo |  | 1,563 |  | 0 | 1,563 | 1563 | 100% |
| Costa Rica |  | 46 |  | 0 | 46 | 46 | 100% |
| Croatia | 278,600 | 9,976 | 36,714 | -4,793 | 256,655 | 101614 | 40% |
| Cuba |  | 100 |  | 0 | 100 | 97 | 97% |
| Croatia | 278,600 | 9,976 | 36,714 | -4,793 | 256,655 | 101614 | 40% |
| Cuba |  | 100 |  | 0 | 100 | 97 | 97% |
| Cyprus | 30,400 | 133,575 |  | 40,149 | 123,826 | 184,701 | 149% |
| Czechia | 1,718,060 | 112,546 | 215,239 | -133,798 | 1,749,165 | 457,631 | 26% |
| Denmark | 3,624,560 | 103,776 | 522,438 | -83,147 | 3,289,045 | 2,612,000 | 79% |
| Egypt | 107,000 | 5,498 | 3 | 0 | 112,495 | 66,841 | 59% |
| El Salvador |  | 10 |  | 0 | 10 | 10 | 100% |
| Estonia | 523,140 | 14,842 | 362,656 | 216 | 175,110 | 110,045 | 63% |
| Finland | 1,701,960 | 266 | 132,857 | 27,830 | 1,541,539 | 1,127,311 | 73% |
| France | 13,565,420 | 93,466 | 7,171,937 | -350,306 | 6,837,255 | 3,473,987 | 51% |
| Germany | 11,591,500 | 1,268,081 | 1,580,572 | -254,972 | 11,533,981 | 6,174,198 | 54% |

**Table S5.** Continued.

| Country | Production | Import Quantity | Export Quantity | Stock Variation | Domestic supply quantity | Feed use | Share of feed use |
| --- | --- | --- | --- | --- | --- | --- | --- |
| Guatemala | 29 | 212 | 9 | -1 | 233 | 229 | 98% |
| Guyana |  | 37 |  | 0 | 37 | 37 | 100% |
| Hungary | 1,383,260 | 20,713 | 420,266 | 0 | 983,707 | 775,402 | 79% |
| Iceland | 7,900 | 12,654 |  | -10,092 | 30,646 | 7,900 | 26% |
| India | 1,633,070 | 256,369 | 1,861 | -19,261 | 1,906,839 | 207,397 | 11% |
| Iran (Islamic Republic of) | 3,600,000 | 3,287,269 |  | -71,440 | 6,958,709 | 6,511,877 | 94% |
| Iraq | 1,518,471 | 45,533 |  | -355,451 | 1,919,455 | 851,479 | 44% |
| Ireland | 1,479,600 | 138,360 | 52,846 | 72,361 | 1,492,753 | 1,363,152 | 91% |
| Israel | 5,000 | 260,791 | 36 | -10,351 | 276,106 | 243,970 | 88% |
| Italy | 1,104,990 | 497,102 | 4,104 | -200,000 | 1,797,988 | 1,054,604 | 59% |
| Japan | 222,700 | 1,147,828 | 101 | -39,541 | 1,409,968 | 914,000 | 65% |
| Jordan | 66,618 | 860,236 |  | 4,622 | 922,232 | 872,680 | 95% |
| Kazakhstan | 3,830,069 | 38,485 | 1,640,083 | 0 | 2,228,471 | 666,314 | 30% |
| Kuwait | 2,726 | 330,399 |  | -29 | 333,154 | 316,391 | 95% |
| Japan | 222,700 | 1,147,828 | 101 | -39,541 | 1,409,968 | 914,000 | 65% |
| Jordan | 66,618 | 860,236 |  | 4,622 | 922,232 | 872,680 | 95% |
| Kazakhstan | 3,830,069 | 38,485 | 1,640,083 | 0 | 2,228,471 | 666,314 | 30% |
| Kuwait | 2,726 | 330,399 |  | -29 | 333,154 | 316,391 | 95% |
| Kyrgyzstan | 465,864 | 407 | 100 | 0 | 466,171 | 378,648 | 81% |
| Lao PDR |  | 1,745 |  | 0 | 1,745 | 325 | 19% |
| Lebanon | 32,000 | 107,214 | 1,935 | -21,648 | 158,927 | 100,782 | 63% |
| Libya | 70,000 | 569,236 |  | 0 | 639,236 | 389,474 | 61% |
| Lithuania | 588,450 | 43,769 | 292,639 | 68,025 | 271,555 | 161,802 | 60% |
| Luxembourg | 36,050 | 5,368 | 13,747 | 0 | 27,671 | 25,000 | 90% |
| Malta |  | 8,315 |  | -28 | 8,343 | 8,231 | 99% |
| Morocco | 1,161,183 | 382,089 |  | 696,567 | 846,705 | 745,366 | 88% |
| Namibia |  | 215 |  | 0 | 215 | 215 | 100% |
| Netherlands | 242,370 | 2,156,518 | 96,336 | 109,129 | 2,193,423 | 2,008,452 | 92% |
| New Caledonia |  | 916 |  | 0 | 916 | 916 | 100% |
| New Zealand | 383,651 | 5,529 | 115 | -6,000 | 395,065 | 317,359 | 80% |
| Nigeria |  | 3,878 |  | 0 | 3,878 | 3,878 | 100% |
| North Macedonia | 138,453 | 7,822 | 3,469 | 4,000 | 138,806 | 129,295 | 93% |

**Table S5.** Continued.

| Country | Production | Import Quantity | Export Quantity | Stock Variation | Domestic supply quantity | Feed use | Share of feed use |
| --- | --- | --- | --- | --- | --- | --- | --- |
| Norway | 581,000 | 45,771 | 751 | -45,000 | 671,020 | 539,000 | 80% |
| Oman | 1,677 | 180,754 | 92 | -58 | 182,397 | 182,206 | 100% |
| Pakistan | 54,685 | 8,479 | 1 | -3,022 | 66,185 | 4,326 | 7% |
| Panama |  | 112 |  | 0 | 112 | 112 | 100% |
| Papua New Guinea |  | 499 |  | 0 | 499 | 326 | 65% |
| Peru | 215,509 | 121,802 | 30 | 7,000 | 330,281 | 60,000 | 18% |
| Poland | 3,311,570 | 215,019 | 82,986 | 0 | 3,443,603 | 2,309,561 | 67% |
| Portugal | 53,300 | 347,560 | 25,932 | -7,655 | 382,583 | 188,843 | 49% |
| Republic of Korea | 136,915 | 67,708 | 83 | -6,378 | 210,918 | 9,979 | 5% |
| Romania | 1,879,950 | 148,443 | 1,123,914 | -280,000 | 1,184,479 | 104,766 | 9% |
| Russian Federation | 20,489,088 | 34,911 | 3,940,653 | -103,000 | 16,686,346 | 9,563,865 | 57% |
| Saudi Arabia | 627,982 | 3,905,759 |  | -82,749 | 4,616,490 | 4,310,736 | 93% |
| Serbia | 373,340 | 5,139 | 47,627 | 6,000 | 324,852 | 177,166 | 55% |
| Slovenia | 102,480 | 24,724 | 14,744 | -884 | 113,344 | 79,963 | 71% |
| South Africa | 345,000 | 12,027 | 2,101 | -130,000 | 484,926 | 25,000 | 5% |
| Spain | 7,744,150 | 1,221,055 | 151,462 | -273,357 | 9,087,100 | 7,099,189 | 78% |
| Sweden | 1,546,500 | 108,819 | 219,502 | -215,719 | 1,651,536 | 843,269 | 51% |
| Switzerland | 190,167 | 49,185 | 434 | 6,000 | 232,918 | 218,721 | 94% |
| Syrian Arab Republic | 3,053,124 | 15,937 | 52,500 | -816,592 | 3,833,153 | 1,857,597 | 48% |
| Tajikistan | 156,240 | 3,254 |  | 0 | 159,494 | 91,999 | 58% |
| Tunisia | 940,000 | 542,722 | 375 | -245,000 | 1,727,347 | 1,040,317 | 60% |
| Turkey | 7,600,000 | 562,777 | 41,188 | -1,116,045 | 9,237,634 | 5,000,000 | 54% |
| Turkmenistan | 19,357 | 549 |  | 0 | 19,906 | 16,318 | 82% |
| Uganda |  | 24,844 | 1 | -38 | 24,881 | 1,524 | 6% |
| Ukraine | 8,916,780 | 9,533 | 4,143,432 | -43,000 | 4,825,881 | 3,045,100 | 63% |
| United Arab Emirates |  | 301,250 | 103,426 | 0 | 197,824 | 197,822 | 100% |
| United Kingdom | 8,048,000 | 57,272 | 1,666,221 | -593,059 | 7,032,110 | 3,787,000 | 54% |
| United States of America | 3,691,860 | 142,375 | 119,029 | 137,000 | 3,578,206 | 836,000 | 23% |
| Uzbekistan | 133,496 | 71,854 |  | 0 | 205,350 | 82,708 | 40% |
| Yemen | 26,933 |  |  | -677 | 27,610 | 21,184 | 77% |

Data source: FAO.[FAOSTAT]. License: CC BY-NC-SA 3.0 IGO. Extracted from: [https://www.fao.org/faostat/en/#data]. Data of Access: [3-June-2022].

Table S6. Maize supply and its feed utilization with the share of use over domestic supply quantity in 2019 (in tonnes).

| Country | Production | Import Quantity | Export Quantity | Stock Variation | Domestic supply quantity | Feed use | Share of feed use |
| --- | --- | --- | --- | --- | --- | --- | --- |
| Afghanistan | 184,671 | 13,922 | 16 | 0 | 198,577 | 118,000 | 59% |
| Albania | 388,951 | 93,664 | 18 | -8,550 | 491,147 | 420,000 | 86% |
| Algeria | 6,368 | 4,356,206 |  | -20,279 | 4,382,853 | 3,377,671 | 77% |
| Angola | 2,818,684 | 73,713 | 243 | -100,000 | 2,992,154 | 1,023,859 | 34% |
| Argentina | 56,860,704 | 5,141 | 36,075,720 | 100,000 | 20,690,125 | 14,800,000 | 72% |
| Australia | 327,206 | 4,773 | 63,278 | 6,000 | 262,701 | 174,620 | 66% |
| Austria | 2,298,880 | 1,093,958 | 403,190 | -589,882 | 3,579,530 | 1,342,028 | 37% |
| Azerbaijan | 283,570 | 71,069 | 1 | -1,000 | 355,638 | 171,067 | 48% |
| Bahamas | 666 | 1,596 |  | 0 | 2,262 | 844 | 37% |
| Bangladesh | 3,569,321 | 1,290,922 | 2 | -55,173 | 4,915,414 | 4,453,698 | 91% |
| Barbados | 42 | 35,268 | 3 | 0 | 35,307 | 29,700 | 84% |
| Belgium | 429,220 | 1,999,695 | 185,739 | -68,333 | 2,311,509 | 1,190,638 | 52% |
| Belize | 58,049 | 978 | 7,123 | 724 | 51,180 | 36,766 | 72% |
| Benin | 1,580,750 | 2,653 | 2 | -113,630 | 1,697,031 | 474,497 | 28% |
| Bolivia | 987,503 | 6,258 | 6,276 | -100,000 | 1,087,485 | 409,199 | 38% |
| Bosnia and Herzegovina | 1,235,596 | 180,843 | 543 | -5,000 | 1,420,896 | 424,068 | 30% |
| Botswana | 7,278 | 244,879 | 586 | 0 | 251,571 | 29,168 | 12% |
| Brazil | 101,138,617 | 1,459,968 | 42,752,102 | 4,004,000 | 55,842,483 | 47,237,194 | 85% |
| Bulgaria | 4,059,790 | 26,524 | 2,588,956 | -105,647 | 1,603,005 | 160,635 | 10% |
| Cabo Verde | 640 | 28,414 |  | 4,500 | 24,554 | 7,333 | 30% |
| Cambodia | 895,000 | 17,848 | 1,867 | 0 | 910,981 | 571,000 | 63% |
| Cameroon | 2,309,535 | 14,936 | 11 | -171,531 | 2,495,991 | 605,384 | 24% |
| Canada | 13,403,900 | 2,318,466 | 1,217,011 | -579,000 | 15,084,355 | 7,903,474 | 52% |
| Chad | 414,606 | 10,000 |  | 15,000 | 409,606 | 34,153 | 8% |
| Chile | 973,178 | 2,410,905 | 22,587 | -150,000 | 3,511,496 | 2,451,111 | 70% |
| China, mainland | 260,778,900 | 4,791,058 | 25,703 | 9,637,000 | 255,907,255 | 195,489,739 | 76% |
| China, Taiwan Province of | 178,762 | 4,806,245 | 163 | -14,826 | 4,999,670 | 4,538,638 | 91% |
| Colombia | 1,394,863 | 5,992,611 | 1,216 | 29,000 | 7,357,258 | 5,448,347 | 74% |
| Comoros | 7,179 | 36 |  | -364 | 7,579 | 1,256 | 17% |
| Costa Rica | 6,241 | 941,159 | 53 | -26,357 | 973,704 | 739,021 | 76% |
| Côte d'Ivoire | 1,100,000 | 14,318 | 14,076 | -318,164 | 1,418,406 | 133,175 | 9% |
| Afghanistan | 184,671 | 13,922 | 16 | 0 | 198,577 | 118,000 | 59% |

**Table S6.** Continued.

| Country | Production | Import Quantity | Export Quantity | Stock Variation | Domestic supply quantity | Feed use | Share of feed use |
| --- | --- | --- | --- | --- | --- | --- | --- |
| Croatia | 2,298,320 | 42,083 | 882,694 | 4,779 | 1,452,930 | 1,171,162 | 81% |
| Cuba | 275,919 | 873,225 |  | 4,000 | 1,145,144 | 683,195 | 60% |
| Cyprus |  | 313,348 | 26 | 0 | 313,322 | 299,824 | 96% |
| Czechia | 620,260 | 247,143 | 181,407 | -9,966 | 695,962 | 561,800 | 81% |
| Denmark | 40,820 | 436,218 | 82,170 | -42,021 | 436,889 | 323,039 | 74% |
| Dominican Republic | 49,840 | 1,502,510 |  | -31,000 | 1,583,350 | 1,397,478 | 88% |
| Ecuador | 1,479,770 | 42,911 | 5,247 | 21,000 | 1,496,434 | 708,470 | 47% |
| Egypt | 7,593,000 | 8,078,446 | 6,502 | 0 | 15,664,944 | 7,519,817 | 48% |
| El Salvador | 785,991 | 794,581 | 1,385 | 54,000 | 1,525,187 | 1,053,437 | 69% |
| Estonia |  | 25,331 | 62 | -503 | 25,772 | 12,838 | 50% |
| Eswatini | 95,000 | 149,968 | 562 | -44,380 | 288,786 | 44,812 | 16% |
| Ethiopia | 9,635,735 | 25,698 |  | -138,381 | 9,799,814 | 861,313 | 9% |
| Fiji | 1,400 | 126 | 2 | 0 | 1,524 | 840 | 55% |
| France | 12,845,020 | 704,995 | 3,672,345 | -1,354,669 | 11,232,339 | 4,889,788 | 44% |
| French Polynesia |  | 3,601 |  | 0 | 3,601 | 3,601 | 100% |
| Gabon | 47,279 | 412 |  | 713 | 46,978 | 23,776 | 51% |
| Georgia | 207,100 | 88,193 | 770 | -17,000 | 311,523 | 133,000 | 48% |
| Germany | 3,664,800 | 4,562,511 | 336,642 | -126,605 | 8,017,274 | 5,964,767 | 77% |
| Ghana | 2,760,000 | 8,819 | 259 | -108,000 | 2,876,560 | 1,385,766 | 52% |
| Greece | 1,233,620 | 805,728 | 7,214 | -54,405 | 2,086,539 | 1,768,019 | 89% |
| Grenada | 358 | 5,669 |  | 0 | 6,027 | 5,504 | 91% |
| Guatemala | 1,870,000 | 1,323,370 | 1,493 | 94,000 | 3,097,877 | 1,048,676 | 32% |
| Guinea | 871,359 | 1,724 | 1 | 10,000 | 863,082 | 555,326 | 63% |
| Guyana | 4,000 | 32,965 | 16 | -57 | 37,006 | 35,206 | 95% |
| Haiti | 220,000 | 47,581 | 5 | 30,000 | 237,576 | 29,874 | 10% |
| Honduras | 595,479 | 691,370 | 624 | -38,269 | 1,324,494 | 460,000 | 37% |
| Hungary | 8,229,690 | 179,351 | 3,025,729 | 0 | 5,383,312 | 1,617,495 | 30% |
| India | 27,715,100 | 312,389 | 379,469 | -4,241,746 | 31,889,766 | 9,776,814 | 42% |
| Indonesia | 30,693,355 | 1,028,522 | 3,427 | -1,348,106 | 33,066,556 | 2,800,000 | 9% |
| Iran (Islamic Republic of) | 1,400,000 | 7,388,742 | 354 | -384,632 | 9,173,020 | 8,065,033 | 96% |
| Iraq | 473,064 | 822,819 | 58 | -26,000 | 1,321,825 | 1,219,906 | 96% |
| Ireland |  | 1,539,222 | 69,706 | -8,284 | 1,477,800 | 1,388,953 | 95% |

**Table S6.** Continued.

| Country | Production | Import Quantity | Export Quantity | Stock Variation | Domestic supply quantity | Feed use | Share of feed use |
| --- | --- | --- | --- | --- | --- | --- | --- |
| Israel | 77,801 | 1,692,768 | 1,223 | 38,900 | 1,730,446 | 1,644,662 | 91% |
| Italy | 6,279,120 | 6,394,217 | 34,220 | 284,340 | 12,354,777 | 11,515,495 | 89% |
| Jamaica | 2,058 | 283,109 | 206 | 5,000 | 279,961 | 246,055 | 85% |
| Japan | 145 | 15,986,045 | 16 | 0 | 15,986,174 | 12,260,000 | 77% |
| Jordan | 20,936 | 770,599 | 6,777 | -8,700 | 793,458 | 714,477 | 92% |
| Kazakhstan | 895,978 | 7,543 | 86,779 | -369,371 | 1,186,113 | 323,647 | 72% |
| Kuwait | 15,121 | 223,808 |  | -6,234 | 245,163 | 213,112 | 92% |
| Kyrgyzstan | 711,786 | 1,651 | 427 | 9,000 | 704,010 | 522,416 | 72% |
| Latvia |  | 98,050 | 46,549 | -17,830 | 69,331 | 28,582 | 85% |
| Lebanon | 3,000 | 633,921 | 97 | 30,000 | 606,824 | 522,382 | 78% |
| Liberia |  | 1,034 |  | 0 | 1,034 | 1,034 | 100% |
| Libya | 3,613 | 132,095 |  | 33 | 135,675 | 127,172 | 94% |
| Lithuania | 97,970 | 375,857 | 242,020 | -2,296 | 234,103 | 180,085 | 78% |
| Luxembourg | 660 | 16,404 | 10,980 | 0 | 6,084 | 6,000 | 99% |
| Malawi | 3,030,000 | 2,911 | 1,954 | 130,600 | 2,900,357 | 56,479 | 2% |
| Malaysia | 53,773 | 3,755,359 | 6,638 | 495 | 3,801,999 | 3,485,104 | 92% |
| Mali | 3,816,536 | 1,110 | 10,807 | -988,598 | 4,795,437 | 1,428,312 | 51% |
| Malta |  | 45,570 |  | -55 | 45,625 | 42,684 | 94% |
| Mexico | 27,228,242 | 15,472,700 | 644,889 | 439,940 | 41,616,113 | 19,456,000 | 46% |
| Montenegro | 2,810 | 30,896 | 32 | 0 | 33,674 | 28,369 | 84% |
| Morocco | 40,491 | 2,731,200 | 17 | -64,372 | 2,836,046 | 656,639 | 24% |
| Mozambique | 2,085,000 | 171,438 | 288 | 95,000 | 2,161,150 | 162,763 | 7% |
| Myanmar | 1,985,765 | 12,146 | 930,840 | 372,074 | 694,997 | 1,124,138 | 78% |
| Namibia | 45,000 | 97,739 | 67 | 31,595 | 111,077 | 23,120 | 13% |
| Nepal | 2,653,243 | 334,392 |  | 9,002 | 2,978,633 | 1,040,594 | 35% |
| Netherlands | 148,300 | 6,383,334 | 1,050,765 | -413,233 | 5,894,102 | 4,004,476 | 79% |
| New Caledonia | 8,842 | 42 |  | 250 | 8,634 | 8,754 | 96% |
| New Zealand | 195,955 | 245,688 | 3,556 | -8,013 | 446,100 | 296,508 | 69% |
| Nicaragua | 400,146 | 279,085 | 4,374 | -13,827 | 688,684 | 63,551 | 10% |
| Nigeria | 11,000,000 | 200,000 |  | 100,000 | 11,100,000 | 3,430,169 | 30% |
| North Macedonia | 148,058 | 41,171 | 24,420 | 3,000 | 161,809 | 122,948 | 73% |

**Table S6.** Continued.

| Country | Production | Import Quantity | Export Quantity | Stock Variation | Domestic supply quantity | Feed use | Share of feed use |
| --- | --- | --- | --- | --- | --- | --- | --- |
| Norway |  | 61,520 | 162 | 0 | 61,358 | 61,358 | 100% |
| Pakistan | 7,236,313 | 20,190 | 134,681 | 14,000 | 7,107,822 | 1,545,298 | 22% |
| Panama | 123,065 | 514,378 | 1,528 | -34,060 | 669,975 | 476,041 | 79% |
| Paraguay | 5,576,900 | 15,419 | 2,993,286 | 84,376 | 2,514,657 | 1,400,000 | 52% |
| Peru | 1,579,796 | 4,009,801 | 11,381 | 9,000 | 5,569,216 | 4,940,000 | 88% |
| Philippines | 7,978,845 | 458,443 | 417 | 98,064 | 8,338,807 | 5,120,249 | 60% |
| Poland | 3,664,550 | 388,619 | 1,145,731 | 0 | 2,907,438 | 2,590,719 | 89% |
| Portugal | 748,780 | 2,122,805 | 151,776 | -22,611 | 2,742,420 | 2,457,934 | 91% |
| Republic of Korea | 76,000 | 11,366,877 | 89 | -163,000 | 11,605,788 | 8,459,421 | 75% |
| Republic of Moldova | 2,129,889 | 3,796 | 736,986 | 38,700 | 1,357,999 | 1,228,839 | 86% |
| Romania | 17,432,220 | 618,355 | 6,676,219 | -3,205,877 | 14,580,233 | 7,292,479 | 89% |
| Russian Federation | 14,282,352 | 33,181 | 3,119,665 | -453,000 | 11,648,868 | 8,820,533 | 82% |
| Rwanda | 421,218 | 77,137 | 86 | 112,676 | 385,593 | 267,783 | 44% |
| Saudi Arabia | 134,881 | 3,260,945 | 10 | -3,198 | 3,399,014 | 2,288,153 | 67% |
| Serbia | 7,344,542 | 4,151 | 3,132,823 | -49,216 | 4,265,086 | 3,481,722 | 84% |
| Seychelles |  | 4,917 |  | -1,160 | 6,077 | 2,200 | 59% |
| Sierra Leone | 38,685 | 300 |  | 0 | 38,985 | 9,363 | 24% |
| Slovakia | 1,444,810 | 111,343 | 444,523 | -154,067 | 1,265,697 | 416,878 | 44% |
| Slovenia | 360,360 | 703,022 | 763,441 | 40,000 | 259,941 | 120,879 | 36% |
| Solomon Islands |  | 200 |  | 10 | 190 | 137 | 65% |
| South Africa | 11,275,500 | 590,494 | 1,180,768 | 1,716,012 | 8,969,214 | 5,697,000 | 46% |
| Spain | 4,184,460 | 10,012,619 | 152,456 | -592,212 | 14,636,835 | 11,800,000 | 88% |
| Sri Lanka | 245,647 | 110,907 | 2 | 18,543 | 338,009 | 179,716 | 48% |
| Sudan | 25,000 | 21,837 | 13,306 | -12,473 | 46,004 | 2,000 | 9% |
| Suriname | 142 | 7,148 |  | 278 | 7,012 | 6,883 | 91% |
| Sweden | 11,300 | 126,537 | 606 | -50,000 | 187,231 | 1,456 | 2% |
| Switzerland | 174,627 | 139,672 | 2,030 | 11,000 | 301,269 | 306,053 | 95% |
| Syrian Arab Republic | 215,309 | 300,746 | 1 | 4,000 | 512,054 | 412,483 | 79% |
| Tajikistan | 232,899 | 982 |  | -2,441 | 236,322 | 144,161 | 62% |
| Thailand | 4,309,480 | 400,679 | 24,207 | 0 | 4,685,952 | 3,716,421 | 79% |
| Togo | 912,086 | 1,031 | 101 | -5,000 | 918,016 | 138,918 | 15% |

**Table S6.** Continued.

| Country | Production | Import Quantity | Export Quantity | Stock Variation | Domestic supply quantity | Feed use | Share of feed use |
| --- | --- | --- | --- | --- | --- | --- | --- |
| Trinidad and Tobago | 5,000 | 83,059 | 561 | -6,000 | 93,498 | 77,258 | 95% |
| Tunisia |  | 1,025,880 |  | 0 | 1,025,880 | 1,025,880 | 100% |
| Turkey | 6,000,000 | 4,347,475 | 691,567 | -1,078,971 | 10,734,879 | 5,250,604 | 61% |
| Turkmenistan | 40,000 | 2,531 | 25 | 0 | 42,506 | 39,193 | 92% |
| Uganda | 3,588,000 | 790 | 17,511 | -650,000 | 4,221,279 | 148,184 | 5% |
| United Arab Emirates | 26,072 | 452,081 | 43,521 | 500 | 434,132 | 419,313 | 96% |
| United Kingdom |  | 2,781,577 | 171,230 | -355,960 | 2,966,307 | 724,730 | 32% |
| United Republic of Tanzania | 5,652,005 | 74,158 | 279,167 | -143,337 | 5,590,333 | 1,296,901 | 24% |
| United States of America | 347,047,570 | 1,050,349 | 41,562,313 | 7,653,000 | 298,882,606 | 149,863,000 | 48% |
| Uruguay | 816,000 | 242,789 | 55,944 | -81,000 | 1,083,845 | 730,817 | 79% |
| Uzbekistan | 421,273 | 97,391 |  | -6,000 | 524,664 | 276,637 | 54% |
| Vanuatu | 916 | 96 |  | 0 | 1,012 | 904 | 89% |
| Venezuela | 1,566,176 | 1,604,806 |  | 0 | 3,170,982 | 1,723,278 | 54% |
| Viet Nam | 4,756,232 | 11,447,667 | 97,396 | 39,541 | 16,066,962 | 13,334,155 | 83% |
| Yemen | 48,290 | 698,799 | 10,457 | 70,000 | 666,632 | 322,065 | 40% |
| Zambia | 2,004,389 | 2,367 | 36,048 | 854,912 | 1,115,796 | 284,347 | 10% |

Data source: FAO.[FAOSTAT]. License: CC BY-NC-SA 3.0 IGO. Extracted from: [https://www.fao.org/faostat/en/#data]. Data of Access: [3-June-2022].

Table S7. Dependency on Russian fertilizer imports in 2020 (in order of countries’ total types of fertilizer imports).

| **No.** | **Code** | **Importer** | **Resource** | **Import quantity (kt)** | **Import share** | **Herfindahl–Hirschman Index** | **Dependency on Russia** |
| --- | --- | --- | --- | --- | --- | --- | --- |
| 1 | BGD | Bangladesh | Potassic fertilizers | 121 | 0.21 | 0.52 | Low |
| 2 | BDI | Burundi | Nitrogenous fertilizers | 0.1 | 0.27 | 0.18 | Very low |
| 3 | KHM | Cambodia | Mixed fertilizers | 0.1 | 0.00 | 0.40 | Very low |
| 4 | CAF | Central African Republic | Nitrogenous fertilizers | 5 | 1.00 | 1.00 | Very high |
| 5 | HKG | China, Hong Kong SAR | Mixed fertilizers | 545 | 1.00 | 0.99 | Very high |
| 6 | DMA | Dominica | Nitrogenous fertilizers | 0.1 | 0.95 | 0.91 | Very high |
| 7 | GNQ | Equatorial Guinea | Nitrogenous fertilizers | 0.04 | 0.20 | 0.40 | Very low |
| 8 | FRO | Faeroe Islands | Nitrogenous fertilizers | 0.1 | 0.05 | 0.69 | Low |
| 9 | FJI | Fiji | Potassic fertilizers | 0.3 | 0.08 | 0.44 | Very low |
| 10 | GIN | Guinea | Nitrogenous fertilizers | 6 | 0.37 | 0.28 | Very low |
| 11 | GUY | Guyana | Nitrogenous fertilizers | 0.4 | 0.01 | 0.32 | Very low |
| 12 | JAM | Jamaica | Mixed fertilizers | 0.1 | 0.01 | 0.61 | Low |
| 13 | LBY | Libya | Nitrogenous fertilizers | 0.002 | 0.00 | 0.52 | Low |
| 14 | MLI | Mali | Nitrogenous fertilizers | 10 | 0.66 | 0.47 | High |
| 15 | MLT | Malta | Nitrogenous fertilizers | 0.02 | 0.01 | 0.35 | Very low |
| 16 | MRT | Mauritania | Nitrogenous fertilizers | 6 | 0.08 | 0.19 | Very low |
| 17 | NAM | Namibia | Nitrogenous fertilizers | 4 | 0.15 | 0.74 | Low |
| 18 | NER | Niger | Potassic fertilizers | 5 | 1.00 | 1.00 | Very high |
| 19 | PHL | Philippines | Potassic fertilizers | 6 | 0.02 | 0.34 | Very low |
| 20 | SYC | Seychelles | Nitrogenous fertilizers | 0.02 | 0.37 | 0.30 | Very low |
| 21 | SLE | Sierra Leone | Nitrogenous fertilizers | 0.2 | 0.09 | 0.29 | Very low |
| 22 | SGP | Singapore | Mixed fertilizers | 0.000001 | 0.00 | 0.13 | Very low |
| 23 | SUR | Suriname | Nitrogenous fertilizers | 8 | 0.65 | 0.47 | High |
| 24 | YEM | Yemen | Nitrogenous fertilizers | 0.004 | 0.00 | 0.76 | Low |
| 25 | ZWE | Zimbabwe | Nitrogenous fertilizers | 19 | 0.08 | 0.19 | Very low |
| **Subtotal** | | | | **736** |  | | |
| 26 | ALB | Albania | Nitrogenous fertilizers | 50 | 0.74 | 0.56 | Very high |
| 26 | ALB | Albania | Mixed fertilizers | 1 | 0.04 | 0.29 | Very low |
| 27 | BLZ | Belize | Potassic fertilizers | 0.1 | 0.01 | 0.78 | Low |
| 27 | BLZ | Belize | Mixed fertilizers | 0.2 | 0.00 | 0.61 | Low |
| 28 | BEN | Benin | Nitrogenous fertilizers | 66 | 0.93 | 0.87 | Very high |
| 28 | BEN | Benin | Mixed fertilizers | 173 | 0.83 | 0.71 | Very high |

**Table S7.** Continued.

| **No.** | **Code** | **Importer** | **Resource** | **Import quantity (kt)** | **Import share** | **Herfindahl–Hirschman Index** | **Dependency on Russia** |
| --- | --- | --- | --- | --- | --- | --- | --- |
| 29 | COD | Congo, Democratic Republic | Nitrogenous fertilizers | 12 | 0.16 | 0.22 | Very low |
| 29 | COD | Congo, Democratic Republic | Mixed fertilizers | 2 | 0.17 | 0.26 | Very low |
| 30 | HRV | Croatia | Potassic fertilizers | 42 | 0.51 | 0.32 | High |
| 30 | HRV | Croatia | Mixed fertilizers | 4 | 0.02 | 0.21 | Very low |
| 31 | CYP | Cyprus | Nitrogenous fertilizers | 0.002 | 0.00 | 0.18 | Very low |
| 31 | CYP | Cyprus | Mixed fertilizers | 0.00005 | 0.00 | 0.39 | Very low |
| 32 | DNK | Denmark | Nitrogenous fertilizers | 63 | 0.09 | 0.31 | Very low |
| 32 | DNK | Denmark | Mixed fertilizers | 117 | 0.28 | 0.24 | Very low |
| 33 | GAB | Gabon | Nitrogenous fertilizers | 1 | 0.75 | 0.58 | Very high |
| 33 | GAB | Gabon | Mixed fertilizers | 0.3 | 0.02 | 0.83 | Low |
| 34 | ISR | Israel | Nitrogenous fertilizers | 93 | 0.37 | 0.24 | Very low |
| 34 | ISR | Israel | Mixed fertilizers | 5 | 0.16 | 0.19 | Very low |
| 35 | KOR | Korea, Republic | Mixed fertilizers | 0.05 | 0.00 | 0.61 | Low |
| 35 | KOR | Korea, Republic | Potassic fertilizers | 30 | 0.02 | 0.28 | Very low |
| 36 | KWT | Kuwait | Nitrogenous fertilizers | 0.1 | 0.03 | 0.22 | Very low |
| 36 | KWT | Kuwait | Mixed fertilizers | 0.1 | 0.01 | 0.25 | Very low |
| 37 | LBN | Lebanon | Nitrogenous fertilizers | 5 | 0.11 | 0.50 | Very low |
| 37 | LBN | Lebanon | Mixed fertilizers | 0.4 | 0.02 | 0.10 | Very low |
| 38 | LBR | Liberia | Nitrogenous fertilizers | 10 | 0.78 | 0.65 | Very high |
| 38 | LBR | Liberia | Potassic fertilizers | 1 | 0.13 | 0.58 | Low |
| 39 | MNE | Montenegro | Nitrogenous fertilizers | 0.2 | 0.05 | 0.30 | Very low |
| 39 | MNE | Montenegro | Mixed fertilizers | 0.2 | 0.03 | 0.23 | Very low |
| 40 | MMR | Myanmar | Potassic fertilizers | 9 | 0.12 | 0.25 | Very low |
| 40 | MMR | Myanmar | Mixed fertilizers | 30 | 0.03 | 0.25 | Very low |
| 41 | NZL | New Zealand | Nitrogenous fertilizers | 0.2 | 0.00 | 0.30 | Very low |
| 41 | NZL | New Zealand | Potassic fertilizers | 0.3 | 0.00 | 0.39 | Very low |
| 42 | NGA | Nigeria | Potassic fertilizers | 157 | 0.76 | 0.61 | Very high |
| 42 | NGA | Nigeria | Nitrogenous fertilizers | 18 | 0.20 | 0.66 | Low |
| 43 | OMN | Oman | Potassic fertilizers | 16 | 0.86 | 0.75 | Very high |
| 43 | OMN | Oman | Nitrogenous fertilizers | 3 | 0.24 | 0.13 | Very low |
| 44 | SAU | Saudi Arabia | Nitrogenous fertilizers | 3 | 0.03 | 0.36 | Very low |

**Table S7.** Continued.

| **No.** | **Code** | **Importer** | **Resource** | **Import quantity (kt)** | **Import share** | **Herfindahl–Hirschman Index** | **Dependency on Russia** |
| --- | --- | --- | --- | --- | --- | --- | --- |
| 44 | SAU | Saudi Arabia | Mixed fertilizers | 1 | 0.01 | 0.16 | Very low |
| 45 | LKA | Sri Lanka | Potassic fertilizers | 21 | 0.09 | 0.40 | Very low |
| 45 | LKA | Sri Lanka | Mixed fertilizers | 1 | 0.04 | 0.26 | Very low |
| 46 | TJK | Tajikistan | Nitrogenous fertilizers | 1 | 0.01 | 0.73 | Low |
| 46 | TJK | Tajikistan | Mixed fertilizers | 14 | 0.28 | 0.31 | Very low |
| 47 | TGO | Togo | Nitrogenous fertilizers | 13 | 0.21 | 0.26 | Very low |
| 47 | TGO | Togo | Mixed fertilizers | 25 | 0.17 | 0.35 | Very low |
| 48 | TUN | Tunisia | Nitrogenous fertilizers | 0.4 | 0.01 | 0.31 | Very low |
| 48 | TUN | Tunisia | Mixed fertilizers | 1 | 0.07 | 0.26 | Very low |
| 49 | ZMB | Zambia | Nitrogenous fertilizers | 5 | 0.01 | 0.19 | Very low |
| 49 | ZMB | Zambia | Mixed fertilizers | 2 | 0.01 | 0.13 | Very low |
| **Subtotal** | | | | **999** |  | | |
| 50 | DZA | Algeria | Nitrogenous fertilizers | 10 | 0.14 | 0.18 | Very low |
| 50 | DZA | Algeria | Potassic fertilizers | 0.2 | 0.02 | 0.33 | Very low |
| 50 | DZA | Algeria | Mixed fertilizers | 10 | 0.06 | 0.24 | Very low |
| 51 | AGO | Angola | Nitrogenous fertilizers | 9 | 0.14 | 0.50 | Low |
| 51 | AGO | Angola | Potassic fertilizers | 1 | 0.10 | 0.28 | Very low |
| 51 | AGO | Angola | Mixed fertilizers | 3 | 0.07 | 0.28 | Very low |
| 52 | ARG | Argentina | Nitrogenous fertilizers | 246 | 0.13 | 0.18 | Very low |
| 52 | ARG | Argentina | Potassic fertilizers | 19 | 0.23 | 0.19 | Very low |
| 52 | ARG | Argentina | Mixed fertilizers | 64 | 0.04 | 0.24 | Very low |
| 53 | ARM | Armenia | Mixed fertilizers | 4 | 0.78 | 0.62 | Very high |
| 53 | ARM | Armenia | Nitrogenous fertilizers | 33 | 0.45 | 0.35 | Very low |
| 53 | ARM | Armenia | Potassic fertilizers | 0.4 | 0.44 | 0.27 | Very low |
| 54 | AUS | Australia | Nitrogenous fertilizers | 255 | 0.08 | 0.12 | Very low |
| 54 | AUS | Australia | Potassic fertilizers | 0.2 | 0.00 | 0.24 | Very low |
| 54 | AUS | Australia | Mixed fertilizers | 2 | 0.00 | 0.42 | Very low |
| 55 | AUT | Austria | Potassic fertilizers | 0.1 | 0.00 | 0.78 | Low |
| 55 | AUT | Austria | Nitrogenous fertilizers | 0.5 | 0.00 | 0.19 | Very low |
| 55 | AUT | Austria | Mixed fertilizers | 0.3 | 0.00 | 0.15 | Very low |
| 56 | AZE | Azerbaijan | Nitrogenous fertilizers | 168 | 0.81 | 0.67 | Very high |
| 56 | AZE | Azerbaijan | Mixed fertilizers | 124 | 0.95 | 0.90 | Very high |

**Table S7.** Continued.

| **No.** | **Code** | **Importer** | **Resource** | **Import quantity (kt)** | **Import share** | **Herfindahl–Hirschman Index** | **Dependency on Russia** |
| --- | --- | --- | --- | --- | --- | --- | --- |
| 56 | AZE | Azerbaijan | Potassic fertilizers | 3 | 0.32 | 0.21 | Very low |
| 57 | BLR | Belarus | Nitrogenous fertilizers | 137 | 0.90 | 0.81 | Very high |
| 57 | BLR | Belarus | Potassic fertilizers | 2 | 0.74 | 0.57 | Very high |
| 57 | BLR | Belarus | Mixed fertilizers | 193 | 0.98 | 0.96 | Very high |
| 58 | BEL | Belgium | Nitrogenous fertilizers | 30 | 0.02 | 0.41 | Very low |
| 58 | BEL | Belgium | Potassic fertilizers | 195 | 0.14 | 0.21 | Very low |
| 58 | BEL | Belgium | Mixed fertilizers | 83 | 0.21 | 0.18 | Very low |
| 59 | BOL | Bolivia | Nitrogenous fertilizers | 14 | 0.28 | 0.28 | Very low |
| 59 | BOL | Bolivia | Potassic fertilizers | 0.5 | 0.07 | 0.23 | Very low |
| 59 | BOL | Bolivia | Mixed fertilizers | 4 | 0.05 | 0.22 | Very low |
| 60 | BIH | Bosnia Herzegovina | Nitrogenous fertilizers | 1 | 0.01 | 0.55 | Low |
| 60 | BIH | Bosnia Herzegovina | Potassic fertilizers | 2 | 0.32 | 0.21 | Very low |
| 60 | BIH | Bosnia Herzegovina | Mixed fertilizers | 12 | 0.17 | 0.32 | Very low |
| 61 | BRA | Brazil | Nitrogenous fertilizers | 2,673 | 0.21 | 0.13 | Very low |
| 61 | BRA | Brazil | Potassic fertilizers | 2,939 | 0.25 | 0.21 | Very low |
| 61 | BRA | Brazil | Mixed fertilizers | 1,720 | 0.20 | 0.20 | Very low |
| 62 | BGR | Bulgaria | Nitrogenous fertilizers | 46 | 0.10 | 0.09 | Very low |
| 62 | BGR | Bulgaria | Potassic fertilizers | 3 | 0.19 | 0.15 | Very low |
| 62 | BGR | Bulgaria | Mixed fertilizers | 41 | 0.12 | 0.14 | Very low |
| 63 | BFA | Burkina Faso | Nitrogenous fertilizers | 52 | 0.58 | 0.37 | High |
| 63 | BFA | Burkina Faso | Potassic fertilizers | 3 | 0.42 | 0.30 | Very low |
| 63 | BFA | Burkina Faso | Mixed fertilizers | 1 | 0.01 | 0.33 | Very low |
| 64 | CMR | Cameroon | Potassic fertilizers | 0.4 | 0.01 | 0.94 | Low |
| 64 | CMR | Cameroon | Nitrogenous fertilizers | 40 | 0.48 | 0.30 | Very low |
| 64 | CMR | Cameroon | Mixed fertilizers | 18 | 0.33 | 0.29 | Very low |
| 65 | CAN | Canada | Potassic fertilizers | 14 | 0.12 | 0.65 | Low |
| 65 | CAN | Canada | Mixed fertilizers | 216 | 0.08 | 0.72 | Low |
| 65 | CAN | Canada | Nitrogenous fertilizers | 345 | 0.18 | 0.29 | Very low |
| 66 | CHL | Chile | Nitrogenous fertilizers | 52 | 0.07 | 0.24 | Very low |
| 66 | CHL | Chile | Potassic fertilizers | 1 | 0.01 | 0.24 | Very low |
| 66 | CHL | Chile | Mixed fertilizers | 0.4 | 0.00 | 0.48 | Very low |
| 67 | CHN | China | Mixed fertilizers | 619 | 0.82 | 0.20 | High |

**Table S7.** Continued.

| **No.** | **Code** | **Importer** | **Resource** | **Import quantity (kt)** | **Import share** | **Herfindahl–Hirschman Index** | **Dependency on Russia** |
| --- | --- | --- | --- | --- | --- | --- | --- |
| 67 | CHN | China | Nitrogenous fertilizers | 1 | 0.03 | 0.20 | Very low |
| 67 | CHN | China | Potassic fertilizers | 2,105 | 0.23 | 0.21 | Very low |
| 68 | COL | Colombia | Nitrogenous fertilizers | 226 | 0.23 | 0.15 | Very low |
| 68 | COL | Colombia | Potassic fertilizers | 95 | 0.13 | 0.17 | Very low |
| 68 | COL | Colombia | Mixed fertilizers | 27 | 0.06 | 0.34 | Very low |
| 69 | COG | Congo, Republic | Nitrogenous fertilizers | 1 | 0.42 | 0.26 | Very low |
| 69 | COG | Congo, Republic | Potassic fertilizers | 0.3 | 0.18 | 0.18 | Very low |
| 69 | COG | Congo, Republic | Mixed fertilizers | 1 | 0.17 | 0.18 | Very low |
| 70 | CRI | Costa Rica | Nitrogenous fertilizers | 94 | 0.44 | 0.26 | Very low |
| 70 | CRI | Costa Rica | Potassic fertilizers | 28 | 0.23 | 0.21 | Very low |
| 70 | CRI | Costa Rica | Mixed fertilizers | 6 | 0.04 | 0.11 | Very low |
| 71 | CIV | Cote d'Ivoire | Nitrogenous fertilizers | 91 | 0.68 | 0.49 | High |
| 71 | CIV | Cote d'Ivoire | Potassic fertilizers | 4 | 0.04 | 0.84 | Low |
| 71 | CIV | Cote d'Ivoire | Mixed fertilizers | 53 | 0.30 | 0.53 | Low |
| 72 | CZE | Czech Republic | Potassic fertilizers | 4 | 0.03 | 0.54 | Low |
| 72 | CZE | Czech Republic | Nitrogenous fertilizers | 26 | 0.03 | 0.18 | Very low |
| 72 | CZE | Czech Republic | Mixed fertilizers | 13 | 0.07 | 0.29 | Very low |
| 73 | DOM | Dominican Republic | Nitrogenous fertilizers | 21 | 0.11 | 0.34 | Very low |
| 73 | DOM | Dominican Republic | Potassic fertilizers | 8 | 0.20 | 0.14 | Very low |
| 73 | DOM | Dominican Republic | Mixed fertilizers | 8 | 0.09 | 0.30 | Very low |
| 74 | ECU | Ecuador | Nitrogenous fertilizers | 281 | 0.51 | 0.34 | High |
| 74 | ECU | Ecuador | Potassic fertilizers | 13 | 0.05 | 0.16 | Very low |
| 74 | ECU | Ecuador | Mixed fertilizers | 67 | 0.36 | 0.18 | Very low |
| 75 | EGY | Egypt | Nitrogenous fertilizers | 0.3 | 0.00 | 0.17 | Very low |
| 75 | EGY | Egypt | Potassic fertilizers | 4 | 0.03 | 0.47 | Very low |
| 75 | EGY | Egypt | Mixed fertilizers | 2 | 0.02 | 0.16 | Very low |
| 76 | SLV | El Salvador | Potassic fertilizers | 17 | 0.56 | 0.40 | High |
| 76 | SLV | El Salvador | Nitrogenous fertilizers | 53 | 0.21 | 0.55 | Low |
| 76 | SLV | El Salvador | Mixed fertilizers | 0.2 | 0.00 | 0.28 | Very low |
| 77 | EST | Estonia | Mixed fertilizers | 591 | 0.89 | 0.79 | Very high |
| 77 | EST | Estonia | Potassic fertilizers | 462 | 0.99 | 0.98 | Very high |
| 77 | EST | Estonia | Nitrogenous fertilizers | 469 | 0.76 | 0.58 | Very high |

**Table S7.** Continued.

| **No.** | **Code** | **Importer** | **Resource** | **Import quantity (kt)** | **Import share** | **Herfindahl–Hirschman Index** | **Dependency on Russia** |
| --- | --- | --- | --- | --- | --- | --- | --- |
| 78 | FIN | Finland | Mixed fertilizers | 143 | 0.74 | 0.57 | Very high |
| 78 | FIN | Finland | Potassic fertilizers | 387 | 0.89 | 0.81 | Very high |
| 78 | FIN | Finland | Nitrogenous fertilizers | 406 | 0.78 | 0.62 | Very high |
| 79 | FRA | France | Mixed fertilizers | 75 | 0.05 | 0.22 | Very low |
| 79 | FRA | France | Potassic fertilizers | 24 | 0.03 | 0.21 | Very low |
| 79 | FRA | France | Nitrogenous fertilizers | 75 | 0.01 | 0.12 | Very low |
| 80 | GEO | Georgia | Mixed fertilizers | 23 | 0.72 | 0.52 | Very high |
| 80 | GEO | Georgia | Nitrogenous fertilizers | 9 | 0.56 | 0.35 | High |
| 80 | GEO | Georgia | Potassic fertilizers | 0.1 | 0.05 | 0.15 | Very low |
| 81 | DEU | Germany | Mixed fertilizers | 90 | 0.00 | 0.14 | Very low |
| 81 | DEU | Germany | Potassic fertilizers | 1 | 0.00 | 0.29 | Very low |
| 81 | DEU | Germany | Nitrogenous fertilizers | 88 | 0.03 | 0.12 | Very low |
| 82 | GHA | Ghana | Nitrogenous fertilizers | 135 | 0.57 | 0.36 | High |
| 82 | GHA | Ghana | Mixed fertilizers | 74 | 0.25 | 0.19 | Very low |
| 82 | GHA | Ghana | Potassic fertilizers | 13 | 0.24 | 0.23 | Very low |
| 83 | GRC | Greece | Mixed fertilizers | 47 | 0.18 | 0.11 | Very low |
| 83 | GRC | Greece | Potassic fertilizers | 6 | 0.06 | 0.33 | Very low |
| 83 | GRC | Greece | Nitrogenous fertilizers | 15 | 0.04 | 0.17 | Very low |
| 84 | GTM | Guatemala | Mixed fertilizers | 11 | 0.03 | 0.18 | Very low |
| 84 | GTM | Guatemala | Potassic fertilizers | 8 | 0.03 | 0.24 | Very low |
| 84 | GTM | Guatemala | Nitrogenous fertilizers | 143 | 0.25 | 0.22 | Very low |
| 85 | HND | Honduras | Nitrogenous fertilizers | 157 | 0.67 | 0.48 | High |
| 85 | HND | Honduras | Mixed fertilizers | 1 | 0.01 | 0.59 | Low |
| 85 | HND | Honduras | Potassic fertilizers | 21 | 0.18 | 0.25 | Very low |
| 86 | HUN | Hungary | Mixed fertilizers | 41 | 0.09 | 0.09 | Very low |
| 86 | HUN | Hungary | Potassic fertilizers | 30 | 0.23 | 0.23 | Very low |
| 86 | HUN | Hungary | Nitrogenous fertilizers | 10 | 0.01 | 0.20 | Very low |
| 87 | IND | India | Mixed fertilizers | 1,238 | 0.15 | 0.20 | Very low |
| 87 | IND | India | Potassic fertilizers | 815 | 0.15 | 0.19 | Very low |
| 87 | IND | India | Nitrogenous fertilizers | 277 | 0.03 | 0.18 | Very low |
| 88 | IDN | Indonesia | Nitrogenous fertilizers | 3 | 0.00 | 0.79 | Low |
| 89 | IRL | Ireland | Mixed fertilizers | 241 | 0.39 | 0.22 | Very low |

**Table S7.** Continued.

| **No.** | **Code** | **Importer** | **Resource** | **Import quantity (kt)** | **Import share** | **Herfindahl–Hirschman Index** | **Dependency on Russia** |
| --- | --- | --- | --- | --- | --- | --- | --- |
| 89 | IRL | Ireland | Nitrogenous fertilizers | 81 | 0.08 | 0.12 | Very low |
| 89 | IRL | Ireland | Potassic fertilizers | 21 | 0.11 | 0.41 | Very low |
| 89 | IRL | Ireland | Nitrogenous fertilizers | 81 | 0.08 | 0.12 | Very low |
| 90 | ITA | Italy | Mixed fertilizers | 67 | 0.10 | 0.18 | Very low |
| 90 | ITA | Italy | Potassic fertilizers | 46 | 0.11 | 0.16 | Very low |
| 90 | ITA | Italy | Nitrogenous fertilizers | 34 | 0.03 | 0.12 | Very low |
| 91 | JPN | Japan | Mixed fertilizers | 3 | 0.01 | 0.65 | Low |
| 91 | JPN | Japan | Potassic fertilizers | 51 | 0.08 | 0.24 | Very low |
| 91 | JPN | Japan | Nitrogenous fertilizers | 2 | 0.00 | 0.27 | Very low |
| 92 | JOR | Jordan | Mixed fertilizers | 0.3 | 0.01 | 0.14 | Very low |
| 92 | JOR | Jordan | Potassic fertilizers | 0.4 | 0.06 | 0.18 | Very low |
| 92 | JOR | Jordan | Nitrogenous fertilizers | 0.1 | 0.00 | 0.33 | Very low |
| 93 | KAZ | Kazakhstan | Mixed fertilizers | 155 | 0.98 | 0.95 | Very high |
| 93 | KAZ | Kazakhstan | Nitrogenous fertilizers | 370 | 0.82 | 0.70 | Very high |
| 93 | KAZ | Kazakhstan | Potassic fertilizers | 9 | 0.33 | 0.39 | Very low |
| 94 | KEN | Kenya | Mixed fertilizers | 119 | 0.25 | 0.27 | Very low |
| 94 | KEN | Kenya | Potassic fertilizers | 2 | 0.06 | 0.16 | Very low |
| 94 | KEN | Kenya | Nitrogenous fertilizers | 0.3 | 0.00 | 0.23 | Very low |
| 95 | KGZ | Kyrgyzstan | Potassic fertilizers | 0.5 | 1.00 | 1.00 | Very high |
| 95 | KGZ | Kyrgyzstan | Nitrogenous fertilizers | 62 | 0.55 | 0.39 | High |
| 95 | KGZ | Kyrgyzstan | Mixed fertilizers | 15 | 0.32 | 0.37 | Very low |
| 96 | LVA | Latvia | Mixed fertilizers | 284 | 0.81 | 0.66 | Very high |
| 96 | LVA | Latvia | Potassic fertilizers | 26 | 0.66 | 0.50 | High |
| 96 | LVA | Latvia | Nitrogenous fertilizers | 225 | 0.37 | 0.21 | Very low |
| 97 | LTU | Lithuania | Potassic fertilizers | 65 | 0.69 | 0.52 | Very high |
| 97 | LTU | Lithuania | Mixed fertilizers | 236 | 0.33 | 0.27 | Very low |
| 97 | LTU | Lithuania | Nitrogenous fertilizers | 220 | 0.27 | 0.12 | Very low |
| 98 | MWI | Malawi | Nitrogenous fertilizers | 1 | 0.00 | 0.51 | Low |
| 98 | MWI | Malawi | Mixed fertilizers | 0.1 | 0.00 | 0.33 | Very low |
| 98 | MWI | Malawi | Potassic fertilizers | 1 | 0.05 | 0.28 | Very low |
| 99 | MYS | Malaysia | Mixed fertilizers | 11 | 0.02 | 0.11 | Very low |
| 99 | MYS | Malaysia | Potassic fertilizers | 182 | 0.12 | 0.21 | Very low |

**Table S7.** Continued.

| **No.** | **Code** | **Importer** | **Resource** | **Import quantity (kt)** | **Import share** | **Herfindahl–Hirschman Index** | **Dependency on Russia** |
| --- | --- | --- | --- | --- | --- | --- | --- |
| 99 | MYS | Malaysia | Nitrogenous fertilizers | 12 | 0.01 | 0.30 | Very low |
| 100 | MUS | Mauritius | Mixed fertilizers | 0.1 | 0.01 | 0.26 | Very low |
| 100 | MUS | Mauritius | Potassic fertilizers | 0.02 | 0.00 | 0.36 | Very low |
| 100 | MUS | Mauritius | Nitrogenous fertilizers | 0.1 | 0.00 | 0.42 | Very low |
| 101 | MEX | Mexico | Mixed fertilizers | 358 | 0.32 | 0.23 | Very low |
| 101 | MEX | Mexico | Potassic fertilizers | 53 | 0.12 | 0.18 | Very low |
| 101 | MEX | Mexico | Nitrogenous fertilizers | 489 | 0.23 | 0.16 | Very low |
| 102 | MDA | Moldova | Mixed fertilizers | 78 | 0.76 | 0.59 | Very high |
| 102 | MDA | Moldova | Nitrogenous fertilizers | 137 | 0.76 | 0.59 | Very high |
| 102 | MDA | Moldova | Potassic fertilizers | 0.3 | 0.32 | 0.20 | Very low |
| 103 | MNG | Mongolia | Nitrogenous fertilizers | 122 | 0.99 | 0.98 | Very high |
| 103 | MNG | Mongolia | Mixed fertilizers | 1 | 0.17 | 0.71 | Low |
| 103 | MNG | Mongolia | Potassic fertilizers | 0.01 | 0.13 | 0.46 | Very low |
| 104 | MAR | Morocco | Potassic fertilizers | 0.4 | 0.00 | 0.67 | Low |
| 104 | MAR | Morocco | Mixed fertilizers | 6 | 0.15 | 0.20 | Very low |
| 104 | MAR | Morocco | Nitrogenous fertilizers | 196 | 0.32 | 0.15 | Very low |
| 105 | MOZ | Mozambique | Mixed fertilizers | 3 | 0.01 | 0.29 | Very low |
| 105 | MOZ | Mozambique | Potassic fertilizers | 8 | 0.30 | 0.18 | Very low |
| 105 | MOZ | Mozambique | Nitrogenous fertilizers | 39 | 0.15 | 0.12 | Very low |
| 106 | NLD | Netherlands | Mixed fertilizers | 11 | 0.03 | 0.28 | Very low |
| 106 | NLD | Netherlands | Potassic fertilizers | 9 | 0.01 | 0.18 | Very low |
| 106 | NLD | Netherlands | Nitrogenous fertilizers | 16 | 0.01 | 0.20 | Very low |
| 107 | NIC | Nicaragua | Nitrogenous fertilizers | 80 | 0.55 | 0.40 | High |
| 107 | NIC | Nicaragua | Mixed fertilizers | 1 | 0.01 | 0.23 | Very low |
| 107 | NIC | Nicaragua | Potassic fertilizers | 7 | 0.09 | 0.32 | Very low |
| 108 | MKD | North Macedonia | Mixed fertilizers | 16 | 0.37 | 0.25 | Very low |
| 108 | MKD | North Macedonia | Potassic fertilizers | 0.3 | 0.27 | 0.18 | Very low |
| 108 | MKD | North Macedonia | Nitrogenous fertilizers | 6 | 0.09 | 0.26 | Very low |
| 109 | NOR | Norway | Potassic fertilizers | 18 | 0.04 | 0.61 | Low |
| 109 | NOR | Norway | Mixed fertilizers | 26 | 0.30 | 0.24 | Very low |
| 109 | NOR | Norway | Nitrogenous fertilizers | 8 | 0.02 | 0.23 | Very low |
| 110 | PAK | Pakistan | Nitrogenous fertilizers | 3 | 0.02 | 0.66 | Low |

**Table S7.** Continued.

| **No.** | **Code** | **Importer** | **Resource** | **Import quantity (kt)** | **Import share** | **Herfindahl–Hirschman Index** | **Dependency on Russia** |
| --- | --- | --- | --- | --- | --- | --- | --- |
| 110 | PAK | Pakistan | Mixed fertilizers | 7 | 0.01 | 0.41 | Very low |
| 110 | PAK | Pakistan | Potassic fertilizers | 3 | 0.02 | 0.15 | Very low |
| 111 | PAN | Panama | Nitrogenous fertilizers | 23 | 0.63 | 0.46 | High |
| 111 | PAN | Panama | Mixed fertilizers | 16 | 0.27 | 0.36 | Very low |
| 111 | PAN | Panama | Potassic fertilizers | 4 | 0.19 | 0.20 | Very low |
| 112 | PRY | Paraguay | Potassic fertilizers | 176 | 0.61 | 0.46 | High |
| 112 | PRY | Paraguay | Mixed fertilizers | 40 | 0.07 | 0.25 | Very low |
| 112 | PRY | Paraguay | Nitrogenous fertilizers | 28 | 0.14 | 0.30 | Very low |
| 113 | PER | Peru | Nitrogenous fertilizers | 512 | 0.53 | 0.32 | High |
| 113 | PER | Peru | Mixed fertilizers | 194 | 0.37 | 0.27 | Very low |
| 113 | PER | Peru | Potassic fertilizers | 44 | 0.16 | 0.19 | Very low |
| 114 | POL | Poland | Mixed fertilizers | 430 | 0.40 | 0.21 | Very low |
| 114 | POL | Poland | Potassic fertilizers | 279 | 0.26 | 0.23 | Very low |
| 114 | POL | Poland | Nitrogenous fertilizers | 174 | 0.11 | 0.15 | Very low |
| 115 | PRT | Portugal | Mixed fertilizers | 1 | 0.00 | 0.51 | Low |
| 115 | PRT | Portugal | Potassic fertilizers | 16 | 0.17 | 0.20 | Very low |
| 115 | PRT | Portugal | Nitrogenous fertilizers | 12 | 0.04 | 0.24 | Very low |
| 116 | ROU | Romania | Mixed fertilizers | 330 | 0.35 | 0.18 | Very low |
| 116 | ROU | Romania | Potassic fertilizers | 32 | 0.26 | 0.44 | Very low |
| 116 | ROU | Romania | Nitrogenous fertilizers | 131 | 0.11 | 0.11 | Very low |
| 117 | SEN | Senegal | Mixed fertilizers | 2 | 0.03 | 0.72 | Low |
| 117 | SEN | Senegal | Potassic fertilizers | 18 | 0.32 | 0.46 | Very low |
| 117 | SEN | Senegal | Nitrogenous fertilizers | 54 | 0.35 | 0.22 | Very low |
| 118 | SRB | Serbia | Mixed fertilizers | 223 | 0.77 | 0.61 | Very high |
| 118 | SRB | Serbia | Potassic fertilizers | 54 | 0.55 | 0.35 | High |
| 118 | SRB | Serbia | Nitrogenous fertilizers | 220 | 0.34 | 0.19 | Very low |
| 119 | SVK | Slovakia | Mixed fertilizers | 21 | 0.14 | 0.14 | Very low |
| 119 | SVK | Slovakia | Potassic fertilizers | 1 | 0.06 | 0.42 | Very low |
| 119 | SVK | Slovakia | Nitrogenous fertilizers | 1 | 0.00 | 0.16 | Very low |
| 120 | SVN | Slovenia | Mixed fertilizers | 65 | 0.41 | 0.26 | Very low |
| 120 | SVN | Slovenia | Potassic fertilizers | 0.02 | 0.01 | 0.28 | Very low |
| 120 | SVN | Slovenia | Nitrogenous fertilizers | 5 | 0.04 | 0.33 | Very low |
| 121 | ZAF | South Africa | Mixed fertilizers | 93 | 0.38 | 0.30 | Very low |

**Table S7.** Continued.

| **No.** | **Code** | **Importer** | **Resource** | **Import quantity (kt)** | **Import share** | **Herfindahl–Hirschman Index** | **Dependency on Russia** |
| --- | --- | --- | --- | --- | --- | --- | --- |
| 121 | ZAF | South Africa | Potassic fertilizers | 58 | 0.11 | 0.15 | Very low |
| 121 | ZAF | South Africa | Nitrogenous fertilizers | 43 | 0.03 | 0.17 | Very low |
| 122 | ESP | Spain | Mixed fertilizers | 117 | 0.08 | 0.18 | Very low |
| 122 | ESP | Spain | Potassic fertilizers | 14 | 0.04 | 0.15 | Very low |
| 122 | ESP | Spain | Nitrogenous fertilizers | 53 | 0.03 | 0.08 | Very low |
| 123 | SWE | Sweden | Mixed fertilizers | 106 | 0.25 | 0.20 | Very low |
| 123 | SWE | Sweden | Potassic fertilizers | 17 | 0.11 | 0.37 | Very low |
| 123 | SWE | Sweden | Nitrogenous fertilizers | 50 | 0.06 | 0.22 | Very low |
| 124 | CHE | Switzerland | Nitrogenous fertilizers | 241 | 0.55 | 0.38 | High |
| 124 | CHE | Switzerland | Mixed fertilizers | 7 | 0.09 | 0.30 | Very low |
| 124 | CHE | Switzerland | Potassic fertilizers | 1 | 0.06 | 0.37 | Very low |
| 125 | TZA | Tanzania | Potassic fertilizers | 2 | 0.50 | 0.39 | High |
| 125 | TZA | Tanzania | Mixed fertilizers | 13 | 0.08 | 0.44 | Very low |
| 125 | TZA | Tanzania | Nitrogenous fertilizers | 22 | 0.06 | 0.20 | Very low |
| 126 | THA | Thailand | Mixed fertilizers | 378 | 0.23 | 0.22 | Very low |
| 126 | THA | Thailand | Potassic fertilizers | 10 | 0.01 | 0.21 | Very low |
| 126 | THA | Thailand | Nitrogenous fertilizers | 11 | 0.00 | 0.23 | Very low |
| 127 | TUR | Turkey | Mixed fertilizers | 173 | 0.17 | 0.31 | Very low |
| 127 | TUR | Turkey | Potassic fertilizers | 46 | 0.16 | 0.48 | Very low |
| 127 | TUR | Turkey | Nitrogenous fertilizers | 53 | 0.02 | 0.16 | Very low |
| 128 | TKM | Turkmenistan | Mixed fertilizers | 24 | 0.85 | 0.73 | Very high |
| 128 | TKM | Turkmenistan | Potassic fertilizers | 0.002 | 0.00 | 0.58 | Low |
| 128 | TKM | Turkmenistan | Nitrogenous fertilizers | 0.1 | 0.00 | 0.95 | Low |
| 129 | UGA | Uganda | Mixed fertilizers | 11 | 0.12 | 0.19 | Very low |
| 129 | UGA | Uganda | Potassic fertilizers | 2 | 0.20 | 0.17 | Very low |
| 129 | UGA | Uganda | Nitrogenous fertilizers | 0.3 | 0.03 | 0.32 | Very low |
| 130 | UKR | Ukraine | Potassic fertilizers | 0.2 | 0.00 | 0.72 | Low |
| 130 | UKR | Ukraine | Mixed fertilizers | 17 | 0.01 | 0.17 | Very low |
| 130 | UKR | Ukraine | Nitrogenous fertilizers | 49 | 0.06 | 0.18 | Very low |
| 131 | ARE | United Arab Emirates | Mixed fertilizers | 34 | 0.54 | 0.33 | High |
| 131 | ARE | United Arab Emirates | Potassic fertilizers | 6 | 0.15 | 0.20 | Very low |
| 131 | ARE | United Arab Emirates | Nitrogenous fertilizers | 1 | 0.01 | 0.21 | Very low |
| 132 | GBR | United Kingdom | Mixed fertilizers | 79 | 0.11 | 0.22 | Very low |

**Table S7.** Continued.

| **No.** | **Code** | **Importer** | **Resource** | **Import quantity (kt)** | **Import share** | **Herfindahl–Hirschman Index** | **Dependency on Russia** |
| --- | --- | --- | --- | --- | --- | --- | --- |
| 132 | GBR | United Kingdom | Potassic fertilizers | 20 | 0.04 | 0.25 | Very low |
| 132 | GBR | United Kingdom | Nitrogenous fertilizers | 168 | 0.07 | 0.12 | Very low |
| 133 | USA | United States | Potassic fertilizers | 803 | 0.07 | 0.74 | Low |
| 133 | USA | United States | Mixed fertilizers | 320 | 0.14 | 0.18 | Very low |
| 133 | USA | United States | Nitrogenous fertilizers | 1,836 | 0.21 | 0.13 | Very low |
| 134 | URY | Uruguay | Mixed fertilizers | 11 | 0.04 | 0.21 | Very low |
| 134 | URY | Uruguay | Potassic fertilizers | 100 | 0.37 | 0.29 | Very low |
| 134 | URY | Uruguay | Nitrogenous fertilizers | 56 | 0.11 | 0.18 | Very low |
| 135 | UZB | Uzbekistan | Mixed fertilizers | 30 | 0.16 | 0.68 | Low |
| 135 | UZB | Uzbekistan | Potassic fertilizers | 0.4 | 0.21 | 0.24 | Very low |
| 135 | UZB | Uzbekistan | Nitrogenous fertilizers | 7 | 0.06 | 0.41 | Very low |
| 136 | VNM | Vietnam | Nitrogenous fertilizers | 12 | 0.01 | 0.50 | Low |
| 136 | VNM | Vietnam | Mixed fertilizers | 127 | 0.10 | 0.41 | Very low |
| 136 | VNM | Vietnam | Potassic fertilizers | 200 | 0.18 | 0.17 | Very low |
| 131 | ARE | United Arab Emirates | Nitrogenous fertilizers | 1 | 0.01 | 0.21 | Very low |
| 132 | GBR | United Kingdom | Mixed fertilizers | 79 | 0.11 | 0.22 | Very low |
| 132 | GBR | United Kingdom | Potassic fertilizers | 20 | 0.04 | 0.25 | Very low |
| 132 | GBR | United Kingdom | Nitrogenous fertilizers | 168 | 0.07 | 0.12 | Very low |
| 133 | USA | United States | Potassic fertilizers | 803 | 0.07 | 0.74 | Low |
| 133 | USA | United States | Mixed fertilizers | 320 | 0.14 | 0.18 | Very low |
| 133 | USA | United States | Nitrogenous fertilizers | 1,836 | 0.21 | 0.13 | Very low |
| 134 | URY | Uruguay | Mixed fertilizers | 11 | 0.04 | 0.21 | Very low |
| 134 | URY | Uruguay | Potassic fertilizers | 100 | 0.37 | 0.29 | Very low |
| 134 | URY | Uruguay | Nitrogenous fertilizers | 56 | 0.11 | 0.18 | Very low |
| 135 | UZB | Uzbekistan | Mixed fertilizers | 30 | 0.16 | 0.68 | Low |
| 135 | UZB | Uzbekistan | Potassic fertilizers | 0.4 | 0.21 | 0.24 | Very low |
| 135 | UZB | Uzbekistan | Nitrogenous fertilizers | 7 | 0.06 | 0.41 | Very low |
| 136 | VNM | Vietnam | Nitrogenous fertilizers | 12 | 0.01 | 0.50 | Low |
| 136 | VNM | Vietnam | Mixed fertilizers | 127 | 0.10 | 0.41 | Very low |
| 136 | VNM | Vietnam | Potassic fertilizers | 200 | 0.18 | 0.17 | Very low |
| **Subtotal** | | | | **32,982** |  | | |

Data source: FAO.[FAOSTAT]. License: CC BY-NC-SA 3.0 IGO. Extracted from: [https://www.fao.org/faostat/en/#data]. Data of Access: [3-June-2022].

Fig. S3a. Dependency of trading partners on food imports from Ukraine in 2020. The y-axis shows the concentration of each commodity in trading partners’ markets by the Herfindahl–Hirschman Index, and the x-axis shows each trading partner’s import share of relevant commodity from Ukraine. The vertical color bar shows the import quantity (kt). Three-letter codes represent country names. The panels are divided into zones (Ⅰ–Ⅳ) that represent very high (I), high (II), low (III), and very low (IV) dependence on food imports from Russia and Ukraine. For a more detailed description, see Tables S2.

Fig. S3b. Dependency of trading partners on food imports from Ukraine in 2020. The y-axis shows the concentration of each commodity in trading partners’ markets by the Herfindahl–Hirschman Index, and the x-axis shows each trading partner’s import share of relevant commodity from Ukraine. The vertical color bar shows the import quantity (kt). Three-letter codes represent country names. The panels are divided into zones (Ⅰ–Ⅳ) that represent very high (I), high (II), low (III), and very low (IV) dependence on food imports from Russia and Ukraine. For a more detailed description, see Tables S2.

Fig. S3c. Dependency of trading partners on food imports from Ukraine in 2020. The y-axis shows the concentration of each commodity in trading partners’ markets by the Herfindahl–Hirschman Index, and the x-axis shows each trading partner’s import share of relevant commodity from Ukraine. The vertical color bar shows the import quantity (kt). Three-letter codes represent country names. The panels are divided into zones (Ⅰ–Ⅳ) that represent very high (I), high (II), low (III), and very low (IV) dependence on food imports from Russia and Ukraine. For a more detailed description, see Tables S2.

Fig. S3d. Dependency of trading partners on food imports from Russia in 2020. The y-axis shows the concentration of each commodity in trading partners’ markets by the Herfindahl–Hirschman Index, and the x-axis shows each trading partner’s import share of relevant commodity from Russia. The vertical color bar shows the import quantity (kt). Three-letter codes represent country names. The panels are divided into zones (Ⅰ–Ⅳ) that represent very high (I), high (II), low (III), and very low (IV) dependence on food imports from Russia and Ukraine. For a more detailed description, see Tables S3.

Fig. S3e. Dependency of trading partners on food imports from Russia in 2020. The y-axis shows the concentration of each commodity in trading partners’ markets by the Herfindahl–Hirschman Index, and the x-axis shows each trading partner’s import share of relevant commodity from Russia. The vertical color bar shows the import quantity (kt). Three-letter codes represent country names. The panels are divided into zones (Ⅰ–Ⅳ) that represent very high (I), high (II), low (III), and very low (IV) dependence on food imports from Russia and Ukraine. For a more detailed description, see Tables S3.

Fig. S3f. Dependency of trading partners on food imports from Russia in 2020. The y-axis shows the concentration of each commodity in trading partners’ markets by the Herfindahl–Hirschman Index, and the x-axis shows each trading partner’s import share of relevant commodity from Russia. The vertical color bar shows the import quantity (kt). Three-letter codes represent country names. The panels are divided into zones (Ⅰ–Ⅳ) that represent very high (I), high (II), low (III), and very low (IV) dependence on food imports from Russia and Ukraine. For a more detailed description, see Tables S3.

Fig. S3g. Dependency of trading partners on food imports from Ukraine in 2020. The y-axis shows the concentration of each commodity in trading partners’ markets by the Herfindahl–Hirschman Index, and the x-axis shows each trading partner’s import share of relevant commodity from Ukraine. The vertical color bar shows the import quantity (kt). Three-letter codes represent country names. The panels are divided into zones (Ⅰ–Ⅳ) that represent very high (I), high (II), low (III), and very low (IV) dependence on food imports from Russia and Ukraine. For a more detailed description, see Tables S2.

Fig. S3h. Dependency of trading partners on food imports from Ukraine in 2020. The y-axis shows the concentration of each commodity in trading partners’ markets by the Herfindahl–Hirschman Index, and the x-axis shows each trading partner’s import share of relevant commodity from Ukraine. The vertical color bar shows the import quantity (kt). Three-letter codes represent country names. The panels are divided into zones (Ⅰ–Ⅳ) that represent very high (I), high (II), low (III), and very low (IV) dependence on food imports from Russia and Ukraine. For a more detailed description, see Tables S2.

Fig. S3i. Dependency of trading partners on food imports from Ukraine in 2020. The y-axis shows the concentration of each commodity in trading partners’ markets by the Herfindahl–Hirschman Index, and the x-axis shows each trading partner’s import share of relevant commodity from Ukraine. The vertical color bar shows the import quantity (kt). Three-letter codes represent country names. The panels are divided into zones (Ⅰ–Ⅳ) that represent very high (I), high (II), low (III), and very low (IV) dependence on food imports from Russia and Ukraine. For a more detailed description, see Tables S2.

Fig. S3j. Dependency of trading partners on food imports from Russia in 2020. The y-axis shows the concentration of each commodity in trading partners’ markets by the Herfindahl–Hirschman Index, and the x-axis shows each trading partner’s import share of relevant commodity from Russia. The vertical color bar shows the import quantity (kt). Three-letter codes represent country names. The panels are divided into zones (Ⅰ–Ⅳ) that represent very high (I), high (II), low (III), and very low (IV) dependence on food imports from Russia and Ukraine. For a more detailed description, see Tables S3.

Fig. S3k. Dependency of trading partners on food imports from Russia in 2020. The y-axis shows the concentration of each commodity in trading partners’ markets by the Herfindahl–Hirschman Index, and the x-axis shows each trading partner’s import share of relevant commodity from Russia. The vertical color bar shows the import quantity (kt). Three-letter codes represent country names. The panels are divided into zones (Ⅰ–Ⅳ) that represent very high (I), high (II), low (III), and very low (IV) dependence on food imports from Russia and Ukraine. For a more detailed description, see Tables S3.

Fig. S3l. Dependency of trading partners on food imports from Russia in 2020. The y-axis shows the concentration of each commodity in trading partners’ markets by the Herfindahl–Hirschman Index, and the x-axis shows each trading partner’s import share of relevant commodity from Russia. The vertical color bar shows the import quantity (kt). Three-letter codes represent country names. The panels are divided into zones (Ⅰ–Ⅳ) that represent very high (I), high (II), low (III), and very low (IV) dependence on food imports from Russia and Ukraine. For a more detailed description, see Tables S3.

Fig. S4a. Dependency on Russian fertilizer imports in 2020. The y-axis shows the concentration of each commodity in trading partners’ markets by the Herfindahl–Hirschman Index, and the x-axis shows each trading partner’s share of total imports of fertilizers from Russia. The vertical color bar shows the import quantity (kt). Three-letter codes represent the country name. The panels are divided into zones (Ⅰ–Ⅳ) that represent very high (I), high (II), low (III), and very low (IV) dependence on fertilizer imports from Russia. For more details, see Tables S6.

Fig. S4b. Dependency on Russian fertilizer imports in 2020. The y-axis shows the concentration of each commodity in trading partners’ markets by the Herfindahl–Hirschman Index, and the x-axis shows each trading partner’s share of total imports of fertilizers from Russia. The vertical color bar shows the import quantity (kt). Three-letter codes represent the country name. The panels are divided into zones (Ⅰ–Ⅳ) that represent very high (I), high (II), low (III), and very low (IV) dependence on fertilizer imports from Russia. For more details, see Tables S6.

Fig. S4c. Dependency on Russian fertilizer imports in 2020. The y-axis shows the concentration of each commodity in trading partners’ markets by the Herfindahl–Hirschman Index, and the x-axis shows each trading partner’s share of total imports of fertilizers from Russia. The vertical color bar shows the import quantity (kt). Three-letter codes represent the country name. The panels are divided into zones (Ⅰ–Ⅳ) that represent very high (I), high (II), low (III), and very low (IV) dependence on fertilizer imports from Russia. For more details, see Tables S6.

Table S8. Comparison of import types, purchasing power parity (PPP) per capital, and population among trading partners of Ukraine and Russia in 2020.

| **Code** | **Importer** | **Import**  **types** | **PPP per capita**  **(constant 2017 international $)** | **Population**  **(millions)** | **Reference** |
| --- | --- | --- | --- | --- | --- |
| JOR | Jordan | 15 | 9,858 | 10 | [1] |
| LBN | Lebanon | 14 | 10,786 | 7 | [1] |
| MDA | Moldova | 14 | 11,859 | 3 | [1] |
| GEO | Georgia | 14 | 13,988 | 4 | [1] |
| CHN | China | 14 | 16,226 | 1,412 | [1] |
| BLR | Belarus | 14 | 19,176 | 9 | [1] |
| TUR | Turkey | 14 | 28,879 | 84 | [1] |
| DEU | Germany | 14 | 52,138 | 83 | [1] |
| EGY | Egypt | 13 | 12,801 | 101 | [1] |
| MYS | Malaysia | 13 | 26,319 | 33 | [1] |
| ISR | Israel | 13 | 39,754 | 9 | [1] |
| KOR | Korea | 13 | 42,442 | 52 | [1] |
| ARE | United Arab Emirates | 13 | 67,704 | 9 | [1] |
| POL | Poland | 12 | 32,599 | 38 | [1] |
| LTU | Lithuania | 12 | 37,238 | 3 | [1] |
| SAU | Saudi Arabia | 12 | 46,029 | 35 | [1] |
| NLD | Netherlands | 12 | 54,621 | 17 | [1] |
| MAR | Morocco | 11 | 7,817 | 36 | [1] |
| VNM | Vietnam | 11 | 10,351 | 98 | [1] |
| ZAF | South Africa | 11 | 12,640 | 60 | [1] |
| ARM | Armenia | 11 | 12,668 | 3 | [1] |
| AZE | Azerbaijan | 11 | 13,774 | 10 | [1] |
| BGR | Bulgaria | 11 | 22,608 | 7 | [1] |
| LVA | Latvia | 11 | 30,467 | 2 | [1] |
| OMN | Oman | 11 | 33,891 | 4 | [1] |
| PAK | Pakistan | 10 | 5,256 | 218 | [1] |
| MNG | Mongolia | 10 | 11,447 | 3 | [1] |
| THA | Thailand | 10 | 17,253 | 70 | [1] |
| KAZ | Kazakhstan | 10 | 25,172 | 19 | [1] |
| GRC | Greece | 10 | 27,005 | 11 | [1] |
| EST | Estonia | 10 | 35,969 | 1 | [1] |
| CYP | Cyprus | 10 | 39,660 | 1 | [1] |
| GBR | United Kingdom | 10 | 41,892 | 67 | [1] |
| NOR | Norway | 10 | 62,725 | 5 | [1] |
| CHE | Switzerland | 10 | 68,400 | 9 | [1] |
| KGZ | Kyrgyzstan | 9 | 4,747 | 7 | [1] |
| IND | India | 9 | 6,172 | 1,396 | [1] |
| UZB | Uzbekistan | 9 | 7,479 | 34 | [1] |
| TUN | Tunisia | 9 | 10,258 | 12 | [1] |
| DZA | Algeria | 9 | 10,575 | 44 | [1] |
| UKR | Ukraine | 9 | 12,513 | 41 | [1] |
| ROU | Romania | 9 | 29,120 | 19 | [1] |
| ESP | Spain | 9 | 36,036 | 47 | [1] |
| ITA | Italy | 9 | 39,149 | 60 | [1] |
| KWT | Kuwait | 9 | 41,006 | 5 | [1] |
| CAN | Canada | 9 | 46,508 | 38 | [1] |
| USA | United States | 9 | 60,205 | 331 | [1] |
| MOZ | Mozambique | 8 | 1,233 | 31 | [1] |
| ETH | Ethiopia | 8 | 2,687 | 100 | [1] |

**Table S8.** Continued.

| **Code** | **Importer** | **Import**  **types** | **PPP per capita**  **(constant 2017 international $)** | **Population**  **(millions)** | **Reference** |
| --- | --- | --- | --- | --- | --- |
| SEN | Senegal | 8 | 3,322 | 17 | [1] |
| TJK | Tajikistan | 8 | 3,678 | 9 | [1] |
| KEN | Kenya | 8 | 4,804 | 49 | [1] |
| BGD | Bangladesh | 8 | 6,058 | 165 | [1] |
| LKA | Sri Lanka | 8 | 13,096 | 22 | [1] |
| TKM | Turkmenistan | 8 | 15,606 | 6 | [1] |
| LBY | Libya | 8 | 15,719 | 7 | [1] |
| SRB | Serbia | 8 | 18,274 | 7 | [1] |
| CZE | Czech Republic | 8 | 38,802 | 11 | [1] |
| JPN | Japan | 8 | 40,015 | 126 | [1] |
| NZL | New Zealand | 8 | 40,727 | 5 | [1] |
| FRA | France | 8 | 43,874 | 65 | [1] |
| FIN | Finland | 8 | 47,416 | 6 | [1] |
| AUT | Austria | 8 | 52,395 | 9 | [1] |
| DNK | Denmark | 8 | 55,762 | 6 | [1] |
| QAT | Qatar | 8 | 91,541 | 3 | [1] |
| COD | Congo, Democratic Republic | 7 | 1,099 | 91 | [1] |
| BEN | Benin | 7 | 3,181 | 13 | [1] |
| COG | Congo, Republic | 7 | 4,170 | 5 | [1] |
| ECU | Ecuador | 7 | 10,403 | 18 | [1] |
| IDN | Indonesia | 7 | 11,586 | 270 | [1] |
| HUN | Hungary | 7 | 31,553 | 10 | [1] |
| BEL | Belgium | 7 | 49,222 | 12 | [1] |
| AUS | Australia | 7 | 49,342 | 26 | [1] |
| LBR | Liberia | 6 | 1,364 | 5 | [1] |
| MWI | Malawi | 6 | 1,384 | 21 | [1] |
| MDG | Madagascar | 6 | 1,483 | 27 | [1] |
| TGO | Togo | 6 | 2,065 | 8 | [1] |
| BFA | Burkina Faso | 6 | 2,103 | 22 | [1] |
| TZA | Tanzania | 6 | 2,774 | 58 | [1] |
| MMR | Myanmar | 6 | 4,969 | 53 | [1] |
| CIV | Côte d'Ivoire | 6 | 5,094 | 27 | [1] |
| NIC | Nicaragua | 6 | 5,389 | 6 | [1] |
| GHA | Ghana | 6 | 5,503 | 31 | [1] |
| ALB | Albania | 6 | 13,394 | 3 | [1] |
| MEX | Mexico | 6 | 8,157 | 128 | [1] |
| CRI | Costa Rica | 6 | 19,205 | 5 | [1] |
| SVK | Slovak | 6 | 31,798 | 5 | [1] |
| YEM | Yemen | 5 | 1,778 | 32 | [1] |
| AFG | Afghanistan | 5 | 2,329 | 33 | [1] |
| UGA | Uganda | 5 | 2,445 | 41 | [1] |
| CMR | Cameroon | 5 | 3,669 | 27 | [1] |
| NPL | Nepal | 5 | 3,762 | 29 | [1] |
| SDN | Sudan | 5 | 3,973 | 44 | [1] |
| WBG | State of Palestine | 5 | 5,087 | 5 | [1] |
| AGO | Angola | 5 | 6,025 | 33 | [1] |
| PHL | Philippines | 5 | 8,018 | 109 | [1] |
| IRQ | Iraq | 5 | 9,435 | 40 | [1] |
| BIH | Bosnia and Herzegovina | 5 | 13,972 | 3 | [1] |
| BRA | Brazil | 5 | 14,206 | 212 | [1] |
| MKD | North Macedonia | 5 | 15,986 | 2 | [1] |

**Table S8.** Continued.

| **Code** | **Importer** | **Import**  **types** | **PPP per capita**  **(constant 2017 international $)** | **Population**  **(millions)** | **Reference** |
| --- | --- | --- | --- | --- | --- |
| MUS | Mauritius | 5 | 20,220 | 1 | [1] |
| CHL | Chile | 5 | 22,032 | 19 | [1] |
| CHL | Chile | 5 | 22,032 | 19 | [1] |
| PAN | Panama | 5 | 25,605 | 4 | [1] |
| PRT | Portugal | 5 | 32,051 | 10 | [1] |
| SVN | Slovenia | 5 | 37,381 | 2 | [1] |
| MLT | Malta | 5 | 41,681 | 1 | [1] |
| SWE | Sweden | 5 | 51,983 | 10 | [1] |
| IRL | Ireland | 5 | 91,612 | 5 | [1] |
| GIN | Guinea | 4 | 2,462 | 14 | [1] |
| KHM | Cambodia | 4 | 4,472 | 16 | [1] |
| NGA | Nigeria | 4 | 4,917 | 206 | [1] |
| DJI | Djibouti | 4 | 5,245 | 1 | [1] |
| MRT | Mauritania | 4 | 5,772 | 4 | [1] |
| SLV | El Salvador | 4 | 8,267 | 6 | [1] |
| PER | Peru | 4 | 11,354 | 33 | [1] |
| GAB | Gabon | 4 | 15,119 | 2 | [1] |
| DOM | Dominican Republic | 4 | 17,653 | 10 | [1] |
| MNE | Montenegro | 4 | 18,280 | 1 | [1] |
| SYC | Seychelles | 4 | 27,213 | 0.10 | [1] |
| HKG | China, Hong Kong SAR | 4 | 56,301 | 7 | [1] |
| SGP | Singapore | 4 | 94,910 | 6 | [1] |
| BDI | Burundi | 3 | 732 | 12 | [1] |
| SOM | Somalia | 3 | 1,131 | 15 | [1] |
| ZWE | Zimbabwe | 3 | 1,991 | 15 | [1] |
| MLI | Mali | 3 | 2,123 | 21 | [1] |
| GMB | Gambia | 3 | 2,173 | 2 | [1] |
| HND | Honduras | 3 | 5,119 | 10 | [1] |
| BLZ | Belize | 3 | 7,220 | 0.42 | [1] |
| GTM | Guatemala | 3 | 7,869 | 18 | [1] |
| BOL | Bolivia | 3 | 7,882 | 12 | [1] |
| PRY | Paraguay | 3 | 12,153 | 7 | [1] |
| COL | Colombia | 3 | 13,642 | 50 | [1] |
| IRN | Iran | 3 | 14,991 | 84 | [1] |
| GUY | Guyana | 3 | 18,671 | 1 | [1] |
| ARG | Argentina | 3 | 19,680 | 45 | [1] |
| URY | Uruguay | 3 | 21,201 | 4 | [1] |
| RUS | Russian Federation | 3 | 26,721 | 146 | [1] |
| HRV | Croatia | 3 | 26,923 | 4 | [1] |
| NCL | New Caledonia | 3 | 41,025 | 0.27 | [1, 2] |
| BHR | Bahrain | 3 | 48,166 | 1 | [1] |
| ISL | Iceland | 3 | 52,410 | 0.36 | [1] |
| CAF | Central African Republic | 2 | 937 | 5 | [1] |
| NER | Niger | 2 | 1,221 | 24 | [1] |
| PRK | Korea, DPR | 2 | 1,584 | 26 | [1, 2] |
| SLE | Sierra Leone | 2 | 1,637 | 8 | [1] |
| RWA | Rwanda | 2 | 2,146 | 13 | [1] |
| ZMB | Zambia | 2 | 3,184 | 19 | [1] |
| CPV | Cabo Verde | 2 | 6,616 | 1 | [1] |
| SWZ | Eswatini | 2 | 8,686 | 1 | [1] |
| NAM | Namibia | 2 | 8,993 | 3 | [1] |

**Table S8.** Continued.

| **Code** | **Importer** | **Import**  **types** | **PPP per capita**  **(constant 2017 international $)** | **Population**  **(millions)** | **Reference** |
| --- | --- | --- | --- | --- | --- |
| DMA | Dominica | 2 | 10,064 | 0.07 | [1] |
| FJI | Fiji | 2 | 11,220 | 1 | [1] |
| BWA | Botswana | 2 | 13,546 | 3 | [1] |
| SUR | Suriname | 2 | 15,446 | 1 | [1] |
| BHS | Bahamas | 2 | 28,146 | 0.39 | [1] |
| SSD | South Sudan | 1 | 387 | 14 | [1] |
| VUT | Vanuatu | 1 | 2,554 | 0.30 | [1] |
| SYR | Syria | 1 | 2,618 | 21 | [1, 2] |
| COM | Comoros | 1 | 2,895 | 1 | [1] |
| MHL | Marshall Islands | 1 | 3,678 | 0.06 | [1] |
| VEN | Venezuela | 1 | 5,460 | 28 | [1] |
| LAO | Lao PDR | 1 | 7,633 | 7 | [1] |
| JAM | Jamaica | 1 | 9,488 | 3 | [1] |
| CUB | Cuba | 1 | 12,085 | 11 | [1, 2] |
| VCT | Saint Vincent and  the Grenadines | 1 | 12,941 | 0.11 | [1] |
| BRB | Barbados | 1 | 13,458 | 0.29 | [1] |
| COK | Cook Islands | 1 | 14,349 | 0.02 | [1, 2] |
| GRD | Grenada | 1 | 14,480 | 0.11 | [1] |
| VGB | British Virgin Islands | 1 | 16,633 | 0.03 | [1, 2] |
| GNQ | Equatorial Guinea | 1 | 17,174 | 1 | [1] |
| ATG | Antigua and Barbuda | 1 | 17,299 | 0.10 | [1] |
| MDV | Maldives | 1 | 18,262 | 0.38 | [1] |
| PYF | French Polynesia | 1 | 18,582 | 0.30 | [1] |
| KNA | Saint Kitts and Nevis | 1 | 22,102 | 0.06 | [1] |
| FRO | Faeroe Islands | 1 | 41,288 | 0.05 | [1] |
| CYM | Cayman Islands | 1 | 67,093 | 0.07 | [1] |
| ATG | Antigua and Barbuda | 1 | 17,299 | 0.10 | [1] |
| MDV | Maldives | 1 | 18,262 | 0.38 | [1] |
| PYF | French Polynesia | 1 | 18,582 | 0.30 | [1, 2] |
| KNA | Saint Kitts and Nevis | 1 | 22,102 | 0.06 | [1] |
| FRO | Faeroe Islands | 1 | 41,288 | 0.05 | [1, 2] |
| CYM | Cayman Islands | 1 | 67,093 | 0.07 | [1] |

Data source: [1]International Monetary Fund, World Economic Outlook Database, April 2023, https://www.imf.org/en/Publications/WEO/weo-database/2023/April

[2]Central Intelligence Agency, Real GDP (purchasing power parity), The World Factbook, April 2023, https://www.cia.gov/the-world-factbook/field/real-gdp-purchasing-power-parity
